# LacZ‐reporter mapping of *Dlx5*/*6* expression and genoarchitectural analysis of the postnatal mouse prethalamus

**DOI:** 10.1002/cne.24952

**Published:** 2020-06-18

**Authors:** Luis Puelles, Carmen Diaz, Thorsten Stühmer, José L. Ferran, Margaret Martínez‐de la Torre, John L. R. Rubenstein

**Affiliations:** ^1^ Department of Human Anatomy and Psychobiology and IMIB‐Arrixaca Institute University of Murcia Murcia Spain; ^2^ Department of Medical Sciences, School of Medicine and Institute for Research in Neurological Disabilities University of Castilla‐La Mancha Albacete Spain; ^3^ Nina Ireland Laboratory of Developmental Neurobiology, Department of Psychiatry UCSF Medical School San Francisco California USA

**Keywords:** distalless, genoarchitectural analysis, liminar alar domain, mouse, pregeniculate nucleus, preincertal nucleus, prethalamus, reticular nucleus, subgeniculate nucleus, zona incerta, zona limitans rostral shell

## Abstract

We present here a thorough and complete analysis of mouse P0‐P140 prethalamic histogenetic subdivisions and corresponding nuclear derivatives, in the context of local tract landmarks. The study used as fundamental material brains from a transgenic mouse line that expresses *LacZ* under the control of an intragenic enhancer of *Dlx5* and *Dlx6* (*Dlx5*/*6‐LacZ*). Subtle shadings of LacZ signal, jointly with pan‐DLX immunoreaction, and several other ancillary protein or RNA markers, including *Calb2* and *Nkx2.2* ISH (for the prethalamic eminence, and derivatives of the rostral zona limitans shell domain, respectively) were mapped across the prethalamus. The resulting model of the prethalamic region postulates tetrapartite rostrocaudal and dorsoventral subdivisions, as well as a tripartite radial stratification, each cell population showing a characteristic molecular profile. Some novel nuclei are proposed, and some instances of potential tangential cell migration were noted.

## Graphical abstract

Prosomeric mappings of *Dlx5*/*6‐LacZ* expression plus relevant gene/protein markers delineated genoarchitectural subdivisions of the adult mouse prethalamus, identified boundaries with hypothalamus, thalamus, and underlying tegmental field, and produced an advanced morphologic model of the mammalian prethalamus.

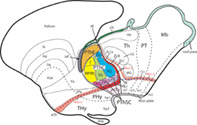

**Abbreviations**
1, PG: layer 1
2, PG: layer 2
3, PG: layer 3
4, PG: layer 4
A: anterior thalamic complex
a/b: alar/basal boundary
ac: anterior commissure
AD: anterodorsal thalamic nucleus
AH: anterior hypothalamic area
al: ansa lenticularis
AM: anteromedial thalamic nucleus
Ar: arcuate nucleus
AV: anteroventral thalamic nucleus
BAC: bed nucleus of the anterior commissure
Bas: basal plate
BSM: bed nucleus of the stria medullaris
BST: bed nucleus of the stria terminalis
BSTL: lateral part of BST
BSTM: medial part of BST
cch: optic chiasma
ch: chorioid tela
CLi: longitudinal liminar portion of the ZLC
CM: central medial thalamic nucleus
cp: posterior commissure
CPv: periventricular stratum of PThC
Dg: diagonal area
DMH: dorsomedial hypothalamic nucleus
E: epiphysis
e: external layer of PG (Layer 2)
EPA: entopeduncular accessory nucleus
EPD: dorsal entopeduncular nucleus
EPV: ventral entopeduncular nucleus
F: Forel's fields
f: fornix
fi: fimbria
GP: globus pallidus
GPE: globus pallidus externus
GPI: globus pallidus internus
Hb: habenula
Hi: hippocampus
hp1: hypothalamic prosomere 1
hp2: hypothalamic prosomere 2
i: internal layer of PG (Layer 4)
ic: internal capsule
IGL: intergeniculate leaflet
IMD: intermediodorsal thalamic nucleus
IP: interpeduncular nucleus
ivf: interventricular foramen
LD: laterodorsal thalamic nucleus
LG: lateral geniculate nucleus
LHb: lateral habenular nucleus
M: mamillar area
m1: mesomere 1
m2: mesomere 2
Mb: midbrain
MD: mediodorsal thalamic nucleus
MG: medial geniculate nucleus
MHb: medial habenular nucleus
ML: lateral mamillar nucleus
ml: medial lemniscal tract
MM: medial mamillar nucleus
mp: mammillary peduncle
MTh: nucleus of the mamillothalamic tract
mth: mamillothalamic tract
mtg: mamillotegmental tract
NHy: neurohypophysis
nst: nigrostriatal tract
och: optic chiasma
ot: optic tract
Ov: oval nucleus
p1: prosomere 1
p2: prosomere 2
p3: prosomere 3
p1Tg: p1 tegmentum
p2Tg: p2 tegmentum
p3Tg: p3 tegmentum
Pa: paraventricular hypothalamic complex
Pal: pallidum
Pall: pallium
PaXi: paraxiphoid nucleus
pc: posterior commissure
pe: cerebral peduncle
Pf: parafascicular thalamic nucleus
PG: pregeniculate nucleus
PGI: intermediate stratum of PG
PGPv: periventricular stratum of PG
PHy: peduncular hypothalamus
PI: preincertal nucleus (preincertal formation, intermediate stratum)
PIPv: preincertal formation, periventricular stratum
PIS: preincertal formation, superficial stratum
PMC: premamillary ventral nucleus
Pn: pontine nuclei
POA: preoptic area
PP: peripeduncular nucleus
PPa: peduncular part of Pa
PRt: perireticular nucleus
PSPa: peduncular part of SPa
PT: pretectum
PTh: prethalamus
PThC: central prethalamic region
PThE: prethalamic eminence
PThE (vz): ventricular zone of PThE
PThSC: subcentral prethalamic region
PVA: paraventricular thalamic nucleus, anterior
Re: reuniens thalamic nucleus
rf: retroflex fascicle
RI: retroincertal nucleus
RIS: superficial RI
RLi: longitudinal liminar portion of ZLR
RM: retromamillar area
RML: lateral retromamillar nucleus
RMM: medial retromamillar nucleus
RP: retropeduncular (reticular complex, superficial stratum)
RPm: marginal migrated RP cells
Rt: reticular nucleus (reticular complex, intermediate stratum)
RtPv: reticular complex, periventricular stratum
SCh: suprachiasmatic nucleus
SCPv: periventricular stratum of PThSC
Se: septum
SG: subgeniculate nucleus (subgeniculate complex, superficial stratum)
SGI: subgeniculate complex, intermediate stratum
SGL: subgeniculate lamina
SGPv: subgeniculate complex, periventricular stratum
SPa: subparaventricular hypothalamic complex
sm: stria medullaris
SNC: substantia nigra compacta
SNL: substantia nigra lateral
SNR: substantia nigra reticular
St: striatum
st: stria terminalis
STh: subthalamic nucleus
STMA: bed nucleus of the stria terminalis, medial division, anterior part
Subpall: subpallium
T: triangular nucleus (Rt shell)
Tel: telencephalon
Th: thalamus
tht: tectothalamic tract
THy: terminal hypothalamus
TM: tuberomamillar area
TPa: terminal part of Pa
TS: triangular septal nucleus
TSPa: terminal part of SPa
v: ventricle
VA: ventral anterior thalamic nucleus
VM: ventromedial thalamic nucleus
VMH: ventromedial hypothalamic nucleus
VPa: ventral part of the peduncular paraventricular domain
VPL: ventroposterior lateral thalamic nucleus
VPM: ventroposterior medial thalamic nucleus
vt: velum transversum
vz: ventricular zone
Xi: xiphoid nucleus
ZI: zona incerta
ZIC: zona incerta caudal, intermediate stratum
ZICS: superficial stratum of ZIC
ZICPv: periventricular stratum of ZIC
ZIPv: zona incerta periventricular
ZIR: zona incerta rostral, intermediate stratum
ZIRS: superficial stratum of ZIR
ZIRPv: periventricular stratum of ZIR
ZIS: zona incerta superficial
ZL: interthalamic zona limitans
ZLC: caudal shell of ZL
ZLCo: core of ZL
ZLR: rostral shell of ZL
ZLRC: cap portion of ZLR
ZLRI: intermediate portion of ZLR
ZLRPv: periventricular portion of ZLR

## 1 INTRODUCTION

**FIGURE 1:**
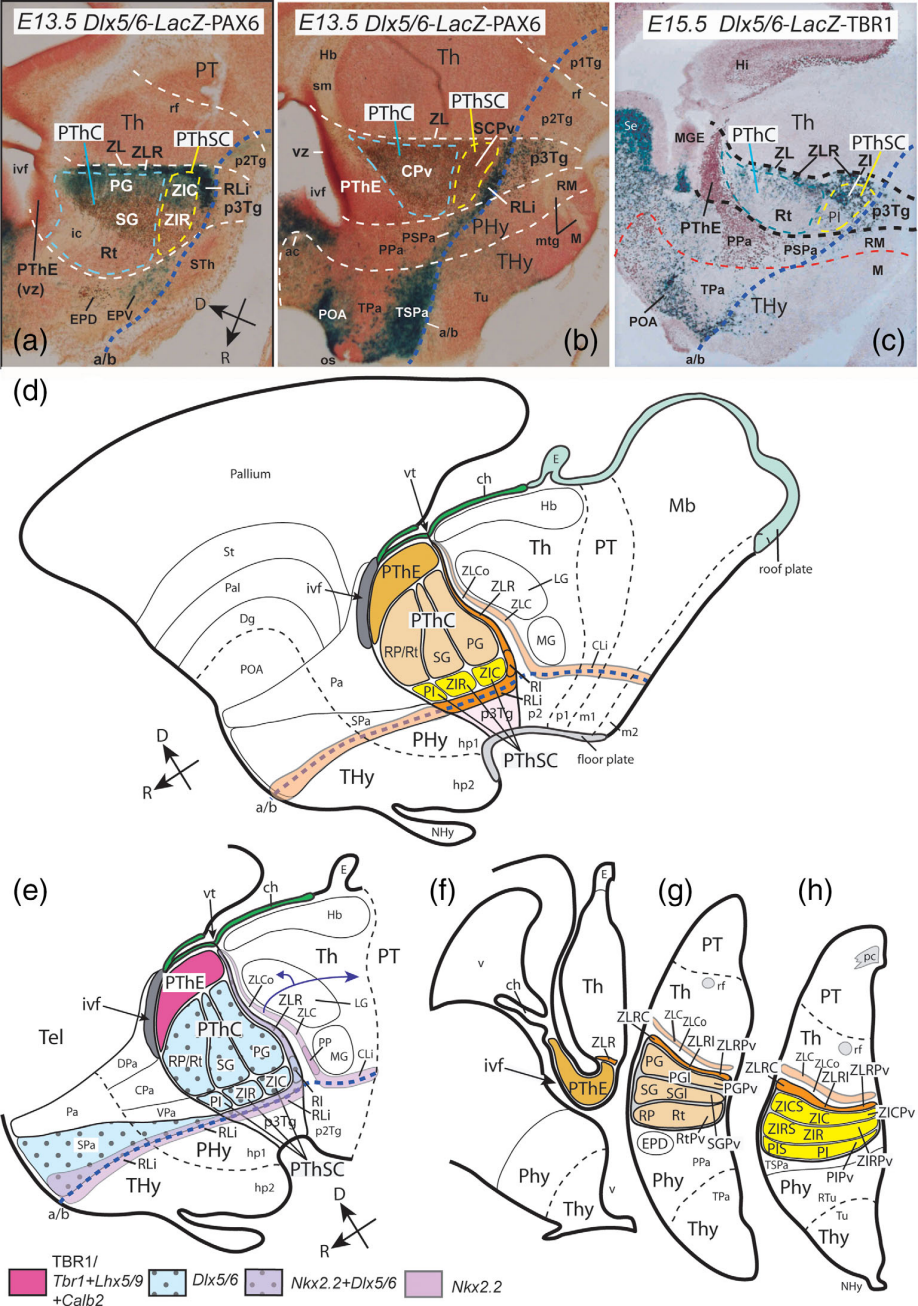
Dorsoventral, rostrocaudal, and radial subdivisions of the mouse prethalamus at embryonic stages. (a–c) Lateral and more medial sagittal sections of an E13.5 mouse brain carrying the *Dlx5*/*6*‐*LacZ* construct (blue), plus PAX6 immunoreaction (brown) (a,b), and similar section at E15.5 labeled with *Dlx5*/*6‐LacZ* and TBR1 immunoreaction (c). The rostral (R) and dorsal (D) spatial directions are indicated in (a). White dash lines indicate the transverse pretecto‐thalamic, thalamo‐prethalamic and prethalamo‐hypothalamic interprosomeric boundaries. The dark blue dash line indicates the alar/basal limit (a/b). The central and subcentral prethalamic regions (PThC, PThSC) are delineated respectively with light blue and yellow dash lines; the main derived primordia are identified (see abbreviation list) (a–c). Note the *Dlx5*/*6‐LacZ*‐negative prethalamic eminence (PThE), lying immediately under the chorioidal roof (ch; green in d,e) expresses PAX6 and TBR1 in its ventricular and mantle zones, respectively. *Dlx5*/*6*‐*LacZ* signal is mainly restricted to the underlying PThC and PThSC subdivisions, but appears also at the rostral shell of the zona limitans (ZLR) and its rostral liminar extension (RLi) along the alar‐basal boundary (a,b). The *Dlx5*/*6‐LacZ* signal is relatively weaker at rostral prethalamic levels (a‐c). (d) Schema of the prosomeric model illustrating the main neuromeric transverse subdivisions and the dorsoventrally disposed, longitudinal roof, alar, basal and floor plates. The midbrain is subdivided rostrocaudally into mesomeres m1 and m2, while the diencephalon divides caudorostrally into diencephalic prosomeres p1, p2, and p3, and the secondary prosencephalon likewise into hypothalamo‐telencephalic prosomeres hp1 and hp2. Black dash lines indicate the transverse interneuromeric limits. The longitudinal a/b boundary is represented as a dark‐blue dash line. The main subdivisions of the prethalamus are highlighted and their relationship with thalamic and hypothalamic neighbor structures are illustrated, based on the embryonic material in (a‐c). The prethalamus (alar plate of p3) is divided dorsoventrally into PThE (sienna background), PThC (light orange), PThSC (yellow) subregions, and we see as well the partly caudal and partly ventral ZLR/RLi complex (strong orange). Note a rostral part of the PThE ventricular surface bulges into the interventricular foramen (ivf; dark gray background; this implies partial evagination of PThE into caudomedial wall of hemisphere, under the chorioidal fissure—not shown). PThC and PThSC are rostrocaudally subdivided into three entities. The PThC contains the reticular/retropeduncular, subgeniculate and pregeniculate radial complexes (RP/Rt, SG, PG) whereas the PThSC contains the preincertal, rostral zona incerta and caudal zona incerta radial complexes (PI, ZIR, ZIC). The prethalamic basal plate, or p3 tegmentum (p3Tg), is colored in light pink. PThE, PG and ZIC contact caudally with the ZLR (strong orange), whereas PThE, RP/Rt and PI bound rostrally with the hypothalamic (alar) paraventricular domain (Pa); instead, the RLi continues rostrally into the rostrally expanding hypothalamic subparaventricular domain (SPa). The thin black dash line in the hypothalamus marks the intrahypothalamic transverse boundary between hp1 and hp2 prosomeres; a similar dash line also marks the thalamo‐pretectal interprosomeric limit between thalamus and pretectum and corresponding tegmentum (p2Tg). (e) Schematic color‐coded map based on d of some of the gene patterns expressed in the prethalamus. Note *Nkx2.2* is primarily expressed only at the ZLR and RLi, overlapping with *Dlx5*/*6‐LacZ* signal*. Nkx2.2* is also expressed at the caudal zona limitans shell area (ZLC) and its caudal liminar extension (CLi; this reaches the isthmic boundary). It is still unclear whether the *Nkx2.2*‐positive forebrain liminar band (RLi and CLi) lies in the alar plate, in the basal plate, or halfway across the alar‐basal boundary (the option tentatively illustrated here). Blue arrows indicate documented tangential migratory routes of GABAergic cells, originated in the ZLC, reaching the thalamic lateral geniculate nucleus (LG) and the posterior limitans nucleus in the caudal thalamus (Delogu et al., 2012; Golding et al., 2014). Analogous migrations from ZLC and/or ZLR eventually enter the PThC. (f–h) Three horizontal schemata in dorsoventral order based on sections through PThE (f), PThC (g) and PThSC (h) subregions of an E13.5 embryo. The three dorsoventral prethalamic subdivisions are highlighted using the same color code as in (d) [Color figure can be viewed at wileyonlinelibrary.com]

We offer here a molecular (genoarchitectural) analysis of mouse prethalamus structure, interpreted within the updated prosomeric model (Puelles, 1995, 2001a, 2013; Puelles, Martinez‐de‐la‐Torre, Bardet, & Rubenstein, 2012; Puelles & Rubenstein, 1993, 2003, 2015). The prethalamus used to be known as the “ventral thalamus” in classic neuroanatomic works. The old terms referring to a dorsal/ventral division of the thalamus are meaningful only within the columnar morphological model of the forebrain, and they refer to the ad hoc forebrain axis defined in that model, held to end in the telencephalon (Herrick, 1910; Kuhlenbeck, 1973; Swanson, 2012, 2018). This traditional view is now superseded by the prosomeric model, where these regions jointly with the pretectum are held to be *caudorostral* components of the diencephalic alar plate (p1–p3 in caudorostral order; Figure 1) on the basis of substantial molecular and experimental evidence supporting a forebrain axis ending in the hypothalamus (Puelles & Rubenstein, 2015).

The updated prosomeric model defines the diencephalon as a forebrain sector intercalated rostrocaudally between the hypothalamus (divided into hypothalamic prosomeres hp1 and hp2, in caudorostral order) and the midbrain (mesomeres m1 and m2, in rostrocaudal order; Figure 1d). The diencephalon is itself divided into three diencephalic prosomeres (p1–p3 in caudorostral order; note p3 contacts rostrally hp1, whereas p1 contacts caudally m1; m2 contacts the isthmic hindbrain; Figure 1d). All prosomeres display four fundamental longitudinal or dorsoventral zones: the floor, basal, alar and roof plates of His (1893a, 1893b). In the diencephalon, the alar domains of p1‐p3 were given simplified novel names: pretectum (p1), thalamus (p2) and prethalamus (p3; Puelles & Rubenstein, 2003), thus evading wrong columnar axial connotations. The classic “dorsal thalamus” and “ventral thalamus” were simply renamed “thalamus” and “prethalamus,” respectively. This makes our terminological usage consistent with the well‐known anteroposterior patterning effects of the interposed zona limitans intrathalamica (ZL; Figure 1d), and ties in with accepted analogous semantic usage with the “tectum” (midbrain alar plate) and “pretectum” (alar part of caudal diencephalic prosomere). The columnar term “epithalamus” is not problematic in the change to the prosomeric paradigm, since we conceive this area as lying hyperdorsally *within* the “thalamus” (i.e., it is restricted in extent to the middle or thalamic diencephalic prosomere, rather than being a general column of the diencephalon). A separate but comparable hyperdorsal domain appears at the top of the “prethalamus” (rostral diencephalic prosomere); this was misidentified classically as “eminentia thalami,” a term making reference to its bulge at the back of the interventricular foramen. Its wrong historic ascription to the thalamus was due to initial lack of distinction between “ventral” and “dorsal” parts of thalamus. Hayes, Murray, and Jones (2003) reasonably proposed to rename this as “prethalamic eminence,” an option adopted thereafter in the prosomeric model.

We first approach genoarchitectural analysis of the prethalamus by a detailed study of differential expression of members of the family of *Dlx* genes. Four *Dlx* homeobox genes (*Dlx1*,*2*,*5*,*6*) are expressed in the embryonic mouse forebrain, as was described in the early 1990s (see historic account and criticism in Bulfone et al., 1993; Price, Lemaistre, Pischetola, Di Lauro, & Duboule, 1991; Robinson, Wray, & Mahon, 1991; Simeone et al., 1994). There are important differences in the relative timing of their respective expression along the process of neuronal differentiation. In the telencephalic subpallium, *Dlx* gene and DLX protein expression cellular patterns appear to be largely indistinguishable. *Dlx2* expression begins in scattered cells in the ventricular zone (VZ) and is followed by *Dlx1*; both are then most highly expressed in secondary progenitors in the subventricular zone (SVZ), where *Dlx5* and *Dlx6* expression is subsequently initiated, co‐expressed with *Dlx1* and *Dlx2* (Eisenstat et al., 1999; Lindtner et al., 2019; Liu, Ghattas, Liu, Chen, & Rubenstein, 1997). *Dlx*/DLX expression patterns in postmitotic neurons are more diverse. For instance, parvalbumin‐positive cortical interneurons distinctly express *Dlx2* and *Dlx5* but show much lower levels of *Dlx1* (Cobos, Broccoli, & Rubenstein, 2005). In the amygdala, *Dlx1* and *Dlx2* are expressed by the intercalated nuclei, whereas *Dlx5* and *Dlx6* are expressed in the central nucleus (Wang, Lufkin, & Rubenstein, 2011).

Regionally, there are three forebrain *Dlx* expression domains, shared by all four *Dlx* paralog genes at least transiently (Akimenko, Ekker, Wegner, Lin, & Westerfield, 1994; Brox, Puelles, Ferreiro, & Medina, 2003; Bulfone et al., 1993; Eisenstat et al., 1999; Ellies et al., 1997; Hauptmann & Gerster, 2000; Liu et al., 1997; Medina, Brox, Legaz, García‐López, & Puelles, 2005; Mueller, Wullimann, & Guo, 2008; Myojin et al., 2001; Neidert, Virupannavar, Hooker, & Langeland, 2001; Papalopulu & Kintner, 1993; Puelles et al., 2000; Puelles, Martinez, Martinez‐de‐la‐Torre, & Rubenstein, 2004; Simeone et al., 1994; Smith‐Fernandez, Pieau, Repérant, Boncinelli, & Wassef, 1998). The earliest expression domain appears around E10 in a well‐delimited longitudinal sector of the rostral alar forebrain, which encompasses a ventral part of the prospective alar prethalamus and extends rostralward into the subparaventricular alar hypothalamic area (Puelles, Martinez‐de‐la‐Torre, Bardet, et al., 2012). Around E11, a second more dorsal *Dlx* expression domain defines the subpallial telencephalon. This bridges the telencephalic stalk, and jointly characterizes all subpallial subdomains in the ganglionic eminences (prospective striatum, pallidum, diagonal area, and the unevaginated preoptic area; this territory includes the bed nuclei of the stria terminalis, part of subpallial amygdala, as well as a major subpallial part of the septum; Puelles, 2017; Puelles, Harrison, Paxinos, & Watson, 2013; Puelles et al., 2000; Puelles et al., 2004; Puelles & Rubenstein, 2003). A third *Dlx* expression domain appears subsequently within the tuberal/retrotuberal region of the basal hypothalamus (Puelles, Martinez‐de‐la‐Torre, Bardet, et al., 2012). The subpallial and alar hypothalamic *Dlx*‐expressing domains are separated by a longitudinal *Dlx*‐negative gap which is the site where the supraopto‐paraventricular hypophysotropic complex is formed; this domain limits caudally with the prethalamic *Dlx* expression domain and the related prethalamic eminence (Puelles, Martinez‐de‐la‐Torre, Bardet, et al., 2012). While knowledge on the alar ganglionic derivatives of the *Dlx*‐positive subpallial telencephalon and the alar subparaventricular and basal tuberal hypothalamus has increased in recent years (Puelles, 2017; Puelles et al., 2000; Puelles et al., 2013; Puelles, Martinez‐de‐la‐Torre, Bardet, et al., 2012; Silberberg et al., 2016; Stühmer, Puelles, Ekker, & Rubenstein, 2002; Zerucha et al., 2000), a detailed analysis of the mature derivatives of the mouse extra‐telencephalic *Dlx* domains, and particularly its diencephalic part (prethalamus), is not yet available.

The diencephalic (prethalamic) *Dlx* domain (alar p3) ends caudally at the zona limitans (the transverse interthalamic boundary separating the thalamus from the prethalamus, revealed to represent a mid‐diencephalic secondary organizer; review in Puelles & Martinez, 2013). Rostrally the prethalamus abuts the *Dlx*‐negative hypothalamic paraventricular domain. Topologically dorsally within the alar plate, there is the prethalamic eminence, where *Dlx* genes are not expressed (this dorsal subregion has a confusing *rostral topography*, due to the axial bending at the cephalic flexure; it expresses differentially the calretinin protein as well as RNA encoded by *Tbr1*, *Gdf10*, *Lhx5* and *Lhx9*) (Abellán, Vernier, Rétaux, & Medina, 2010; Puelles et al., 2000; Puelles, Martinez‐de‐la‐Torre, Bardet, et al., 2012; Shimogori et al., 2010). Ventrally to the eminentia the *Dlx*‐positive prethalamus reaches the alar‐basal boundary with a *Dlx*‐positive subregion encompassing both the zona incerta and a new longitudinal element to be described below, the rostral liminar area (Puelles, Martinez‐de‐la‐Torre, Bardet, et al., 2012; Puelles & Rubenstein, 2003, 2015). Altogether, the diencephalic *Dlx*‐positive domain clearly encompasses most prethalamic nuclear primordia, excepting the prethalamic eminence (Diez‐Roux et al., 2011; Jones & Rubenstein, 2004; Kitamura, Miura, Yanazawa, Miyashita, & Kato, 1997; Puelles & Rubenstein, 2003).

The literature lacks a complete and widely accepted list of mammalian prethalamic derivatives. Some of them seem to have been ascribed to either thalamus or subthalamus (see critique of the latter obsolete concept in Puelles, Martinez‐de‐la‐Torre, Bardet, et al., 2012), and both the transversal prethalamo‐hypothalamic boundary and the longitudinal alar‐basal boundary separating alar prethalamus from its corresponding tegmental domain are rather vaguely defined. There is indeed considerable uncertainty in the literature about where precisely some populations present in this area originate; for example, zona incerta, reticular nucleus, intergeniculate leaflet, and retropeduncular nucleus (see Section 4). Throwing light on this issue is the primary aim of the present report.

We mainly made use of mice (*Mus musculus*) transgenically modified to express the reporter bacterial enzyme beta‐galactosidase (*LacZ*) under control of a zebrafish enhancer (Zerucha et al., 2000). Once inserted into the mouse genome, this enhancer imitates the activity of its mouse homolog, the *Dlx5*/*6* enhancer, driving *LacZ* expression only in mouse cells which normally express *Dlx* genes, in a pattern closely resembling that of *Dlx5* and *Dlx6* (Stühmer, Puelles, et al., 2002; Zerucha et al., 2000). This is consistent with the fact that the homologous mouse *Dlx5*/6 (I56i) and zebrafish *zfdlx4*/*6* enhancers are ultraconserved, showing very little divergence across vertebrate evolution (Zerucha et al., 2000).

After beta‐galactosidase histochemical reaction, the cells expressing (or having once expressed) *Dlx5*/*6‐LacZ* continue to be labeled by beta‐galactosidase activity in the cytoplasm, demonstrable as a blue labeling product, which in most places remains visible until adulthood (e.g., in present results up to P140).

We essentially mapped the blue‐labeled cells as far as possible during postnatal development (P0–P140), identifying in sagittal, coronal and horizontal section planes the successive changes in their distribution, as well as their eventual relationships with recognizable mature nuclei. We further correlated these mapping results with various other differential markers aiding a tridimensional systematization of prethalamic derivatives (i.e., along dorsoventral, anteroposterior and radial dimensions). In order to check comparatively the derivatives of the *Dlx*‐positive prethalamic nuclei we studied postnatal RNA expression of *Calb1*, *Calb2*, *Ecel1*, *Enc1*, *Islet1*, *Nkx2.2*, *Pax6*, *Six3*, and *Somatostatin*, as well as Calbindin (CB), Neuropeptide Y (NPY), Parvalbumin (PV), and PAX6 proteins.

The *Dlx5*/*6‐LacZ* reaction was also compared with pan‐DLX immunohistochemistry (DLX1,2,5,6 proteins are all reactive to this antibody, if present). We made the surprising observation that differential patterns could be observed in specific sets of prethalamic derivatives (e.g., some nuclei showed only *LacZ* signal, both *LacZ* and immunoreaction, or only immunoreaction). After checking corresponding *Dlx1*/*2*/*5*/*6* ISH data in the Allen Adult and Developing Mouse Brain Atlas, these findings were interpreted as evidence that different *Dlx* paralogues predominate in given prethalamic nuclei at postnatal stages, similarly as was found previously in the subpallium (as noted above). Our anatomic analysis and terminology was guided by the most recent, updated version of the prosomeric forebrain model (Puelles, 2013; Puelles, Martinez‐de‐la‐Torre, Bardet, et al., 2012; Puelles, Martinez‐de‐la‐Torre, Ferran, & Watson, 2012; Puelles & Rubenstein, 2015).

## 2 MATERIALS AND METHODS

### 2.1 Animals

All experimental procedures with transgenic mice were approved by the Committee on Animal Research at University of California, San Francisco (CA), and mouse colonies maintained in accordance with National Institutes of Health and UCSF guidelines.

All experimental protocols, handling use and care of nontransgenic mice were conducted in compliance with the current normative standards of the European Community (86/609/EEC), the Spanish Government (Royal Decree, 1201/2005; Law 32/2007) and the approval of University of Murcia Committee for Animal Experimental Ethics.

For the present research, mice (transgenic or not; *Mus musculus*) were collected from postnatal to adult stages (embryonic specimens were collected as well) (Table 1).

**TABLE 1:** Summary of numbers of transgenic and nontransgenic animals at embryonic and postnatal stages, and markers used in each case

	Stage								
	E12.5	E13.5	E14.5	E15.5	E16.5	E18.5	P0–P8	P15–P40	P140
**Transgenic animals**									
*Dlx5*/*6*‐*lacZ*							Hor (*n* = 2); Sag (*n* = 2)	Hor (*n* = 2) Sag (*n* = 2)	Hor (*n* = 1) Sag (*n* = 1)
*Dlx5*/*6*‐*lacZ* +DLX							Sag (*n* = 1)		
*Dlx5*/*6*‐*lacZ* +PV							Hor (*n* = 1) Sag (*n* = 1)		
*Dlx5*/*6‐lacZ* +TBR1				Sag (*n* = 1)					
*Dlx5*/*6*‐*lacZ* +PAX6		Sag (*n* = 1)			Sag (*n* = 1)				
*Dlx5*/*6*‐*lacZ* +NPY							Sag (*n* = 2)		
*Dlx5*/*6*‐*lacZ* +CB							Sag (*n* = 4)		
*Nkx2.2*+*Otp‐LacZ*	Sag (*n* = 1)	Sag (*n* = 1)	Sag (*n* = 1)						
**Nontransgenic animal**									
*Calb1+*CR							Hor (*n* = 1)		
*Calb2*+ CB/NOS/TH								Hor (*n* = 1)	
*Enc1*+CB						Sag (*n* = 1)			
*Enc1*+CR						Hor (*n* = 1)			
*Nkx2.2*+CB/TH							Hor (*n* = 8) Trans (*n* = 2)		

Abbreviations: CB, calbindin; CR, calretinin; DLX, distalless homeobox protein; Hor, horizontal sections through the prethalamus; *n*, number of cases; NOS, nitric oxide synthase; NPY, neuropeptide Y; PAX6, paired box protein 6; PV, parvalbumin; Sag, sagittal sections; TBR1, T‐box, brain, 1 protein; TH, tyrosine hydroxylase; Trans, transversal sections through the prethalamus.

### 2.2 Transgenic animals

The isolation of zebrafish *Dlx5*/*6* forebrain enhancer elements, construction of the zfdlx5/6‐*LacZ* transgenic vector, and the characterization of the *LacZ* expression pattern in transgenic mice (strain C57 Bl/6) with respect to that of the endogenous *Dlx5* and *Dlx6* genes, are described in Zerucha et al. (2000). The propagation of the transgene appears to be stable, as no change in the pattern or intensity of β‐galactosidase expression has been noted for more than 20 generations. Heterozygous *Dlx5*/*6*‐*LacZ* brain specimens were fixed and processed histochemically (with or without immunochemical counterstains) at stages P0, P4, P15, P40, and P140.

### 2.3 Preparation of tissue

Most of analyzed brains were from postnatal animals (*n* = 32) although few embryonic brains (*n* = 5) were also included in this study. For the preparation of embryonic brain tissue, timed‐pregnant dams were killed by cervical dislocation, embryos removed and the brains dissected in cold phosphate‐buffered saline (PBS: 137 mM NaCl, 2.7 mM KCl, 1.8 mM KH_2_PO_4_, 5.1 mM Na_2_HPO_4_, pH 7.4). The tissue was fixed in cold 4% paraformaldehyde/PBS for between 30 min (embryonic day [E] 13.5) to 2 hr (E15.5 and later stages). For the preparation of postnatal and adult tissue, animals were anesthetized with 2% chloral hydrate/PBS, and cardially perfused with between 10 ml (postnatal day [P] 0) and 50 ml (P13 and older) cold 4% paraformaldehyde/PBS. Following dissection, brains were postfixed in the same solution for 3 hr to overnight. All tissues were either sectioned directly or stored at −20°C in a solution of 30% vol/vol ethylene glycol, 30% vol/vol glycerol, 0.4 × PBS. Brains stored in this way were rinsed overnight in PBS prior to sectioning.

### 2.4 Sectioning of tissue

The brains from transgenic animals were parted along the midline to obtain transversal and sagittal sections from the same specimen. The rest of brains were sectioned as a whole. For sectioning, the embedded tissue was oriented to obtain either sagittal, horizontal or transverse sections across the prethalamus, and cut into series of 100 μm‐thick slices with a vibrating blade microtome (Leica). Sections from *Dlx5*/*6‐LacZ* transgenic brains were immediately used for the X‐gal staining reaction, and some of them processed for in situ hybridization or immunohistochemistry. Sections from nontransgenic brains were processed for in situ hybridization and/or immunohistochemistry.

### 2.5 X‐gal staining

Sections were immersed in a solution of 10 mM Tris–HCl (pH 7.3), 0.005% sodium‐desoxycholate, 0.01% Nonidet P40, 5 mM K_4_Fe(CN)_6_, 5 mM K_3_Fe(CN)_6_, 2 mM MgCl_2_, 0.8 mg/ml X‐gal (stock solution: 40 mg/ml in dimethylformamide) and incubated overnight at 37°C. The tissue was next rinsed in PBS, cleared for 30 min in 50% v/v glycerol in PBS, sequentially mounted on slides.

### 2.6 Reverse transcriptase‐polymerase chain reaction (RT‐PCR)

*Calb1*, *Calb2*, and *Six3* cDNA fragments were obtained by reverse transcription (RT). RNA was individually extracted with Trizol reagent (Invitrogen, Carlsbad, CA, Cat. 10296‐028) from freshly dissected brains of *Mus musculus*. The RNA was treated with DNase I (Invitrogen, Cat. 18068‐015) for 15 min at room temperature (RT), and the enzyme was then inactivated at 65°C. Afterwards, RNA samples were converted to single‐stranded cDNA with Superscript III reverse transcriptase (Invitrogen, Cat. 18080‐044) and oligo‐dT‐anchored primers. The resulting first‐strand cDNA (0.5 μl of the reverse transcription reaction) was used as a template for the PCR reaction, which was performed in presence of *Taq* polymerase (Promega, Cat. M8305) and the following gene‐specific primers for *Calb1*, *Calb2*, and *Six3* mRNAs. Used primers were:

mCalb1F: 5′GCTGCTCTTTCGATGCCAGC 3′

mCalb1R: 5′GTGTCATCTGGCTACCTTCC 3′

mCalb2F: 5′GATGCTGACGGAAATGGGT 3′

mCalb2R: 5′ACCCTACCAGCCACCCTCTC 3′

mSix3F: 5′CTGGAGGAGACGGGCGACAT 3′

mSix3F: 5′GCTGGGGTTGGGGTAGGGAT 3′

The PCR conditions used were an initial denaturation step at 94°C for 5 min, then 35 cycles (30 s at 94°C, plus 1 min at *T*_m_ temperature (58°C), and 1 min at 72°C), followed by 20 min at 72°C. The PCR products were cloned into the pGEM‐T Easy Vector (Promega, Cat. A1360), and sequenced (SAI, University of Murcia).

### 2.7 In situ hybridization (ISH)

The tissues were processed for in situ hybridization with digoxigenin‐UTP‐labeled antisense riboprobes. Sense and antisense digoxigenin‐labeled riboprobes for mouse *Calb1*, *Calb2*, *Enc1*, *Nkx2.2*, and *Six3* were synthesized following the manufacter's recommendations (Roche Diagnostics S.L., Applied Science, Barcelona, Spain), and applying specific polymerases (Fermentas, Madrid, Spain). Probe sequence information is provided in Table 2. In situ hybridization (ISH) was performed basically as described by Ferran, Ayad, et al. (2015). After hybridization, all sections were washed and incubated in a solution containing alkaline phosphatase‐coupled anti‐digoxigenin antibody (diluted 1:3.500; Roche Diagnostics). Nitroblue tetrazolium/5‐bromo‐4‐chloro‐3‐indolyl phosphate (NBT/BCIP; Roche) solution was then used as chromogenic substrate for the final alkaline phosphatase reaction (Boehringer, Mannheim, Germany). No specific signal was obtained with sense probes (data not shown).

**TABLE 2:** List of gene probes used for in situ hybridization and their principal characteristics

Gene symbol	NCBI accession number	Size (bp)	Position	Publication/laboratory
*Calb1*	NM_009788.4	1,149	400–1,548	Present results
*Calb2*	NM_007586.2	895	160–1,054	Present results
*Enc1*	NM_007930.4	1,973	2,779–4,752	M.C. Hernández/present results
*Nxk2.2*	U31566.1	2,018	1–2018	J. Rubenstein/present results
*Six3*	NM_011381.4	408	704–1,111	Present results

*Dlx1*, *Dlx2*, *Dlx5*, *Dlx6*, *Isl1*, *Pax6*, *Ecel1* and *Sst* expression was analyzed from in situ hybridization images downloaded from the Allen Developing Mouse Brain Atlas (https://developingmouse.brain-map.org).

### 2.8 Immunohistochemistry (IHC)

For comparative purposes, the mappings sometimes included immunochemical detection of calbindin (CB), calretinin (CR), distalless homeobox protein (DLX), nitric oxide synthase (NOS), neuropeptide Y (NPY), parvalbumin (PV), paired box protein 6 (PAX6), T‐box, brain, 1 protein (TBR1), and tyrosine hydroxylase (TH).

Our immunohistochemical reaction protocol was described in detail elsewhere (Ferran, Ayad, et al., 2015). The primary antibodies used are described in Table 3. After washes, the sections were incubated with biotinylated goat anti‐rabbit or goat anti‐mouse (Vector Laboratories, CA; used at 1:200 dilution) followed by a streptavidin–peroxidase complex (Vectastain‐ABC kit; Vector Laboratories; 0.001% dilution), applied for 1 hr at room temperature. Peroxidase activity was developed with 0.03% 3,3′‐diaminobenzidine (Sigma; St Louis, MO), plus 0.003% hydrogen peroxidase. After immunohistochemical and hybridization labeling, the slides were washed several times in PBS, air‐dried and coverslipped with Cytoseal 60 (Thermo Scientific, Ref. 8310‐16) or Mowiol (Calbiochem, Bad Soden, Germany, Ref. 475904). We verified the specificity of the antibodies by performing parallel control experiments that omitted the primary antibody, checking that no residual immunostaining was detected (data not shown).

**TABLE 3:** Antibody list

Name	Immunogen	Manufacturer, host species, RRID, catalog number	Dilution
Calbindin (CB)	Recombinant rat calbindin D‐28k.	Swant Bellinzona, Switzerland, Cat# CB 38; RRID:AB-10000340; rabbit polyclonal; in immunoblots it recognizes a single band of approximately 27–28 kDa. It cross reacts with calbindin D‐28k from many other species, including human, monkey, rat, mouse chicken and fish	1:1,500
Calretinin (CR)	Recombinant human calretinin containing a 6‐his tag at the N‐terminal.	Swant Bellinzona, Switzerland, Cat# CR 7697 RRID:AB_2619710 rabbit polyclonal–calcium dependent reaction, code 7696, lot 25392; This antiserum does not cross‐react with calbindin D‐28k or other known calcium binding‐proteins, as determined by immunoblots and by its distribution in the brain. The antibody was evaluated for specificity and potency by (a) Biotin‐Avidin labeling of cryostate‐, vibratome‐, and paraffinsections of 4% paraformaldehyde fixed brains and (b) immunoenzymatic labeling of immunoblots	1:2,000
Pan‐distalless homeobox protein (DLX)	A 200 amino‐acid butterfly distalless peptide comprising the NH2‐terminal sequences expressed in pET23a with a histidine tag and purified on a nickel column, followed by two injections with the 61 amino acid homeodomain.	A kind gift from Grace Boekhoff‐Falk (previously G.Panganiban), University of Wisconsin‐Madison; Wisconsin; rabbit polyclonal. Antibodies are column affinity purified using the distal‐less protein. Immunohistochemistry to vertebrate tissues show expression patterns that are indistinguishable from the sum of the *Dlx1*,*2*,*5*,*6* RNA expression patterns (Stühmer, Anderson, Ekker, & Rubenstein, 2002; Stühmer, Puelles, et al., 2002).	1:400
Nitric oxide synthase (NOS)	A recombinant protein consisting of 195 amino acids from the N‐terminal of rat nNOS protein (the exact positions are a company secret, according to Invitrogen Technical Service)	ZYMED Laboratories (now Invitrogen Immunodetection), Carlsbad, CA; Z‐RNN (equals Z‐RNN3) and is now Cat#61‐7000, AB_2313734; rabbit polyclonal; This antibody reacts with the ~160 kDa nNOS protein and does not exhibit any cross‐reactivity with the related eNOS or iNOS proteins. During development reactivity was confirmed with *a* ~ 160 kD band on western blots of rat and mouse brain tissue lysates (20 μg).	1:3,000
Neuropeptide Y (NPY)	Synthetic porcine neuropeptide Y conjugated to bovine serum albumin: YPSKPDNPGEDAPAEDLARYYSALRHYINLITRQRY‐NH2.	Incstar (now Immunostar Inc) Hudson, WI; Cat t# 22940; RRID:AB_572253; rabbit polyclonal; The antibody was raised to NPY coupled to BSA with glutaraldehyde. The glutaraldehyde couples the NPY to the BSA using free amine groups. This strategy was intended to target the amino terminus of NPY, though other conformations were likely present since glutaraldehyde binds any free amines it can find, including those on amino acids such as lysine	1:2,000
Parvalbumin (PV)	Purified frog muscle parvalbumin	Sigma–Aldrich Cat# P3088 RRID:AB_477329; monoclonal (clone PARV‐19) Isotype Mouse; IgG1, lot 056H4830: Immunoblot: Single band 12 kDa; Monoclonal Anti‐Parvalbumin (mouse IgG1 isotype) is derived from the PARV‐19 hybridoma produced by the fusion of mouse myeloma cells and splenocytes from an immunized mouse. Purified frog muscle parvalbumin was used as the immunogen. The isotype is determined using Sigma ImmunoTypeTM Kit (Product Code ISO‐1) and by a double diffusion immunoassay using Mouse Monoclonal Antibody Isotyping Reagents (Product Code ISO‐2).	1:2,000
Paired box protein 6 (PAX6)	Peptide sequence: QVPGSEPDMSQYWPRLQ of the C‐terminus of the mouse PAX6 protein. The sequence is highly conserved among Pax‐6 of various species.	Covance, polyclonal rabbit anti‐Pax6, PBR‐278P100, RRID: AB_291612. Characterization: None provided by the supplier	1:300
T‐box, brain, 1 protein (TBR1)	Amino acid residues 614–624 of mouse Tbr1: DSSDSGIYEQAKRRRISPADT	A kind gift from Yi‐Ping Hsueh, Institute of Molecular Biology, Academia Sinica, Taipei, Taiwan. Characterization: western blot/ICH. Loss of immunoreactivity in *Tbr1* mutant mouse. Staining pattern precisely matches expression of *Tbr1* in the mouse telencephalon (Hevner et al., 2001)	1:100
Tyrosine hydroxylase (TH)	SDS‐denatured, native rat TH purified from pheochromocytoma	Novus Cat# NB300‐109 RRID:AB_10077691; Rabbit polyclonal; Immunoblot: single band of 60 kDa; western blot: 10 μg rat caudate lysate or PC12 cell lysate elicits single band 60 kDa; This tyrosine hydroxylase antibody recognizes all mammalian and at least some nonmammalian forms of the enzyme in western blots and in IHC/IF.	1:1,000

### 2.9 Interpretation rationale for double *Dlx5*/*6‐LacZ* and ulterior Pan‐Distalless immunoreaction (any DLX protein)

We studied a sagittally cut *Dlx5*/*6*‐*LacZ* P0 brain which was secondarily immunoreacted with the Pan‐Distalless antibody (see Table 3). It is usually thought on the basis of published data on the striatum that during development *Dlx*‐expressing cells activate sequentially the four different neural paralog *Dlx* forms, that is, starting with *Dlx1*/*2* at postmitotic stages and proceeding on to express *Dlx5*/*6* as differentiation advances, frequently with accompanying downregulation of *Dlx1*/*2* (Eisenstat et al., 1999). Presence of the corresponding mRNAs and proteins would be expected at the appropriate stages, so that specific ISH reaction and some immunoreaction with the pan‐distalless antibody should be a general feature where a *Dlx* gene is being expressed. On the other hand, in our double‐labeled material we found well delimited only blue (*LacZ*‐positive), only brown (DLX immunoreactive) and mixed blue‐brown areas. In the following lines, we explain the interpretive rationale we finally followed with this complex material, which bespeaks of a subtly differential nature of the corresponding progenitor subdomains, consistently with conclusions derived from other molecular markers studied. In this rationale, we considered the possibility that the *LacZ* reaction may have partially or totally quenched subsequent immunoreaction of the DLX proteins (particularly in cases where their cytoplasmic concentration was not high). We examined as well public *Dlx1*/*2*/*5*/*6* gene in situ data available at the Allen Developing Mouse Brain Atlas which show the prethalamic topography of some of these transcripts at perinatal (E18.5‐P4) stages. We arrived at the following conclusions.

**FIGURE 2:**
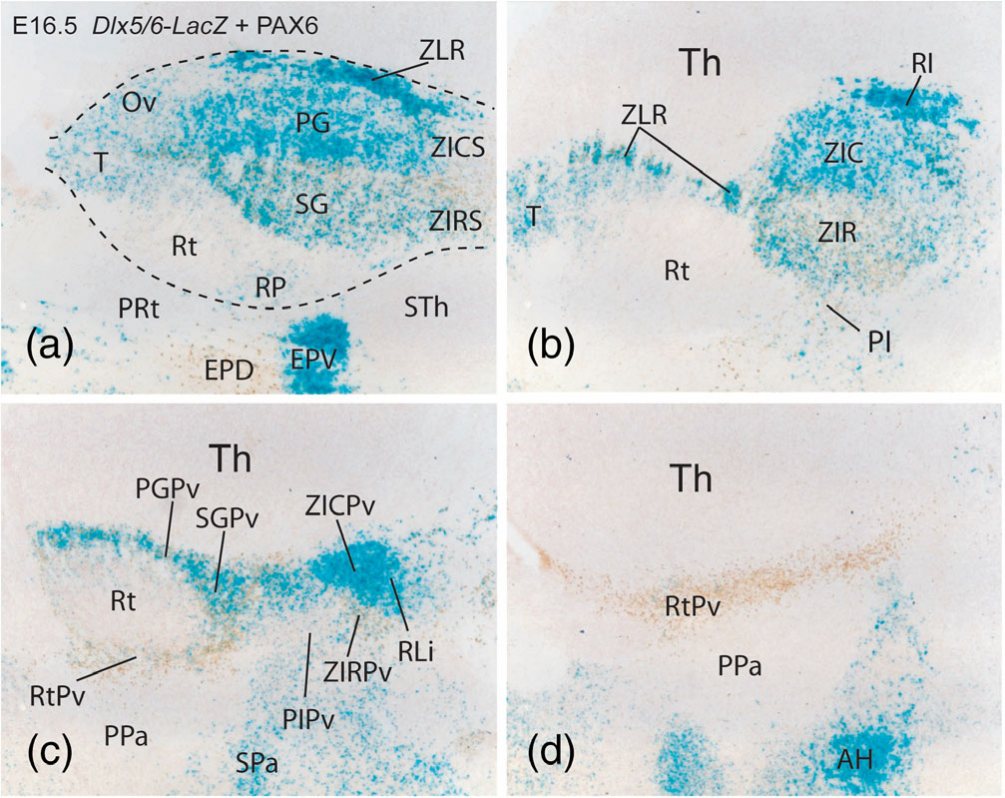
Lateromedial series of sagittal sections though the prethalamus of an E16 mouse embryo carrying the *Dlx5/6‐LacZ* construct and immunoreacted with anti‐PAX6 antibody. The tagged rostrocaudal subdivisions are evident superficially (a,b). Rostral is oriented to the bottom and dorsal to the left. Weakly immuno‐labelled PAX6 cells are restricted to the Dlx‐positive SG/ZIR complexes, where the *Dlx5/6‐LacZ* reaction is weaker than at the PG/ZIC complex (SG, PG, ZIR, ZIC; a,b), as well as to the periventricular stratum deep to the Rt (RtPv; c,d). *Dlx‐LacZ* reaction is very strong at the ZLR/RI sites, rather weak at the dorsal triangular and oval nuclei, and practically absent at the Rt (ZLR, RI, T,Ov, Rt in a,b). Within the neighboring peduncular hypothalamus the dorsal entopeduncular nucleus (EPD) is PAX6‐positive and *LacZ*‐negative, whereas the ventral entopeduncular nucleus (EPV) strongly expresses *Dlx‐LacZ* signal (a). [Color figure can be viewed at wileyonlinelibrary.com]

We identified purely blue‐labeled cells as elements that at some earlier stage were *Dlx5*/*6*‐positive (since they show the *Dlx5*/*6*‐*LacZ* reaction product), but secondarily significantly downregulated the amounts of both DLX5/6 and DLX1/2 protein present, so that their immunoreaction at stages E18.5‐P4 was below detection level, any protein remnants possibly having been quenched by the *LacZ* histochemical procedure. Indeed, we confirmed at the Allen Developing Mouse Brain Atlas that these “blue only” PTh sites display at E18.5 and P4 low *Dlx1*/*2* in situ reaction and no *Dlx6* ISH signal (therefore, blue labeling = persistent *LacZ* reaction possibly maintained by low levels of *Dlx1*/*2*, with low DLX1/2 protein levels [quenched signal?] and no significant DLX6 protein levels, insufficient for immunoreaction).

On the other hand, selectively brown‐labeled cells theoretically must represent cells having downregulated any *Dlx5*/*6* signal already at rather early stages (as revealed by overall presence of early embryonic *Dlx5*/*6*‐*LacZ* signal at E13.5, but lack of it at specific PTh sites at P0), while still containing sufficient DLX1 protein at P0 to be detected by the pan‐distalless antibody (as predicted, these loci show significant *Dlx1*/*2* ISH signal at E18.5 and P4; Allen Developing Mouse Brain Atlas) (accordingly, brown labeling = implies only persistent *Dlx1*/*2* and DLX1/2 expression; therefore no postnatal *Dlx5*/*6* or *Dlx5*/*6*‐*LacZ* signal).

Finally, we interpreted the double‐labeled cells as revealing sites with persistent detectable levels of DLX5/6 protein at P0 (apparently mainly DLX6), sufficient to elicit visible immunoreaction in spite of quenching effects of the local successful *Dlx5*/*6*‐*LacZ* reaction (we see at these sites marked *Dlx6* ISH reaction, but little *Dlx5* or *Dlx1*/*2* ISH signal at E18.5 and P4; Allen Developing Mouse Brain Atlas) (in conclusion, blue + brown labeling = mainly DLX6 and *Dlx6*; low *Dlx1*/*2*/*5* or corresponding proteins).

### 2.10 Imaging

Sections were photographed with a digital Zeiss AxioCam camera through a Zeiss Axiophot microscope. Alternatively, whole‐slide digital images were acquired with a ScanScope CS digital slide scanner at high resolution (Aperio Technologies, Inc.; Vista, CA). After scanning, the visualization and capture of images of adjacent labeled sections were carried out by using the Aperio software ImageScope. Contrast and focus were adjusted by applying Adobe Photoshop CS3 software (Adobe Systems, Inc., San Jose, CA).

## 3 RESULTS

For simplicity, we will refer to “*Dlx5*/*6*‐promoter‐driven‐*LacZ* expression” as “*LacZ* signal” or “*Dlx*‐*LacZ* signal.” Our description focuses on the prethalamus, with minimal attention to neighboring areas such as the prethalamic tegmentum, the interthalamic zona limitans, the alar and basal hypothalamus, or the substantia nigra. After some introductory embryonic data at stages E13.5 and E15.5 (Figures 1,2), we attend successively to stages P0, P4, P15, P40, and P140, offering images of sagittal and horizontal sections (Figures 3–21), with occasional inclusion of some other embryonic images, for specific reasons (E16.5, E18.5). We will emphasize in the text the major changes observed at each time point.

### 3.1 Embryonic pattern

The general distribution of *Dlx* signal in the prethalamus can be optimally observed in embryonic material (Bulfone et al., 1993; Liu et al., 1997; Puelles & Rubenstein, 1993; Simeone et al., 1994). In our analysis of E11.5, E12.5, E13.5, E15.5, and E16.5 embryos the *Dlx*‐*LacZ* signal (as well as GAD67 immunoreaction) extends throughout the sum of central prethalamus (PThC), subcentral prethalamus (PThSC) and prethalamic zona limitans‐related areas (ZLR/RLi), whereas the hyperdorsal prethalamic eminence is distinctly *Dlx*‐negative (PThC, PThSC, PThE, ZLR/RLi; Figures 1a–c,e and 21). PThE shows a differential molecular profile, displaying, for example, PAX6 and *Gdf10* expression at its ventricular zone (Figure 1a,b; Puelles, Martinez‐de‐la‐Torre, Bardet, et al., 2012; Shimogori et al., 2010), as well as TBR1 (Figure 1c), *Tbr2*, *Lhx5*/*9*, and calretinin (*Calb2*) signal (Abbott & Jacobowitz, 1999; Abellán et al., 2010; Shimogori et al., 2010) at its mantle layer (Figure 1e). Interestingly, some of the genoarchitectural properties of the PThE ventricular and mantle zones (including lack of *Dlx*‐*LacZ* signal) also continuously extend rostrally into the paraventricular hypothalamic area and overlying telencephalic pallium (Pa; Figure 1c). However, the paraventricular hypothalamic area expresses differentially other markers, such as *Otp* and *Sim1*, and lacks significant *Lhx9* and *Calb2* signals (Morales‐Delgado et al., 2014; Morales‐Delgado, Merchan, Bardet, Ferran, & Díaz, 2011; Puelles, Martinez‐de‐la‐Torre, Bardet, et al., 2012; Shimogori et al., 2010).

Caudally, there is a very sharp transverse embryonic limit of the prethalamic *Dlx*‐*LacZ* domain at the interthalamic p2/p3 boundary, known classically as the zona limitans interthalamica (ZL; Figures 1a–c,e and 21a–c; Gilbert, 1935; Kuhlenbeck, 1973; Puelles, 2018; Rendahl, 1924); this landmark has been widely documented in modern molecular developmental literature, including its relationship with the expression of *Dlx* family genes (see combined *Gbx2*/*Pitx2*/*Dlx2* image in S Martinez, Puelles, Puelles, & Echevarria, 2012; fig. 1.6A.B).

This peculiar locus is now recognized as the mid‐diencephalic secondary organizer (review in Puelles & Martinez, 2013). The equally transverse prethalamo‐hypothalamic boundary lies just caudal to the peduncular hypothalamus, the transverse hypothalamic sector which is traversed dorsoventrally by the cerebral peduncle (sum of the medial and lateral forebrain bundles, continuous dorsally with the telencephalic internal capsule; Puelles, Martinez‐de‐la‐Torre, Bardet, et al., 2012; Puelles & Rubenstein, 2015). The hypothalamo‐diencephalic limit also coincides at periventricular level with the sharp caudal boundary of the hypothalamic paraventricular nucleus, as visualized with the *Otp* and *Sim1* gene markers expressed selectively there (Morales‐Delgado et al., 2011; Morales‐Delgado et al., 2014; Puelles, Martinez‐de‐la‐Torre, Bardet, et al., 2012; Shimogori et al., 2010). A peculiarity observed at E13.5 and E16.5 embryonic stages, in contrast to the E11.5 pattern, is that the primarily widespread *Dlx5*/*6*‐*LacZ* signal observed throughout the PThC and PThSC mantle becomes increasingly downregulated in some prethalamic derivatives, notably at the reticular nucleus, irrespective that some *Dlx* paralogue forms remain detectable there by immunoreaction (Figures 1a,c and 21a–c).

### 3.2 Regional and areal constitution of the prethalamus

We will present here our conclusions about the overall structural model of the prethalamus, to facilitate subsequent detailed description of marker distributions (compare Figure 1d,e).

#### 3.2.1 Dorsoventral regions

Note we use the relatively recent “prethalamus” term (PTh) as representing the alar plate domain of the diencephalic prosomere 3 (the rostralmost diencephalic segmental unit; see Introduction). This name was originally proposed in substitution of the obsolete columnar term “ventral thalamus” (Puelles & Rubenstein, 2003); it refers explicitly to the topologic position occupied by this rostral diencephalic domain in the prosomeric model (Figure 1d). Our overall structural conclusion is that the PTh region is tetrapartite both dorsoventrally and rostrocaudally (Figure 1d,f–h). Most dorsally, it includes the *Dlx*‐negative *prethalamic eminence* (PThE; Puelles and Rubenstein (2003); Hayes et al. (2003)). The PThE is understandable as a hyperdorsal alar plate derivative, which ends in contact with the local chorioidal roof plate (PThE, ch; Figures 1d,e and 21f; Puelles, 2013; fig. 10.4e). The stria medullaris tract runs longitudinally through the PThE, continuing into the similarly hyperdorsal thalamic habenula subdomain (sm, Hb; Figure 1b,d,e). Under the PThE (ventrally) there appears a larger PTh territory presently named by us the *central prethalamus*, which is followed more ventrally by a relatively smaller *subcentral prethalamus* territory (PThC, PThSC; Figures 1a–e and 21g,h; Puelles, 2013; fig. 10.4e). The PThC contains the major prethalamic derivatives relating to the bidirectional thalamo‐reticulo‐cortical connections and the longitudinal optic tract. It includes a deep *periventricular* stratum (often misidentified as a part of zona incerta), the reticular nucleus, which is the main rostral *intermediate* mantle derivative, with correlative middle and caudal intermediate PThC elements, and the *superficial* visual retropeduncular, subgeniculate and pregeniculate nuclei (CPv, Rt, RP, SG, PG; Figure 1a–e; Puelles, 2013; fig. 10.4e). The PThSC instead contains the *rostral* and *caudal* parts of the *zona incerta* complex (note obsolete columnar usage gives them as “ventral” and “dorsal” parts, respectively), and includes rostrally the newly recognized *preincertal nucleus* (PI, ZIR, ZIC; Figures 1a–e and 21g,h; Puelles, 2013; fig. 10.4e). Underneath the PThSC, there appears a uniform, strongly *Dlx5*/*6*‐*LacZ* positive band which forms the ventral rim or *limen* of the alar plate, which borders on the underlying basal plate or prethalamic tegmentum. We named it the *rostral liminar band* (RLi), since there is also a caudal counterpart under the thalamus and pretectum (with partially different molecular profile). In its longitudinal prethalamic course, the RLi band reportedly lies dorsoventrally halfway across the alar‐basal boundary as defined by basal plate *Shh* expression (RLi; p3Tg; figs. d,e; Puelles, Martinez‐de‐la‐Torre, Bardet, et al., 2012). This overlap is due to the early activation of *Nkx2.2*/*2.9*, *Ptc [patched]*, and other SHH responsive genes, where SHH secreted and diffused from basal plate cells reaches highest concentration levels; this apparently occurs across the alar‐basal boundary. The RLi is continuous rostrally with a similar liminar formation in the hypothalamus (Figure 1a–e). In addition, the RLi is continuous at its caudal end with the molecularly analogous ZLR (*rostral shell* of the *zona limitans*) a thin nearly transverse *Nkx2.2*‐ and *Dlx*‐positive domain which limits caudally the PThE, PThC and PThSC prethalamic regions relative to the zona limitans interthalamic boundary (ZLR; Figure 1a–h; review in Puelles & Martinez, 2013). The zona limitans (ZL) is equivalent to the embryonic mid‐diencephalic secondary organizer, which is located at the transverse p3/p2 boundary, normally seen only across its alar plate subdomain, though it also extends cryptically into the basal plate (ZL; Figures 1a–e and 21c; Andreu‐Cervera et al., 2019). It possesses a *sonic hedgehog* (*Shh*)‐expressing core domain (ZLCo), and is flanked by thin *rostral* prethalamic and *caudal* thalamic *Shh*‐negative and *Nkx2.2*‐positive shell domains (ZLCo, ZLR, ZLC; Figure 1d,e). Only the rostral shell domain (ZLR) is prethalamic, and it differentially displays a strong *Dlx5*/6‐*LacZ* reaction, jointly with selective *Dbx1* expression. The p3 basal plate territory, or *prethalamic tegmentum* (p3Tg), lies ventral to the RLi, and abuts the forebrain floor at the rostralmost part of the cephalic flexure, just behind the hypothalamic retromamillary area (in hp1), and in front of the thalamic (p2) prerubral tegmentum (p3Tg, RM, p2Tg; Figure 1a–e). The p3Tg also displays some Dlx5/6‐LacZ‐positive neurons (Figures 1b,c and 21c).

#### 3.2.2 Anteroposterior domains

As we illustrated schematically in Figure 1d,e,g,h our results support a shared anteroposterior (AP) tripartition of the PThC and PThSC subregions, while such subdivision is absent at the PThE and RLi domains. The ZLR represents by itself an independent fourth AP prethalamic domain. These AP details will be elaborated in the following sections.

#### 3.2.3 Radial subdivisions

Each of the progenitor domains identified as anteroposterior divisions of PThC and PThSC show subtle adult structural differences along the radial dimension (i.e., they show stratification along the ventriculopial axis, Figure 1g,h). These allow the overall distinction of periventricular, intermediate and superficial strata or derived nuclei (to indicate such radial location we added Pv, I or S to the areal name abbreviations where it was necessary; note that in some cases the better known formations lie in the intermediate stratum—this occurs with the reticular nucleus and the zona incerta nuclei, while in other cases they are superficial formations—for example, pregeniculate, oval and subgeniculate nuclei; these principal elements were generally named without suffix, for clarity and simplicity).

### 3.3 *LacZ*‐positive prethalamic cell populations at P0 and P4

**FIGURE 3:**
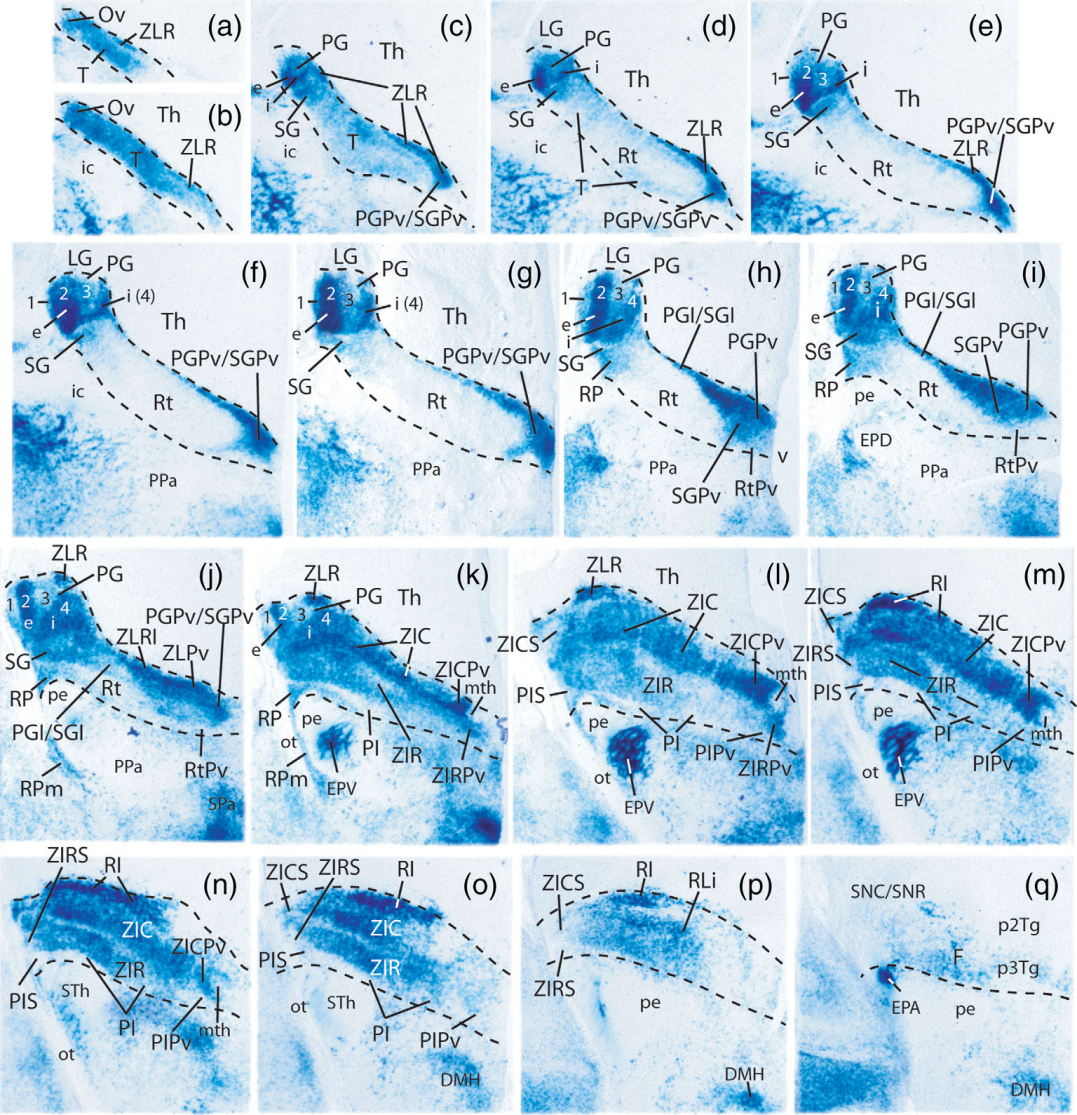
Dorsoventral series of horizontal sections through the prethalamus of a *Dlx5*/*6*‐*LacZ* P0 mouse brain illustrating differences in the signal in radial (ventriculo‐pial), dorsoventral and rostrocaudal subdivisions. Nuclear primordia are tagged according to the abbreviations list. The midline lies to the right, caudal is oriented to the top of the panels. *Dlx5*/*6* signal is restricted to the prethalamus, but shows evident downregulation at the intermediate reticular nucleus (Rt). Black dash lines indicate the caudal thalamo‐prethalamic and rostral hypothalamo‐prethalamic boundaries (note here the rostral relationship with internal capsule and cerebral peduncle; ic, pe). Note the thalamus (Th) is free of *Dlx5*/*6‐LacZ*‐labeling whereas the hypothalamic ventral entopeduncular, accessory entopeduncular and dorsomedial nuclei (EPV, EPA, DMH) display *Dlx5*/*6‐LacZ* signal [Color figure can be viewed at wileyonlinelibrary.com]

**FIGURE 4:**
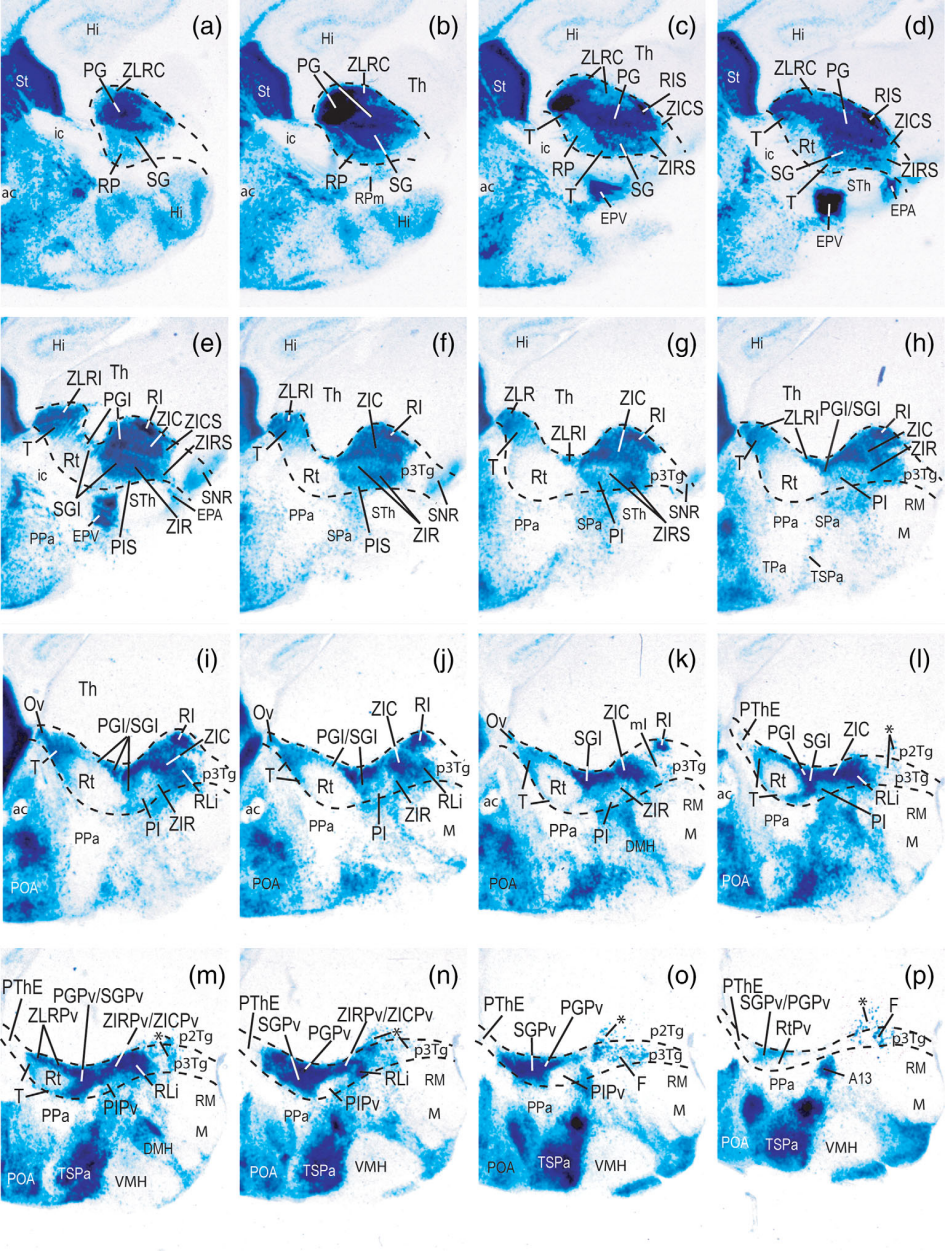
Lateromedial series (a–p) of sagittal sections showing *Dlx5*/*6‐LacZ* reaction and dorsoventral and anteroposterior subdivisions in a P0 mouse brain prethalamus. Black dash lines indicate the caudal thalamo‐prethalamic and rostral hypothalamo‐prethalamic boundaries. Caudal is oriented to the top right, dorsal to the top left (part of telencephalic subpallium is visible for reference). The asterisks in l–p indicate dispersed *Dlx* cells in the basal p3 and p2 tegmentum (p3Tg; p2Tg). Strong *Dlx5*/*6‐LacZ* signal is also present in hypothalamic territories, excepting at the alar paraventricular subdomain (Pa), retrotuberal subthalamic nucleus and retromamillary area (STh, RM), or the tuberal ventromedial nucleus and the mamillary area (VMH, M). Preoptic and striatal areas (POA, St) likewise have *Dlx5*/*6*‐*LacZ* signal [Color figure can be viewed at wileyonlinelibrary.com]

**FIGURE 5:**
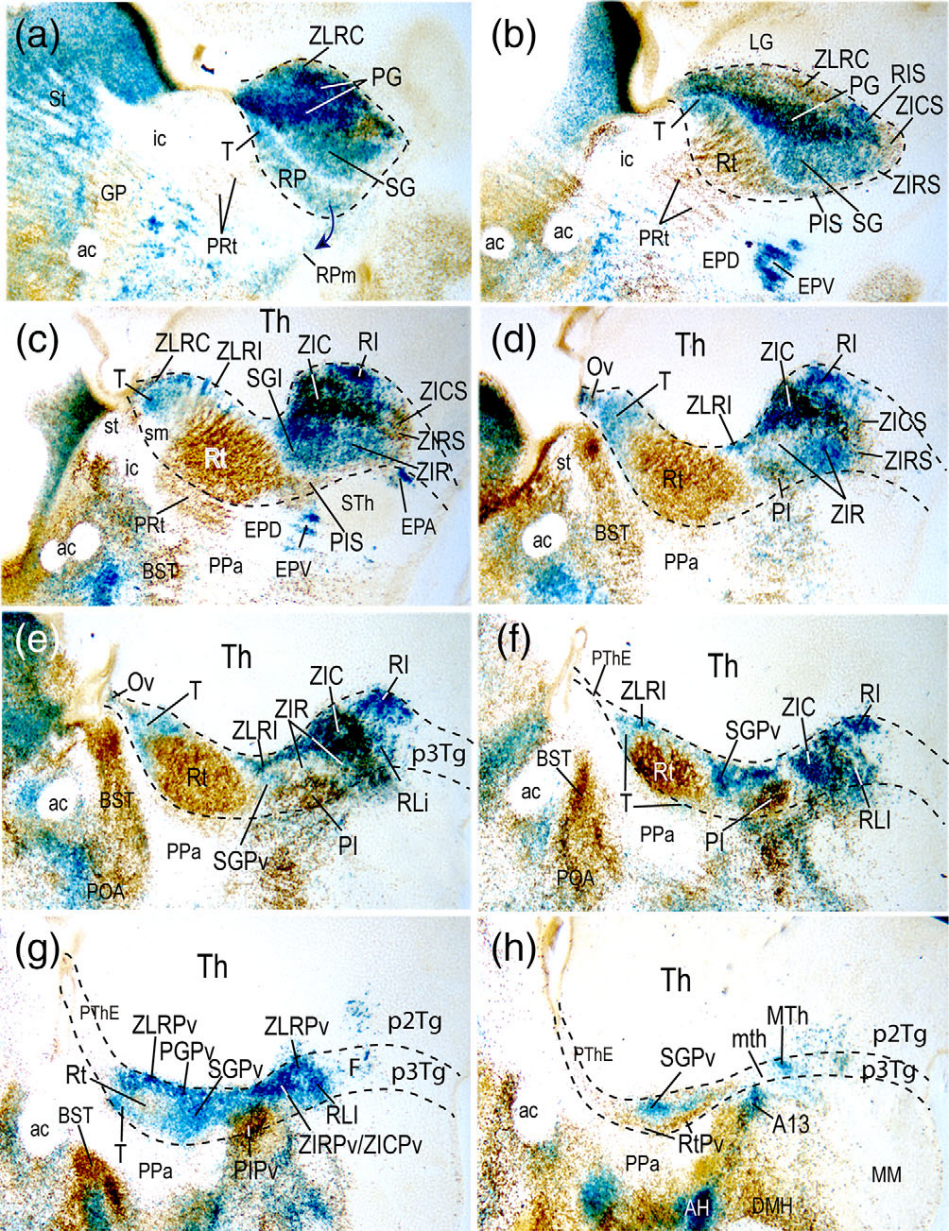
Lateromedial series (a–h) of sagittal sections through the prethalamus of a P0 mouse brain carrying the *Dlx5*/*6*‐*LacZ* construct, which were counterstained with pan‐distalless antibody (DLX; this antibody recognizes all DLX forms). Black dash lines indicate the prethalamo‐thalamic (intrathalamic) and hypothalamo‐prethalamic boundaries. Caudal is oriented to the top right, dorsal to the top left. Blue cells represent exclusive *Dlx5*/*6*‐*LacZ* signal, brown cells indicate exclusive DLX‐immunostaining, and double‐labeled cells show both reactions; see our interpretation criteria for this unusual labeling in Section 2. Combined labeling reveals rostrocaudal, dorsoventral and radial prethalamic components nondistinguishable with single *Dlx5*/*6*‐*LacZ* labeling (compare with Figure 4). Remarkably, brown DLX‐immunoreactive cells are restricted to locations devoid of *LacZ* signal, mainly the reticular nucleus, Rt (b–g), its periventricular stratum, RtPv (H) and the entire preincertal complex (PIS, PI, PIPv in (b–g)). Superficial and dorsal components of the reticular region, that is, the retropeduncular and triangular nuclei (RP, T), have blue‐labeled cells, in contrast with the remaining brown‐labeled Rt/RtPv region. The arrow in (a) points to apparent partial retropeduncular cell migration (RPm) into the peduncular hypothalamus. Note also differences of labeling between the superficial subgeniculate and pregeniculate nuclei, blue and double‐labeled, respectively, at the central prethalamus (SG, PG, in (a,b)). Similar labeling differences are observable between the rostral and caudal zona incerta components at the subcentral prethalamus (ZIR, ZIC in (c–e)). Note in b–d selective DLX immunoreaction characterizing the superficial subcentral components (PIS, ZIRS, ZICS), as well as the superficial cap of the ZLR (ZLRC) [Color figure can be viewed at wileyonlinelibrary.com]

**FIGURE 6:**
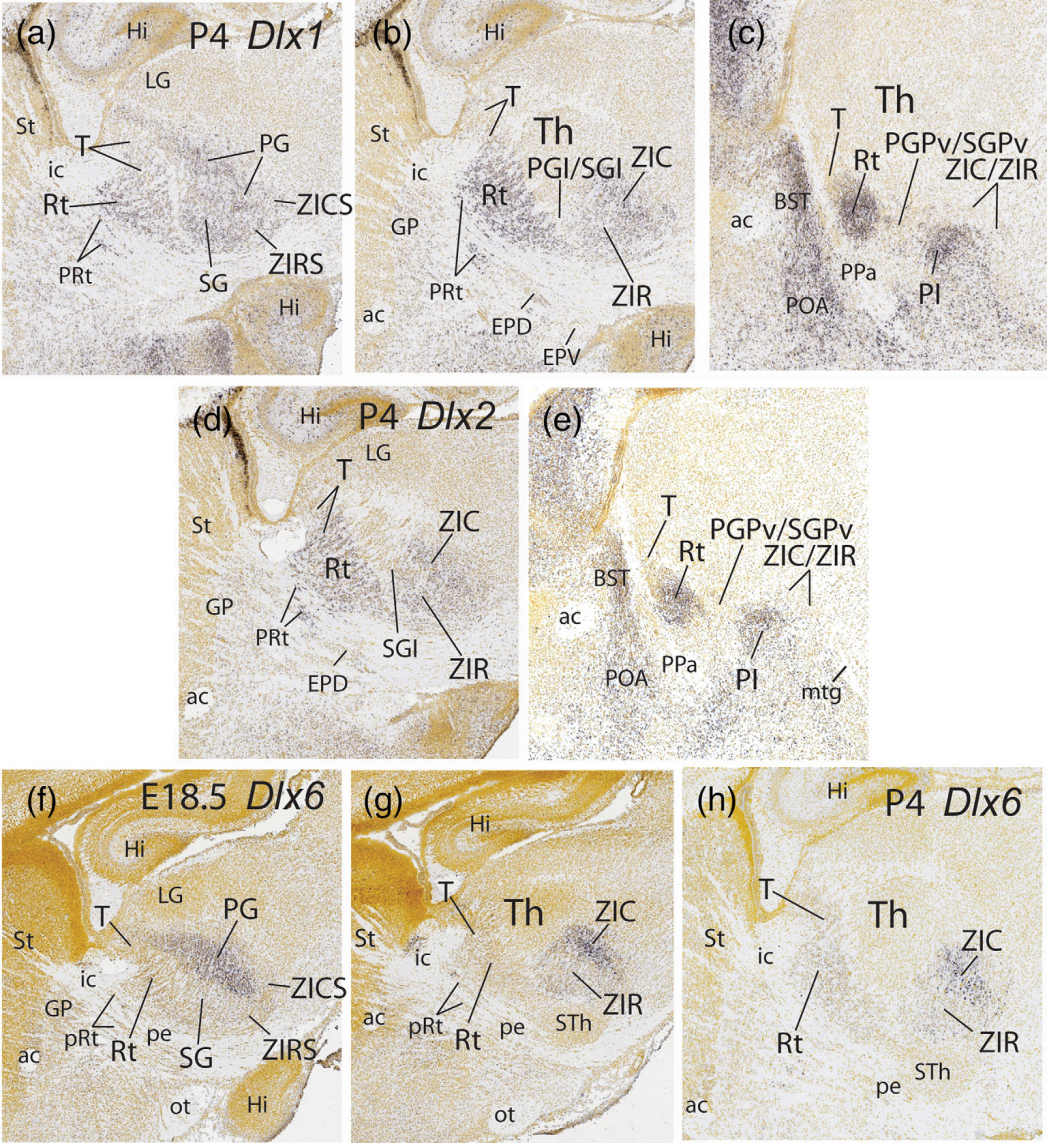
Expression of *Dlx1*, *Dlx2* and *Dlx6* in the prethalamus at perinatal stages. All images were downloaded from the Allen Developing Mouse Brain Atlas. Caudal is oriented to the top right and dorsal to the top left. (a‐c) Lateromedial sagittal sections of a P4 mouse brain HIS reacted for Dlx1. Strong labelling is mainly found at the rostral part of the central prethalamus (Rt) and subcentral prethalamus (PI), although Dlx1 is also present in SG, PG and ZIC. (d,e) Two sagittal sections of a brain at P4, ordered from lateral to medial, which show that *Dlx2* expression in the prethalamus is rather similar to the relatively stronger *Dlx1* labelling. (f‐h) Sagittal sections showing *Dlx6* ISH expression in E18.5 (f,g) and P4 (h) mouse brains. Section f is lateral to g. Section h represents a level equivalent to g. *Dlx6* labelling is mainly restricted to the caudal part of the central prethalamus (PG) and subcentral prethalamus (ZIC) [Color figure can be viewed at wileyonlinelibrary.com]

**FIGURE 7:**
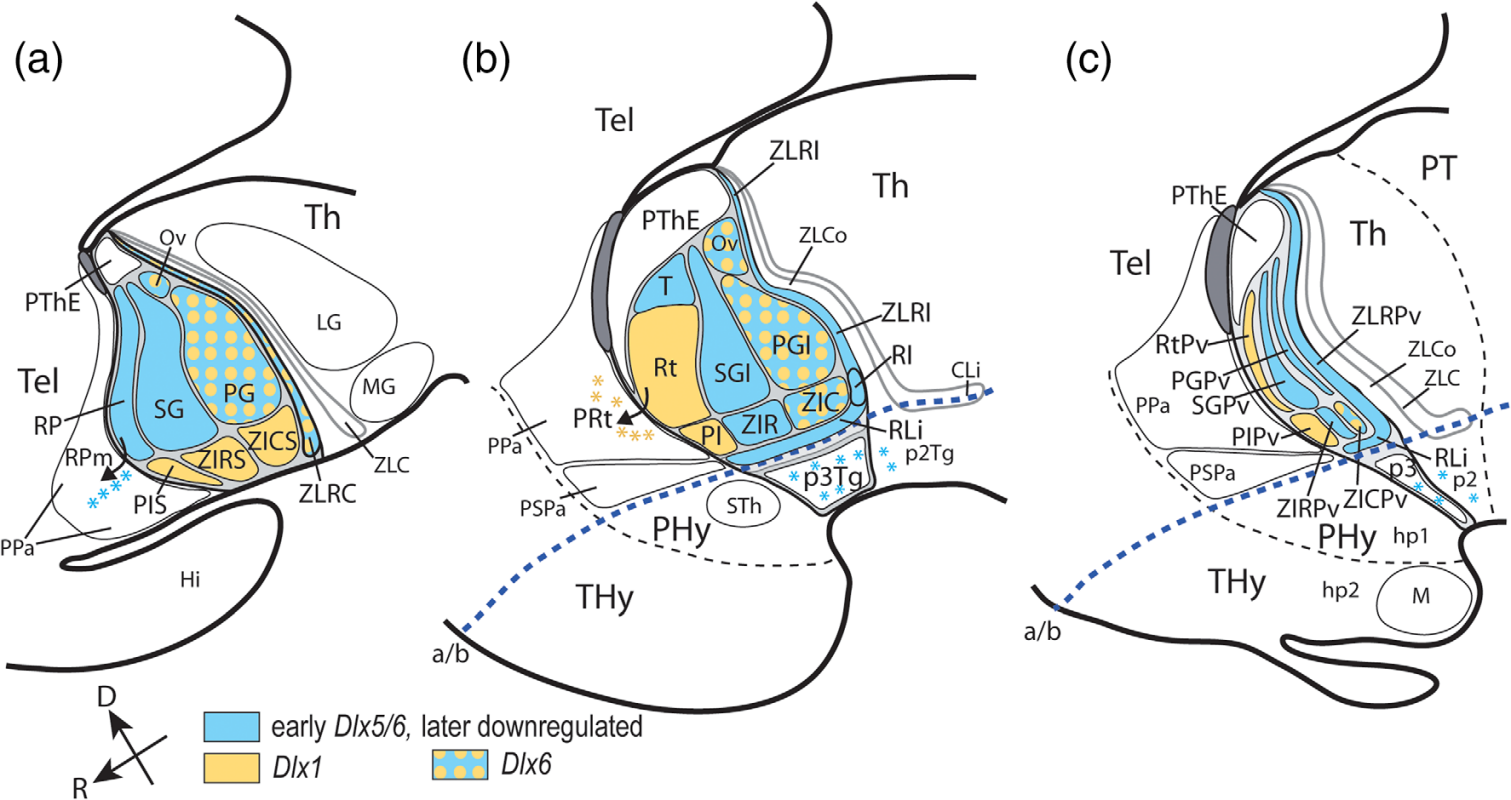
Three lateromedially ordered color‐coded schemata based on preceding Fig.5, which summarize Dlx5/6‐LacZ labelling combined with the pan‐distalless antibody (DLX) immunoreaction at superficial (a), intermediate (b) and periventricular (C) levels, interpreted in the context of correlative ISH data on *Dlx* forms found at the Allen Developing Mouse Brain Atlas (see Figure 6; resulting color‐code at bottom; following the rationale explained in Material and Methods, the conclusion is that only *Dlx1/2* and *Dlx6* RNA signals persist significantly in the prethalamus at P0, each with characteristic topography, irrespective of the conservation or not of the *Dlx5/6‐LacZ* reaction product). The prethalamus appears enclosed by black linear boundaries. Black dash lines indicate the thalamo/pretectal (Th/PT) and intrahypothalamic (THy/PHy) borders. The longitudinal alar/basal boundary (a/b) is represented as a blue dash line. The rostral (R) and dorsal (D) spatial directions are indicated in a. Blue asterisks and a black arrow in a mark apparently marginally migrated retropeduncular nucleus cells (RPm, RP) found superficial to the cerebral peduncle in the hypothalamus. Yellow asterisks accompanied by a black arrow in b indicate dispersed cells of the perireticular nucleus (PRt) present within the lateral hypothalamus (superficial to paraventricular nucleus within alar PHy), showing a brown immunoreaction pattern interpreted by us –rationale in Material and Methods‐ as consistent with postnatal‐persisting *Dlx1* RNA signal, similarly as occurs in the Rt nucleus (see Fig.6). Blue asterisks represented at p3Tg and p2Tg in b,c refer to *Dlx5/6‐LacZ*‐labelled elements presumably migrated into the basal plate. [Color figure can be viewed at wileyonlinelibrary.com]

**FIGURE 8:**
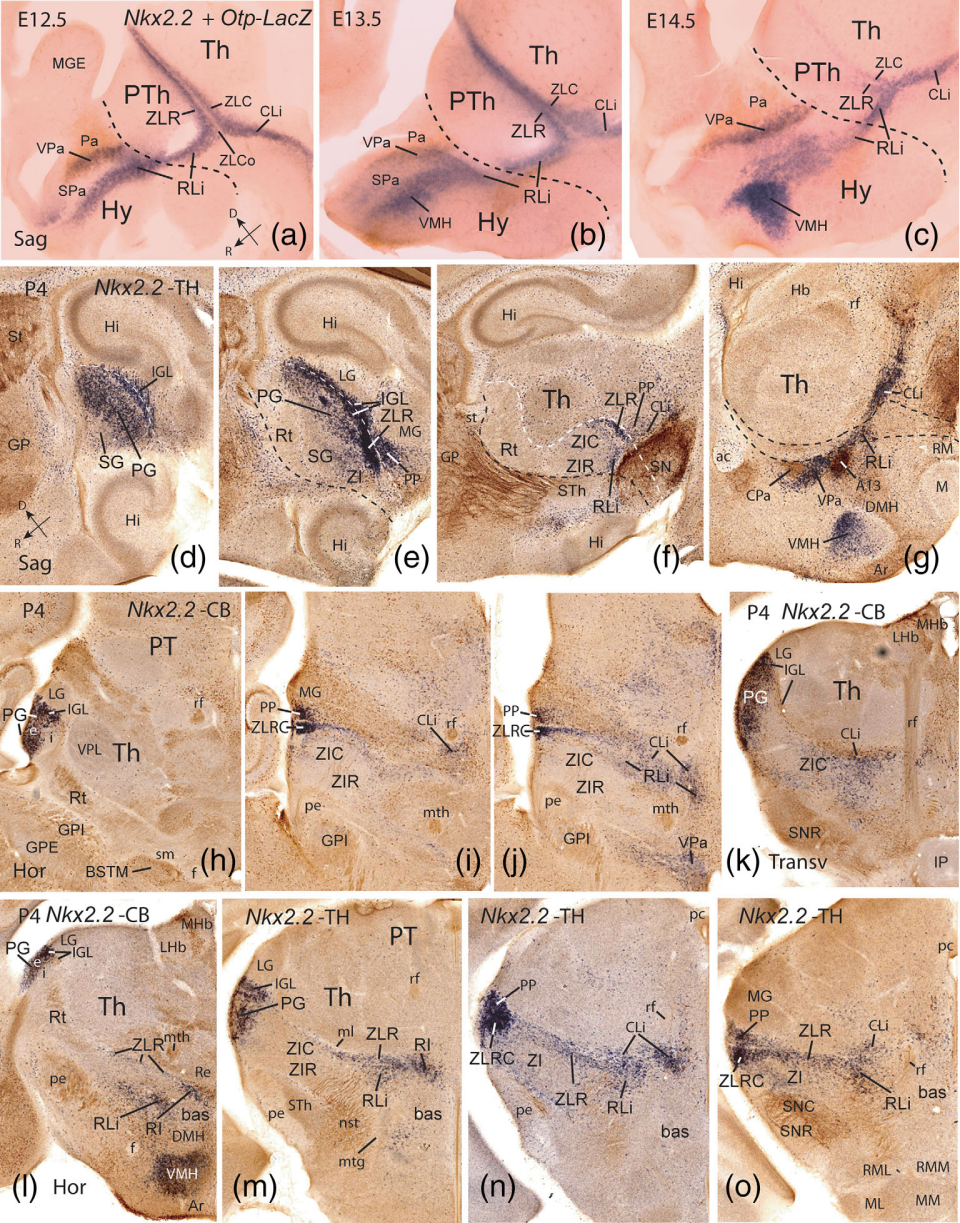
*Nkx2.2*‐expressing prethalamic derivatives at embryonic stages and P4. (a‐c) Sagittal sections through the early diencephalo‐hypothalamic mantle zone of embryos carrying an *Otp‐LacZ* construct at E12.5, E13.5 and E14.5. The sections were reacted for *Nkx2.2* ISH (blue), while *Otp‐LacZ* cells were visualized with an antibody against β‐galactosidase (brown). These Figures are modified from Figure 8.26 of Puelles, Martinez‐de‐la‐Torre, Bardet, et al. (2012), where the emphasis was placed on hypothalamic details (Hy; e.g., dorsal versus ventral tangential migration of hypothalamic *Nkx2.2*‐positive cells into VPa and VMH, respectively; compare with corresponding adult data in g; the hypothalamo‐prethalamic interprosomeric border is marked by a black dash line, typically passing just caudal to Pa, the paraventricular nucleus). Here we focus on *Nkx2.2* signal restricted to the transverse rostral and caudal shells of the zona limitans (ZLR, ZLC) at the prethalamus/thalamus border and the longitudinally continuing rostral and caudal liminar bands (RLi, CLi), which follow the alar/basal boundary. Note the Shh‐expressing ZLCo (Shh not shown) remains essentially *Nkx2.2*‐negative (a,b). As the dorsal tip of the ZL is approached, the ZLR and ZLC seem to fuse together (a,b). Dorsal (D) and rostral (R) spatial orientations are indicated in a. (d‐g) Lateromedial series of sagittal sections through the prethalamus of a postnatal P4 brain hybridized for *Nkx2.2* (blue) and immunostained for tyrosine hydroxylase (TH; brown). Note in (d,e) the presence dorsally, in front of the LG, of superficial *Nkx2.2*‐expressing cells in the intergeniculate leaflet (IGL), an apparently fused derivative of ZLR+ZLC, as well as in the pregeniculate nucleus (PG); the latter elements probably have migrated tangentially from the IGL, since they were not seen at early embryonic stages (a‐c). More ventrally, the IGL band bifurcates in an acute angle ventralwards; we identified the rostral branch as the ventral part of the ZLR, passing underneath the pregeniculate nucleus (PG) into the RLi (e‐g). In contrast, the caudal, slightly more diffuse IGL branch apparently represents a ventral part of ZLC in the form of its derivative, the conventional peripeduncular nucleus (PP), typically found in front of the MG. A black dash line illustrates the transverse rostral border of the prethalamus with the hypothalamus, while a white dash line marks the prethalamo‐thalamic border. (h‐j) Three conventional horizontal sections through the prethalamus at P4 (dorsoventral order), illustrating derivatives of the *Nkx2.2*‐positive ZLR and ZLC combined with calbindin (CB) immunoreaction (brown). The transition of dorsally placed IGL into the more ventral ZLR and PP *Nkx2.2*‐positive derivatives can be followed relative to the LG and MG nuclei; see also labelled cells migrated into PG (h). The midline lies to the right and caudal is oriented up. (k) *Nkx2.2*‐expressing cells and CB immunoreaction in a transversal section through the caudal subregion of the prethalamus (intersecting the retroflex tract; rf); note relationship of the incertal subcentral region with the tegmental SNC/SNR complex. (l‐o) *Nkx2.2*‐positive prethalamic derivatives in a slightly oblique dorsoventral series of horizontal sections through diencephalon and hypothalamus combined with either CB or TH. Note the superficial *Nkx2.2*‐positive nuclei (PG, IGL, PP), and labelling at the deeper retroincertal nucleus (RI) [Color figure can be viewed at wileyonlinelibrary.com]

**FIGURE 9:**
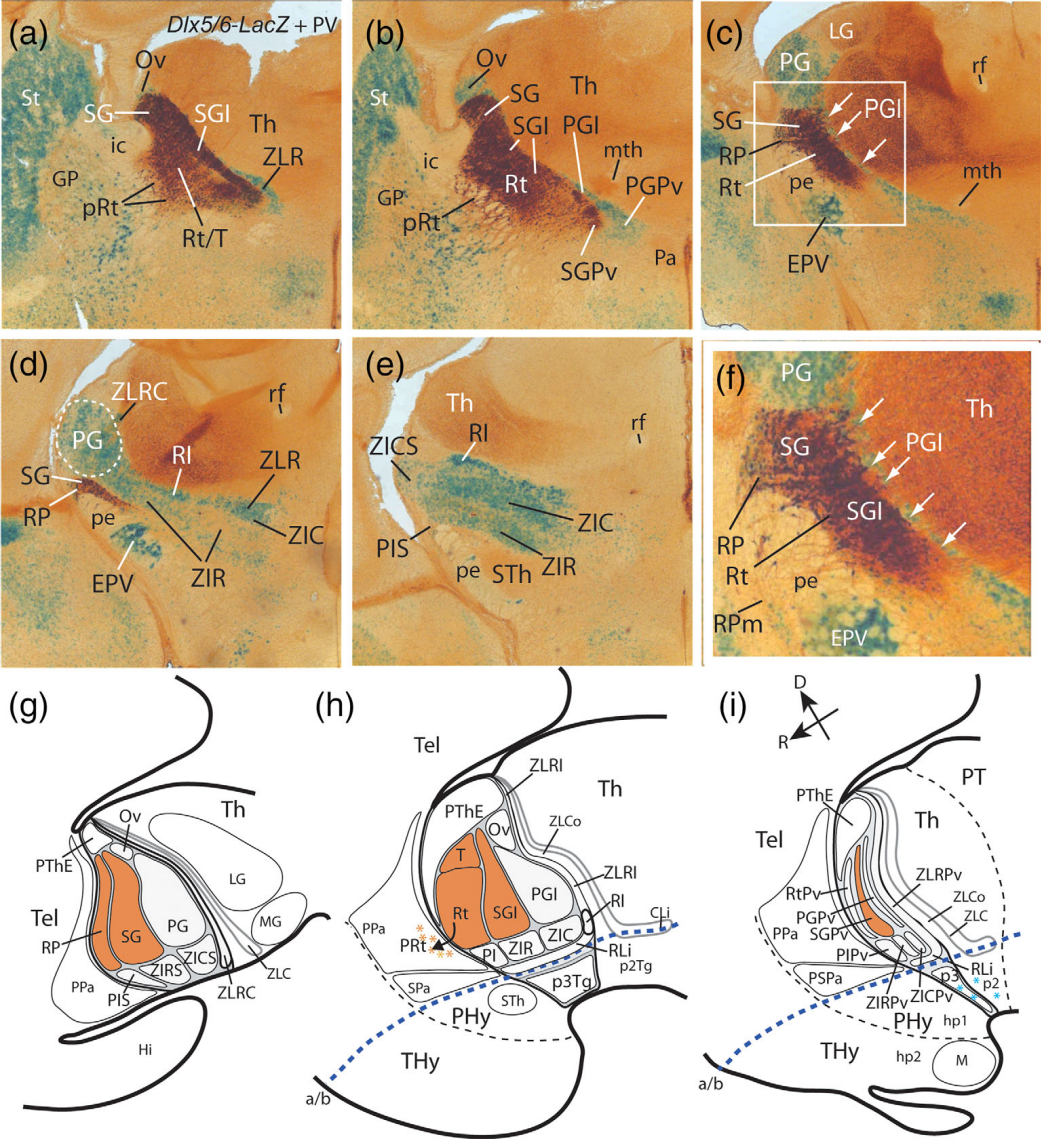
Parvalbumin‐immunoreaction (brown) in the prethalamus of a transgenic P0 mouse brain carrying the *Dlx5/6‐LacZ* construct (blue). (a‐e) Dorsoventral series of horizontal sections through the prethalamus. Caudal is oriented to the top and the midline lies to the right. Note brown parvalbumin (PV) labelling is restricted to the central prethalamus, specifically to its rostral (retropeduncular, reticular, triangular; RP, Rt, T) and middle (subgeniculate nucleus ‐SG, and its intermediate and periventricular strata ‐SGI, SGPv) subregions. Perireticular nucleus (PRt) cells in a,b are also PV‐positive. The caudal pregeniculate complex (PG, PGI, PGPv), the whole subcentral prethalamus (PI, ZIR, ZIC) and the rostral zona limitans are PV‐negative and *Dlx*‐positive. White arrows in both c and the higher magnification shown in f indicate the very narrow intermediate stratum of the pregeniculate nucleus (PGI) which is recognizable by its blue *Dlx*‐positive and PAX6‐negative labelling. (g‐h) Sagittal schemata illustrating in orange the pattern of PV‐labelling observed at the superficial (g), intermediate (h) and periventricular (i) strata. The rostral (R) and dorsal (D) spatial directions are indicated at i [Color figure can be viewed at wileyonlinelibrary.com]

**FIGURE 10:**
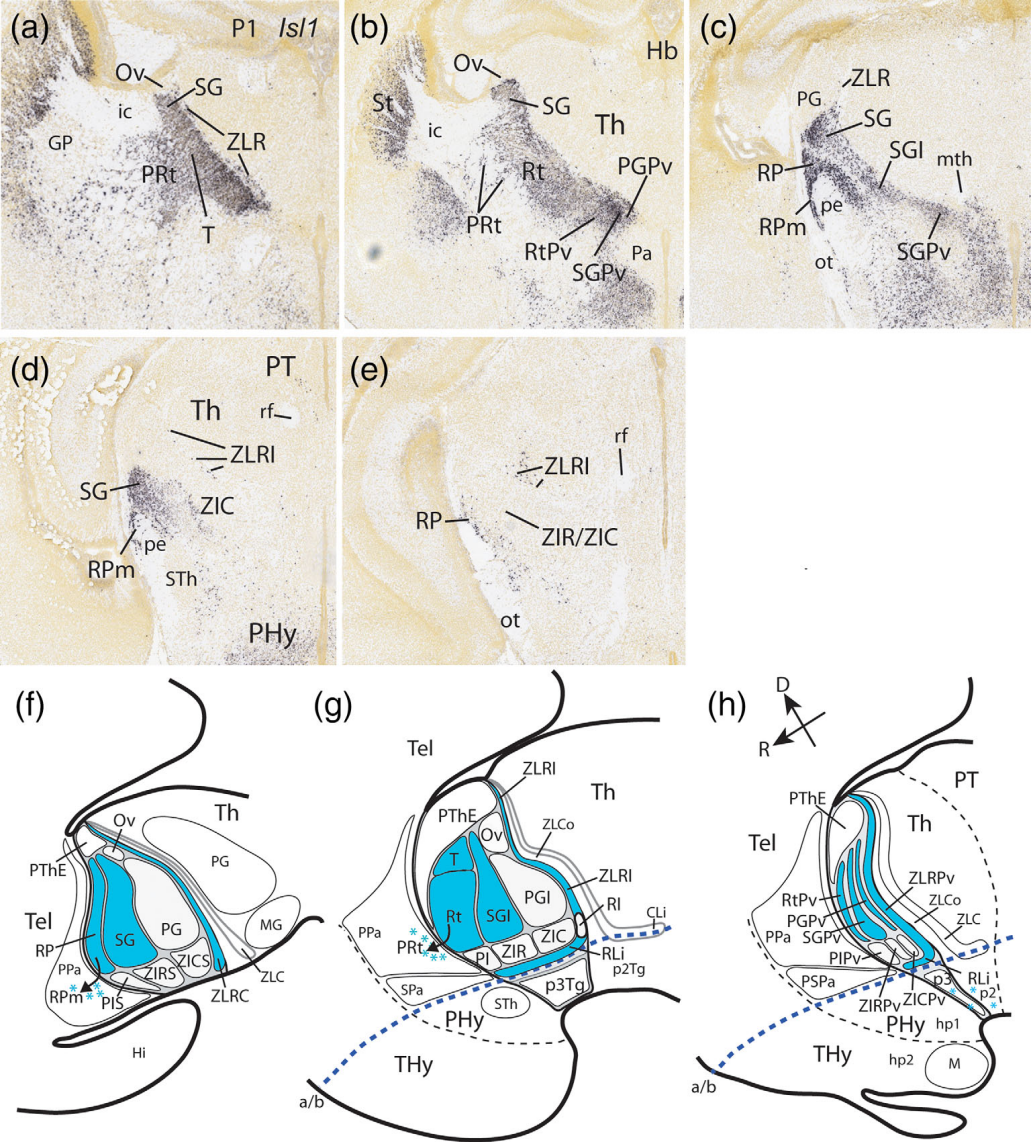
*Isl1* ISH signal in the prethalamus of a P1 mouse brain. (a‐e) Dorsoventral series of horizontal sections through the prethalamus downloaded from the Allen Developing Mouse Brain Atlas. Caudal is at the top and medial to right. The *Isl1* labelling in the prethalamus is rather similar to PV immunoreaction showed in Figure 9. The *Isl1* expression is basically restricted to the central prethalamic subregion, though the ZLR/RLi complex and the PGPv are also labelled. Potential retropeduncular cells migrated subpially to the peduncular paraventricular hypothalamus are identified (RPm in c,d). (f‐g) Sagittal schemata summarizing the *Isl1* expression (blue) in the prethalamus as found at superficial (f), intermediate (g) and periventricular (h) strata. The rostral (R) and dorsal (D) spatial directions are indicated in h [Color figure can be viewed at wileyonlinelibrary.com]

**FIGURE 11:**
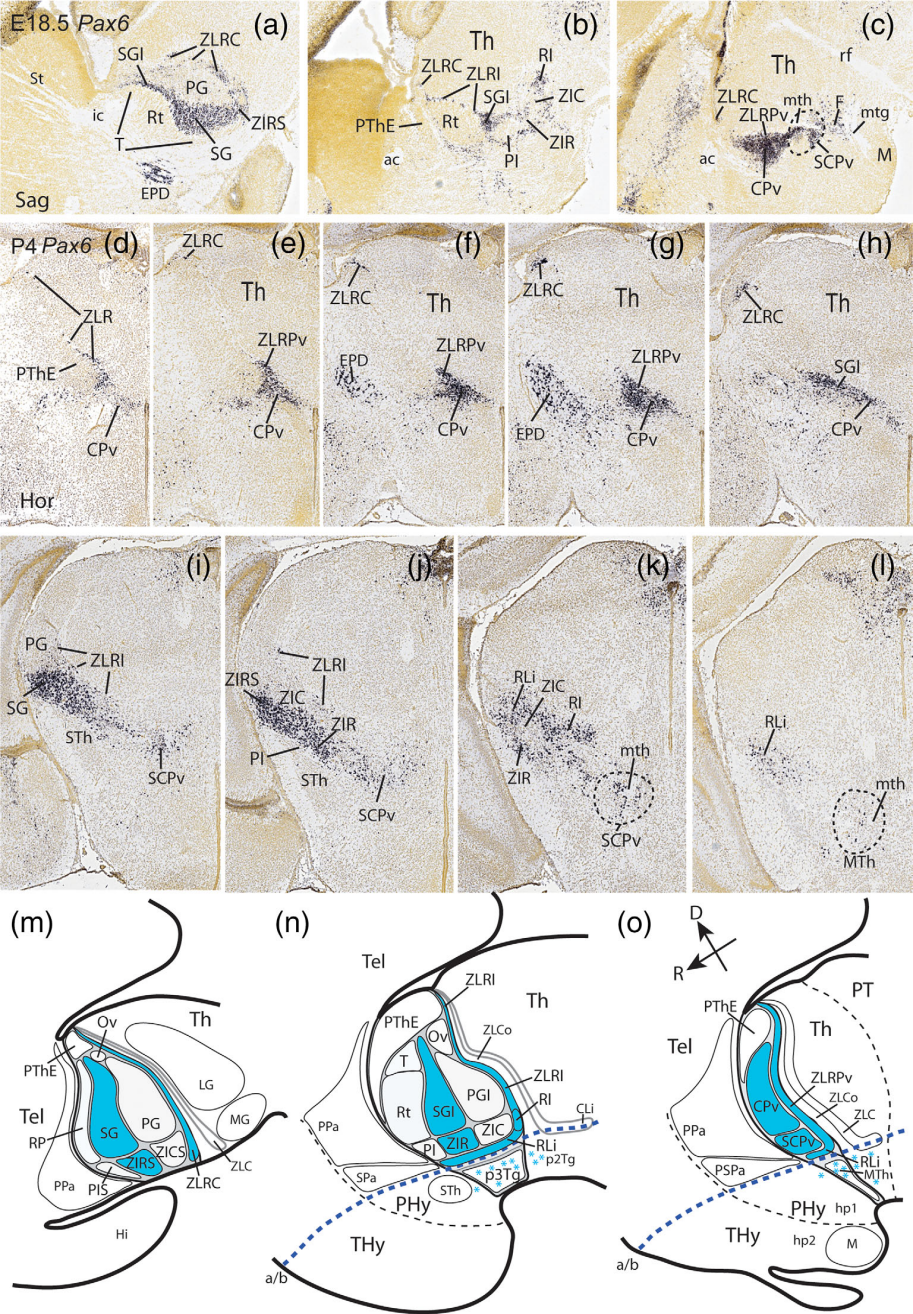
*Pax6* expression in the prethalamus at perinatal stages. All microphotographs were downloaded from the Allen Developing Mouse Brain Atlas. (a‐c) Three lateromedial sagittal sections of a mouse brain at E18.5. Superficially *Pax6* signal is restricted to SG/ZIRS (a) and outer part of ZIR (not shown here; compare i,j), and a line of patches along the ZLRC (a‐c). More medially there is little *Pax6* signal at the inner part of ZIR and ZIC complex (b; compare i,j). Periventricularly there appears a densely *Pax6*‐positive calyx‐shaped cell mass which seems to include all rostrocaudal central parts; we labelled it here as the ‘central periventricular stratum’ (CPv; C). Underneath it there appears a sparsely labelled ‘subcentral periventricular stratum’ above the p3 tegmental field (SCPv; c). (d‐l) Dorsoventral series of horizontal sections through the prethalamus of a P4 mouse brain. Caudal is at the top and medial to right. The *Pax6* labelling is mainly restricted to the middle stratum components of the central and subcentral prethalamic subregions, i.e. the subgeniculate complex (SG, SGI, CPv) and the ventrally located rostral zona incerta (ZIRS, ZIR, SCPv). Note the *Pax6* expression extends to the whole periventricular stratum of the prethalamus, as occurs at early embryonic stages, with the difference that central periventricular elements are fused into a compact CPv locus which reaches the ventricle (CPv; e‐h), whereas the subcentral stratum components jointly form a disaggregated population found at some distance from the ventricle (SCPv; i‐k; compare Fig.1b). (m‐o) Summary of prethalamic *Pax6* expression pattern mapped in blue in sagittal schemata of the superficial (m), intermediate (m) and periventricular (n) strata. The rostral (R) and dorsal (D) spatial directions are indicated in o [Color figure can be viewed at wileyonlinelibrary.com]

**FIGURE 12:**
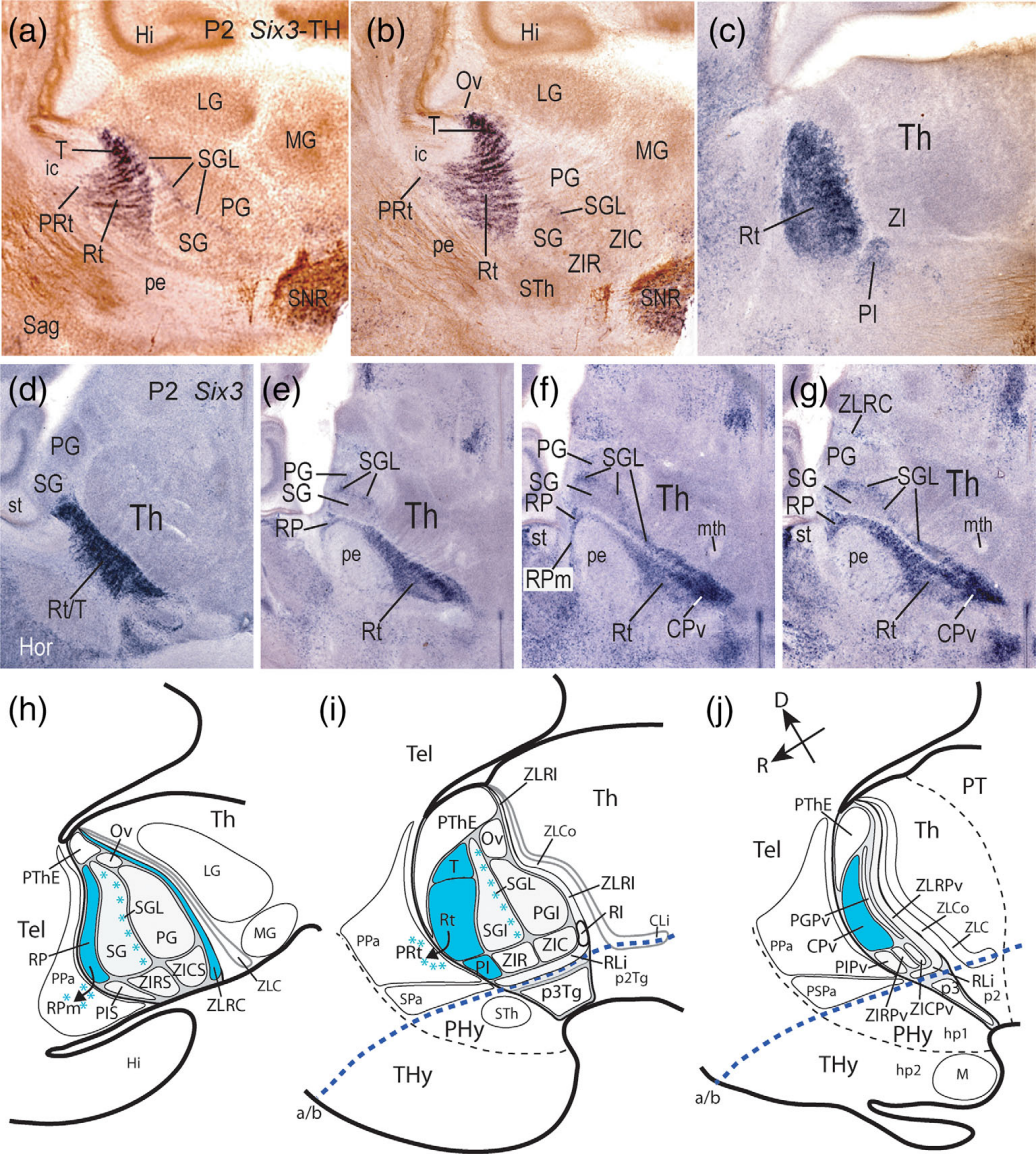
Expression of *Six3* in the prethalamus at P2. (a‐c) Lateromedial series of sagittal sections showing *Six3* labelling mainly restricted to the rostral parts of PThC and PThSC: RP and Rt nuclei, and PI nucleus, respectively. (d‐g) Dorsoventral horizontal sections showing *Six3* expression in the RP/Rt cell populations (subcentral PI not seen at these levels). There is also a thin *Six3*‐positive cell lamina at the caudal part of the superficial and intermediate subgeniculate nuclei (SGL; e‐g). The SGL fuses deeply with the likewise positive RtPv nucleus (f,g), forming the rostral part of the CPv stratum. (h‐j) Summary of *Six3* expression illustrated in blue in sagittal schemata at superficial (h), intermediate (i) and periventricular (j) strata. The rostral (R) and dorsal (D) spatial directions are indicated in j [Color figure can be viewed at wileyonlinelibrary.com]

**FIGURE 13:**
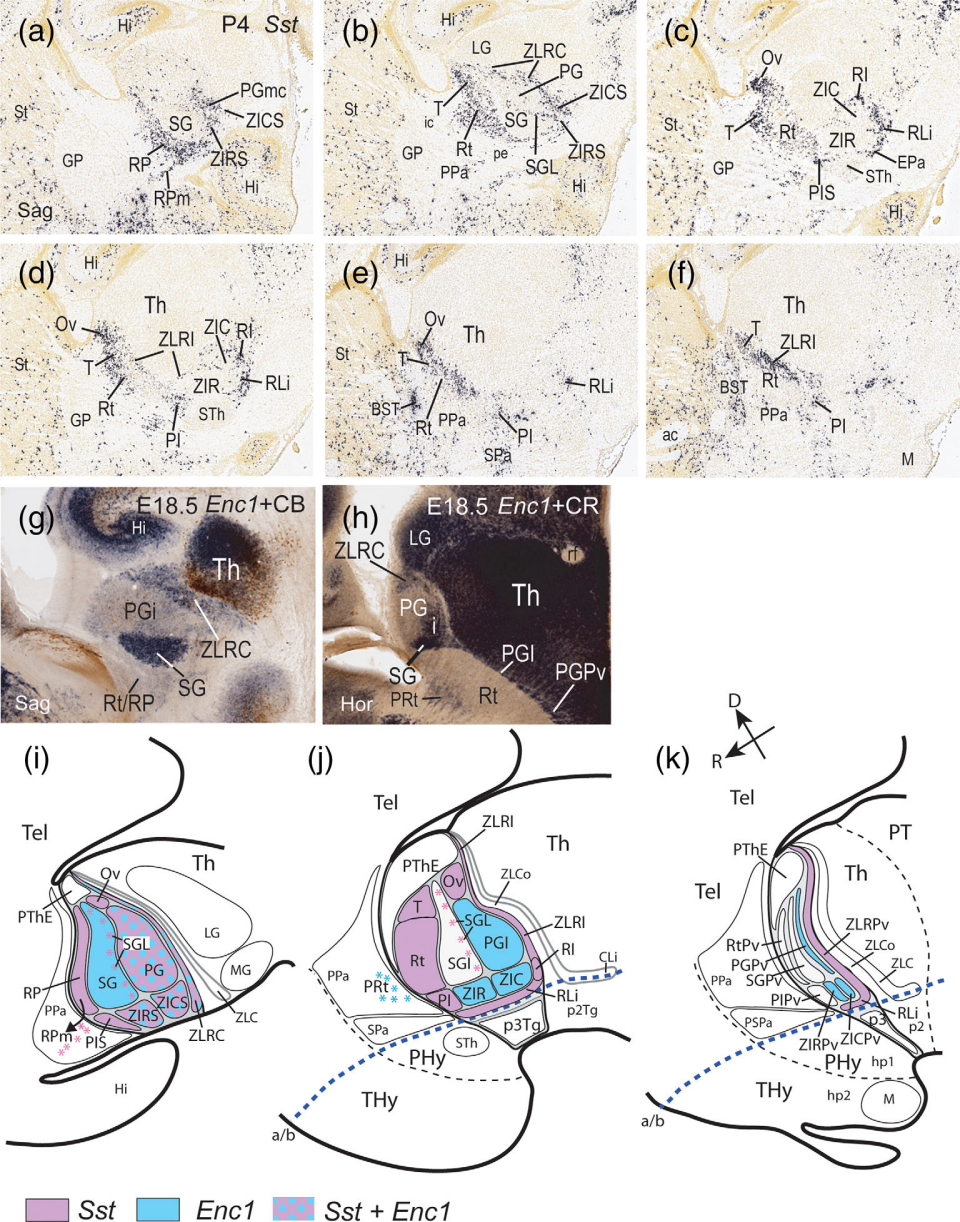
Expression of *Sst* and *Enc1* in the prethalamus at perinatal stages. (a‐f) Lateromedial series of sagittal sections of a P4 mouse brain ISH reacted for *Sst*, downloaded from the Allen Developing Mouse Brain Atlas. Reference axes are indicated in k. *Sst* expression in mainly found at the rostral part of the central prethalamus (RP, Rt, T) and subcentral prethalamus (PIS, PI), but there are also *Sst*‐positive cells at the magnocellular stratum of the PG nucleus (PGmc; a), the SGL caudal lamina (b), the oval nucleus (Ov; c‐e), and the ZLR/RLi complex (b‐e). (g,h) A sagittal section (g) and a horizontal section (h) showing *Enc1* ISH expression (dark blue) combined with immunoreaction against calbindin (brown, CB) in an E18.5 embryonic mouse brain. Note strong and discrete *Enc1* labelling at the subgeniculate nucleus, the internal cell layer of the PG, and the intermediate/periventricular PG strata of PThC (SG, PGi, PGI; g,h). The *Enc1* labelling also extends ventralwards into the rostral and caudal zona incerta (ZIR, ZIC; not shown). The perireticular nucleus, neighboring the unlabelled reticular nucleus, is moderately *Enc1*‐positive (h). The thalamus (Th) is generally strongly *Enc1*‐positive (g,h). (i‐k) Schematic sagittal representation of the *Sst* and *Enc1* in situ hybridization patterns in the prethalamus superficial (i), intermediate (j) and periventricular (k) strata. *Sst* labelled areas in violet, *Enc1* areas in blue, and double‐labelled areas in violet with blue circles or blue with violet circles (i). The rostral (R) and dorsal (D) spatial directions are indicated in k [Color figure can be viewed at wileyonlinelibrary.com]

**FIGURE 14:**
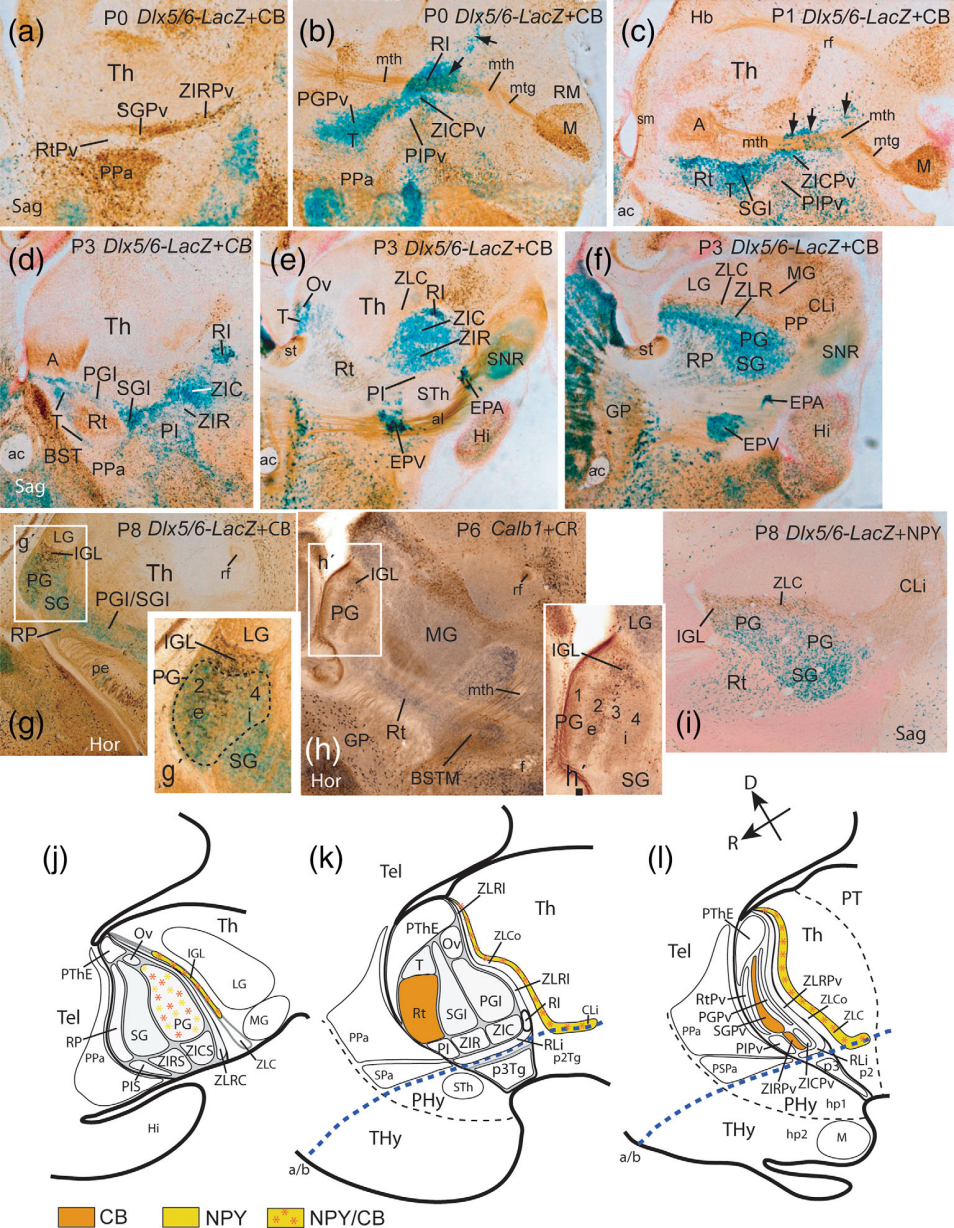
Calbindin (CB) immunoreaction in the prethalamus at postnatal stages. (a‐f) Assorted sagittal sections of *Dlx5/6*‐*LacZ*‐reacted and CB counterstained (IMR) mouse brains at indicated postnatal stages, ordered from medial to lateral. Spatial directions are given below at l. At periventricular level there appears CB reaction restricted to the SGPv and ZIRPv elements, leaving RtPv unlabelled behind the strongly positive hypothalamic PPa nucleus (a). Some fibre packets such as the retroflexus, mamillothalamic, mamillotegmental and ansa lenticularis tracts are CB‐positive (rf, mth, mtg, al). The mamillothalamic tract crosses the ZIC and ZLR at the level of the retroincertal nucleus (ZIC/ZLR, RI) to reach the CB‐positive anterior thalamic complex (b,c). Dispersed blue *Dlx5/6‐LacZ* cells are found in the thalamic tegmental area, caudally to the mth tract (arrows; b,c). The Rt shows a CB‐positive neuropile (d). The *Dlx5/6‐LacZ*‐positive EPV and EPa nuclei appear interstitial to the descending cerebral peduncle, separated by the negative STh nucleus (e,f); note as well thin *LacZ*‐negative limits separating ZIR, ZIC and RI (e), and *Dlx5/6‐LacZ*‐positive lateral part of SNR next to SNL (e,f). The caudal thalamic zona limitans (ZLC) shell component of the superficial IGL is CB‐positive, whereas the ZLR moiety is only *Dlx‐LacZ*‐positive (f). (g) Horizontal section of a *Dlx5/6‐LacZ* P8 mouse brain combined with CB‐immunostaining. The framed area is magnified in g', showing CB‐immunopositive cells spread in layer 2 and 3 of the pregeniculate nucleus (PG); CB cells aggregated at the *Dlx5/6‐LacZ*‐negative part of the intergeniculate leaflet are distinguishable (IGL, g, g'). (h, h') Horizontal section at P6 with ISH expression of the *Calb1* gene (calbindin) counterstained with calretinin (CR) immunoreaction, and magnified detail through framed PG area in h’; note enhanced labelling of PG strata 1‐4 next to IGL. (i) Sagittal section of a *Dlx5/6‐LacZ* mouse brain immunostained for NPY. NPY‐positive cells are concentrated at the thalamic ZLC band, though IGL and PG contain migrated NPY‐positive cells, as reported in the literature (see text). (j‐l) Color‐coded map of CB‐ and NPY‐immunostaining observed at superficial (j), intermediate (k) and periventricular (l) strata of the prethalamus. The rostral (R) and dorsal (D) spatial directions are indicated at l. [Color figure can be viewed at wileyonlinelibrary.com]

**FIGURE 15:**
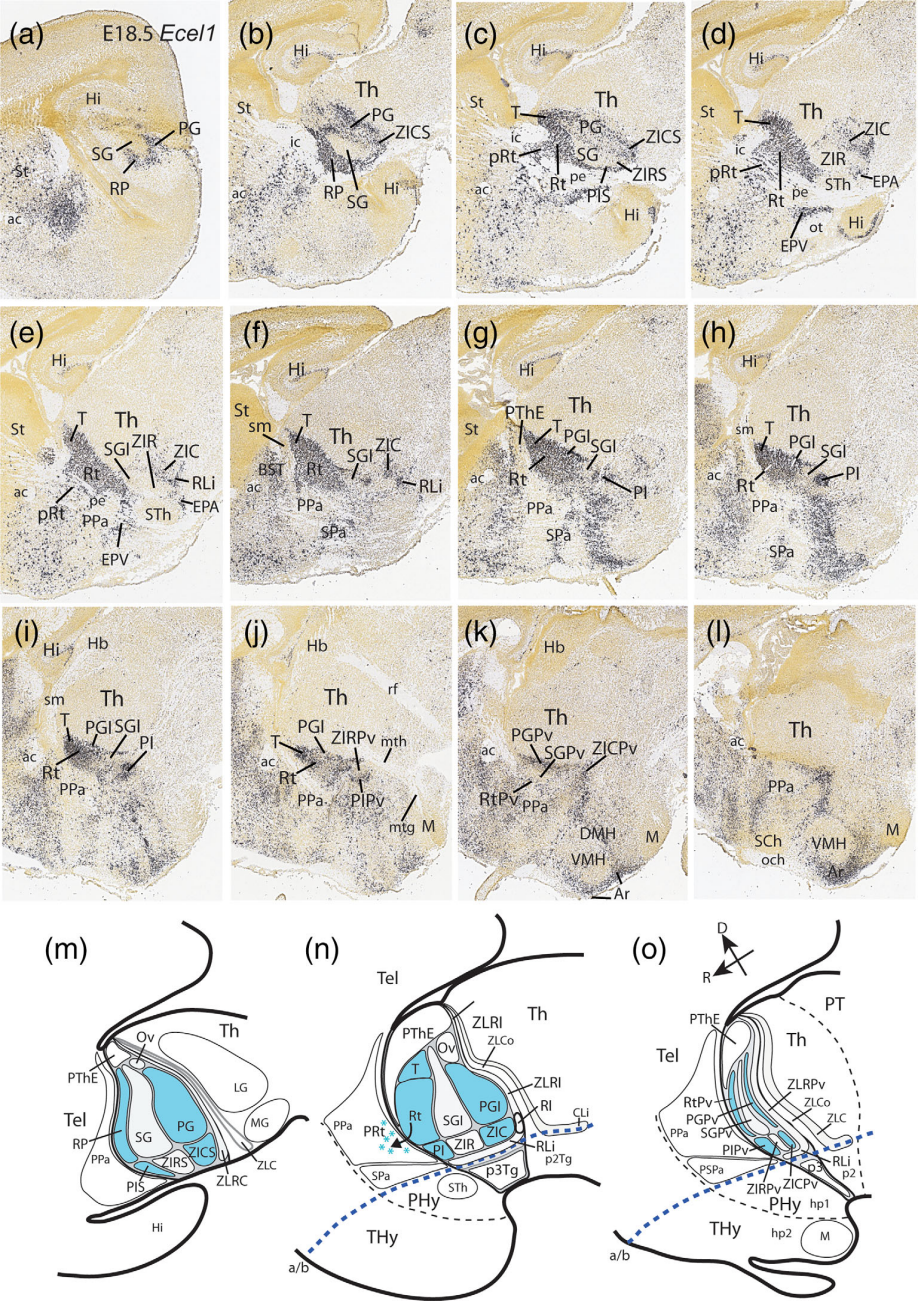
*Ecel1* expression in the prethalamus at E18.5 in images downloaded from the Allen Developing Mouse Brain Atlas. (a‐l) Lateromedial sagittal series of sections showing restricted *Ecel1* labelling at the rostral (RP, Rt, T, PIS, PI) and caudal (e.g., PG, ZICS, ZIC) elements of the central and subcentral prethalamus, contrasting with unlabelled middle elements (e.g., SG, SGI, ZIR). (m‐o) Schematic sagittal representation of *Ecel1* labelling (blue) in superficial (m), intermediate (n) and periventricular (o) strata. The rostral (R) and dorsal (D) spatial directions are indicated at o [Color figure can be viewed at wileyonlinelibrary.com]

**FIGURE 16:**
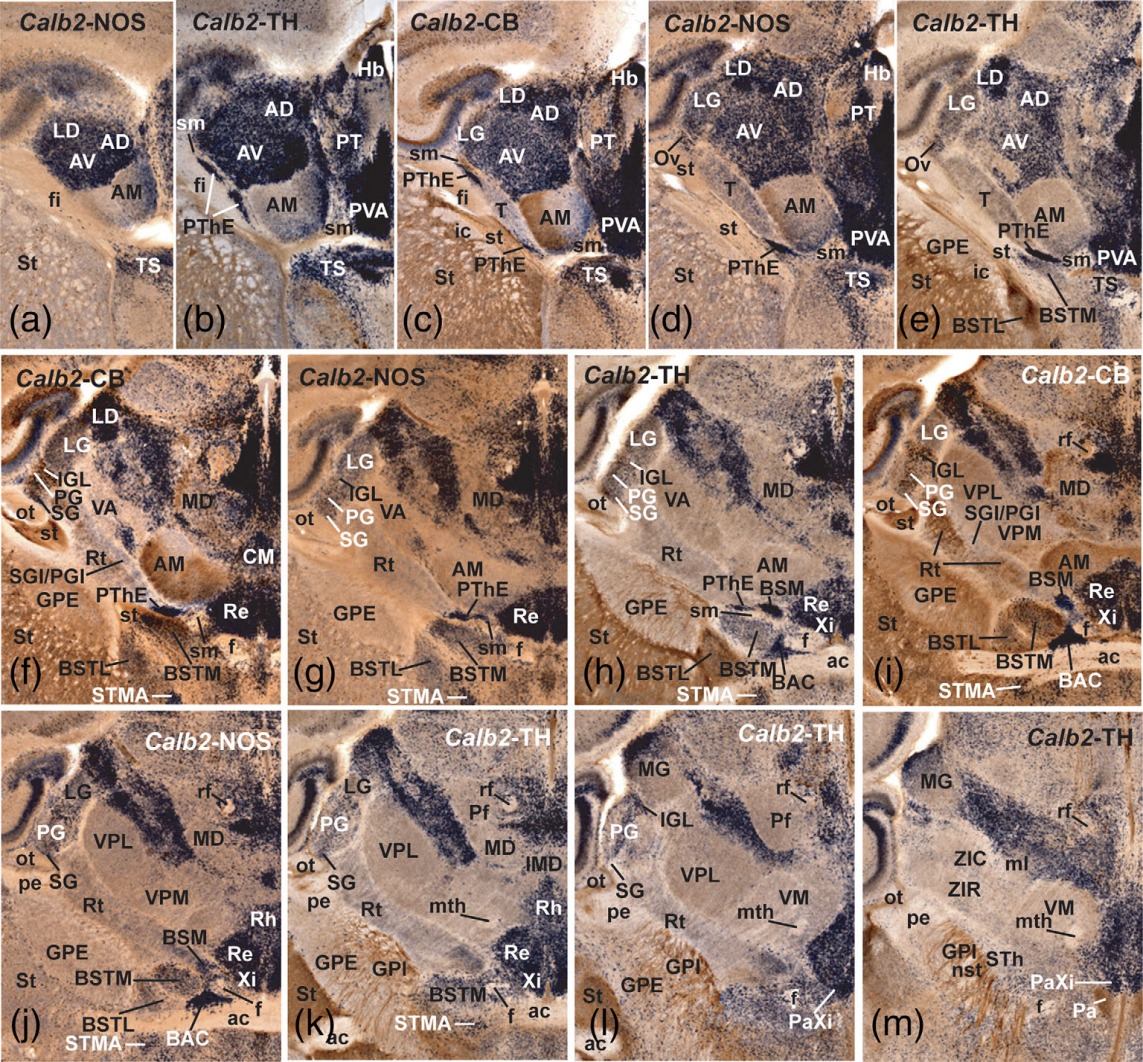
*In situ* hybridization expression of *Calb2* (calretinin; a PThE marker) combined with immunoreaction against NOS, TH and CB (calbindin) in horizontal alternated series of a P40 mouse brain. Caudal is at the top and the midline is oriented to the right. Most thalamic nuclei and the habenula are *Calb2*‐positive, with exceptions (not considered here). In the prethalamus, the *Calb2* signal (dark blue) is restricted to the prethalamic eminence (PThE; b‐h) and its prethalamic derivatives, the bed nucleus of the stria medullaris (BSM; h,i) and the paraxiphoid nucleus (PaXi; l,m), and potentially migrated cell populations such as the bed nucleus of the anterior commissure (BAC; h‐j) and triangular septal nucleus (TS; a‐e). Note also the relation of these PThE *Calb2*‐expressing elements with CB‐positive tracts such as the fimbria, stria terminalis and stria medullaris (fi, st sm). Cells of the reticular nucleus are also CB‐positive (brown in f and i), differentially with regard to the caudally adjacent SGI/PGI nucleus. Note also migrated *Calb2*‐positive cells within the PG nucleus and the IGL (g‐m) [Color figure can be viewed at wileyonlinelibrary.com]

**FIGURE 17:**
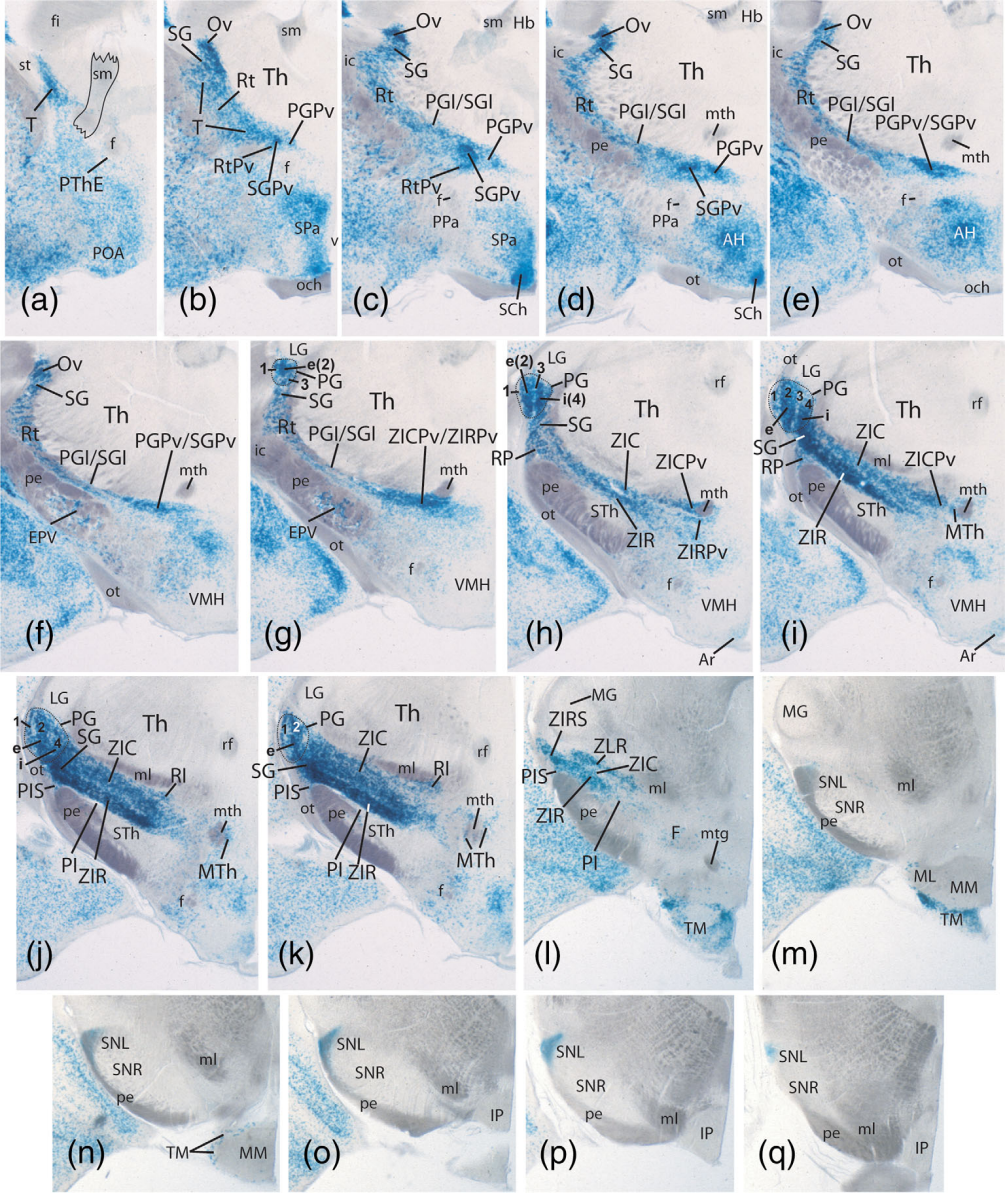
Dorsoventral horizontal section series through the prethalamus of a *Dlx5/6‐LacZ* P40 mouse brain. Midline is to the right and caudal is oriented up. (a‐l) The *Dlx5/6‐LacZ*‐positive prethalamus appears compressed between the *Dlx*‐negative thalamus and partially *Dlx*‐positive hypothalamus, adopting a characteristic sigmoid form. Note the difference between the small and unlayered oval nucleus (Ov; (b‐f)) and the larger and layered pregeniculate nucleus (PG; (g‐k)). The *Dlx5*/*6*‐*LacZ*‐positive periventricular stratum of all prethalamic formations lies farther apart from the ependyma when compared with the P0 stage (see Fig. 2). Myelinated landmark tracts are identifiable as unstained dark gray masses. (m‐q) The ventralmost sections show a packet of *Dlx5*/*6*‐*LacZ*‐labelled fibers of uncertain origin at the lateralmost part of the cerebral peduncle; these fibers seem to end along the lateral part of the substantia nigra (SNL; (n‐q)). The blue hypothalamic cells surrounding rostrally and partly caudally the mamillary body correspond in position and number to described histaminergic tuberomamillary neurons (TM in m,n; Puelles, Martinez‐de‐la‐Torre, Bardet, et al., 2012) [Color figure can be viewed at wileyonlinelibrary.com]

**FIGURE 18:**
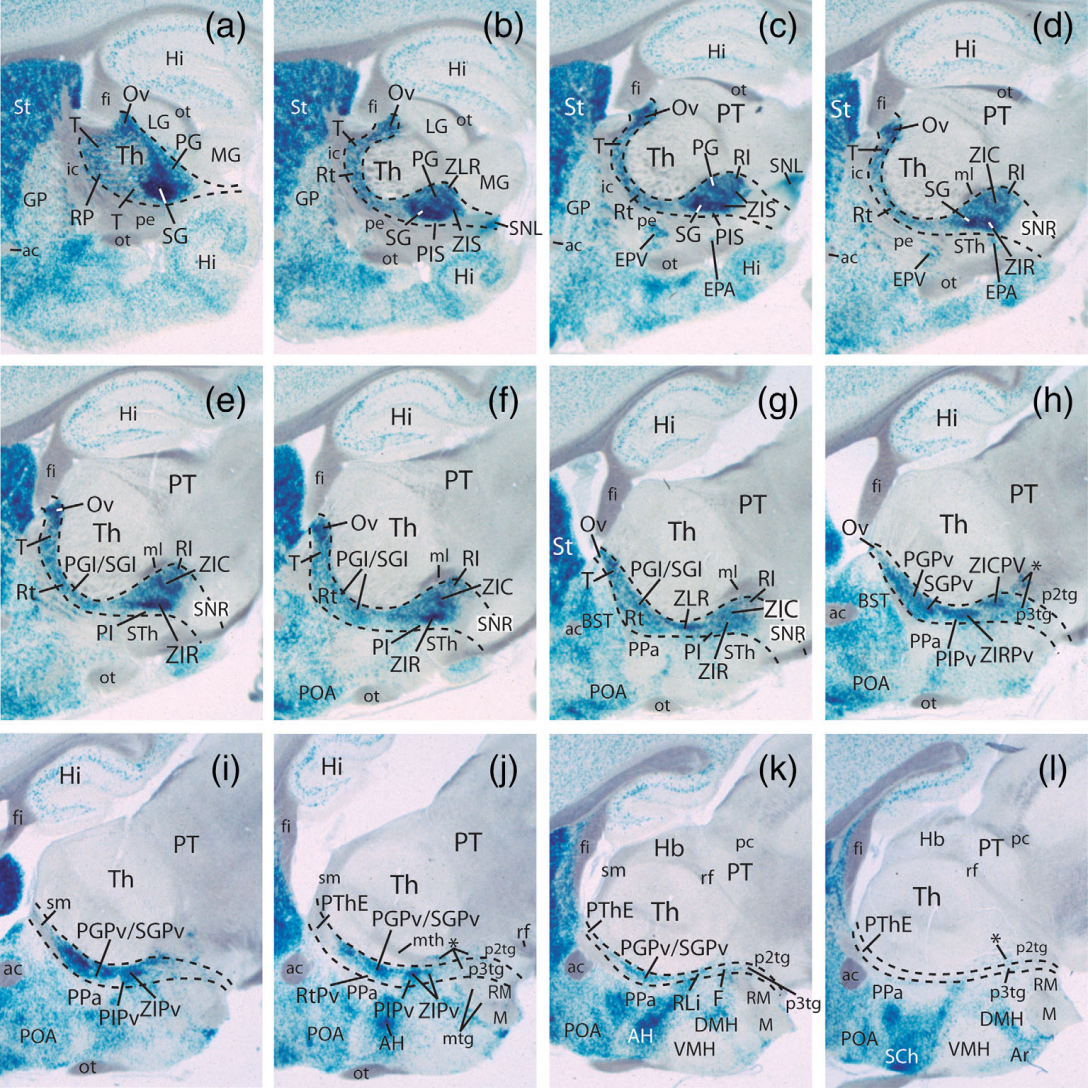
Lateromedial sagittal section series of a P40 mouse brain showing characteristic *Dlx5*/*6*‐*LacZ* labelling in the prethalamus, excepting the prethalamic eminence (PThE). (a‐l) The upper (dorsal) PThC portion including the reticular nucleus (Rt) appears flattened and deformed due to rostral protrusion of thalamic elements (Th), whereas the lower (ventral) PThC plus the PTHSC portion forms a thicker complex apparently lying under the thalamus, though its topologic position continues to be prethalamic (see dash lines indicating the interprosomeric boundaries). The series ends at the significantly rostrocaudally compressed periventricular stratum of the prethalamus (i‐l). Some *Dlx5*/*6*‐*LacZ*‐positive cells appear dispersed in patches within the p3 and the p2 tegmentum, medially to the substantia nigra pars reticulata (asterisks in h‐l). *Dlx* signal is also variously distinguished in the secondary prosencephalon (hypothalamus and subpallium), contrasting with the wholly unlabelled thalamus and pretectum (Th, PT). Unlabelled myelinated tracts are distinguishable as dark gray masses. Note the passage of the stria medullaris tract characterizes the unlabelled PThE region (sm, PThE; (i‐l)) [Color figure can be viewed at wileyonlinelibrary.com]

**FIGURE 19:**
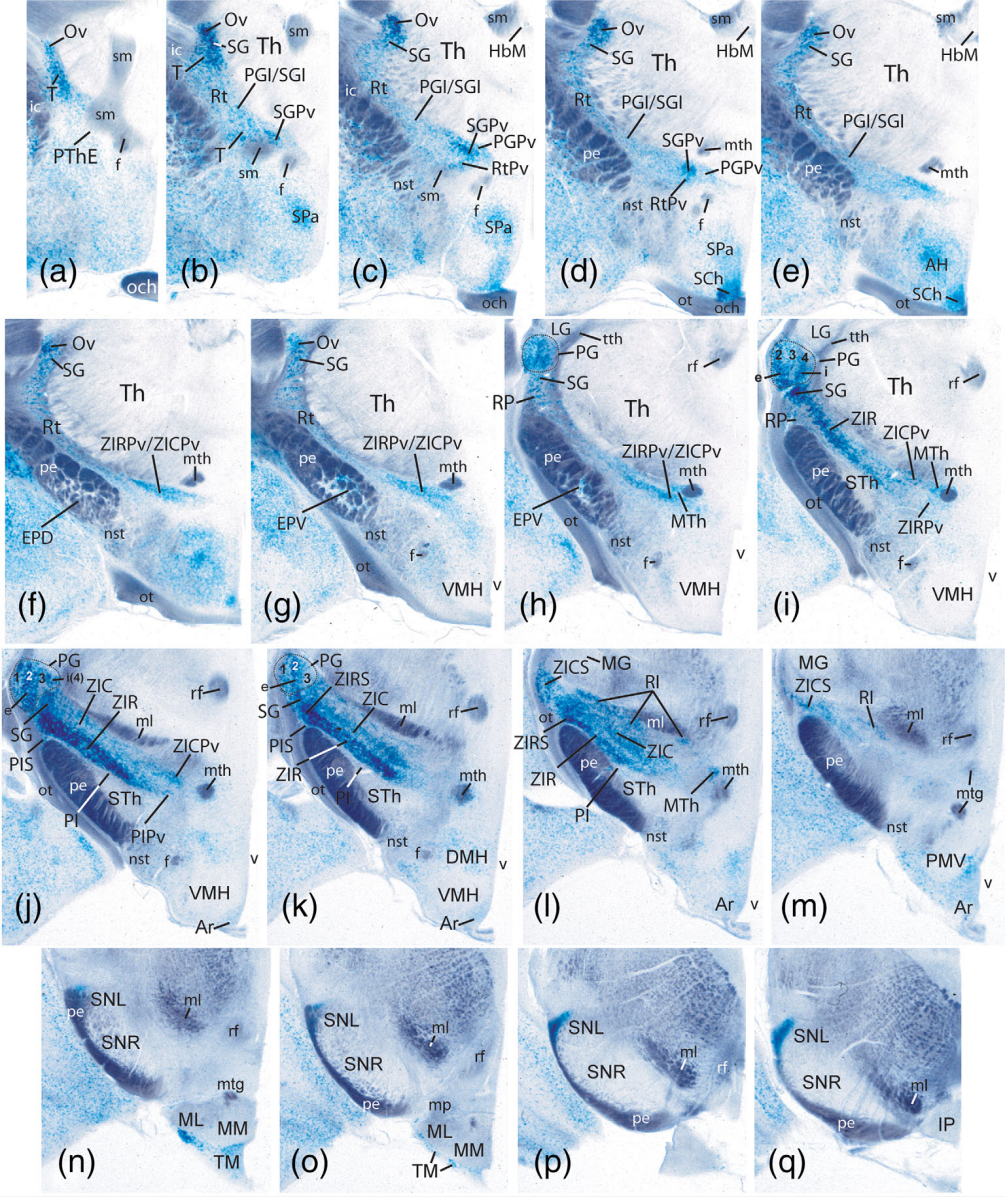
Dorsoventral series of horizontal sections through the prethalamus of a P140 mouse brain. Unstained myelinated tracts identifiable in dark gray are useful as landmarks. (a‐m) The sections show *Dlx5*/*6*‐*LacZ* labelling in the prethalamus and hypothalamus, as well as the unlabelled thalamus (Th). Note small accessory entopeduncular nucleus (EPA; (l)), which shows LacZ signal, similarly as the ventral entopeduncular nucleus (EPV in G,H), contrasting with the *Dlx5*/*6*‐*LacZ*‐negative dorsal entopeduncular nucleus (EPD in F). (l‐q) Ventral sections illustrating *Dlx5*/*6*‐*LacZ*‐positive fibres at the dorsolateral part of the cerebral peduncle, which seem to end next to the lateral substantia nigra (SNL; (n‐q)) [Color figure can be viewed at wileyonlinelibrary.com]

**FIGURE 20:**
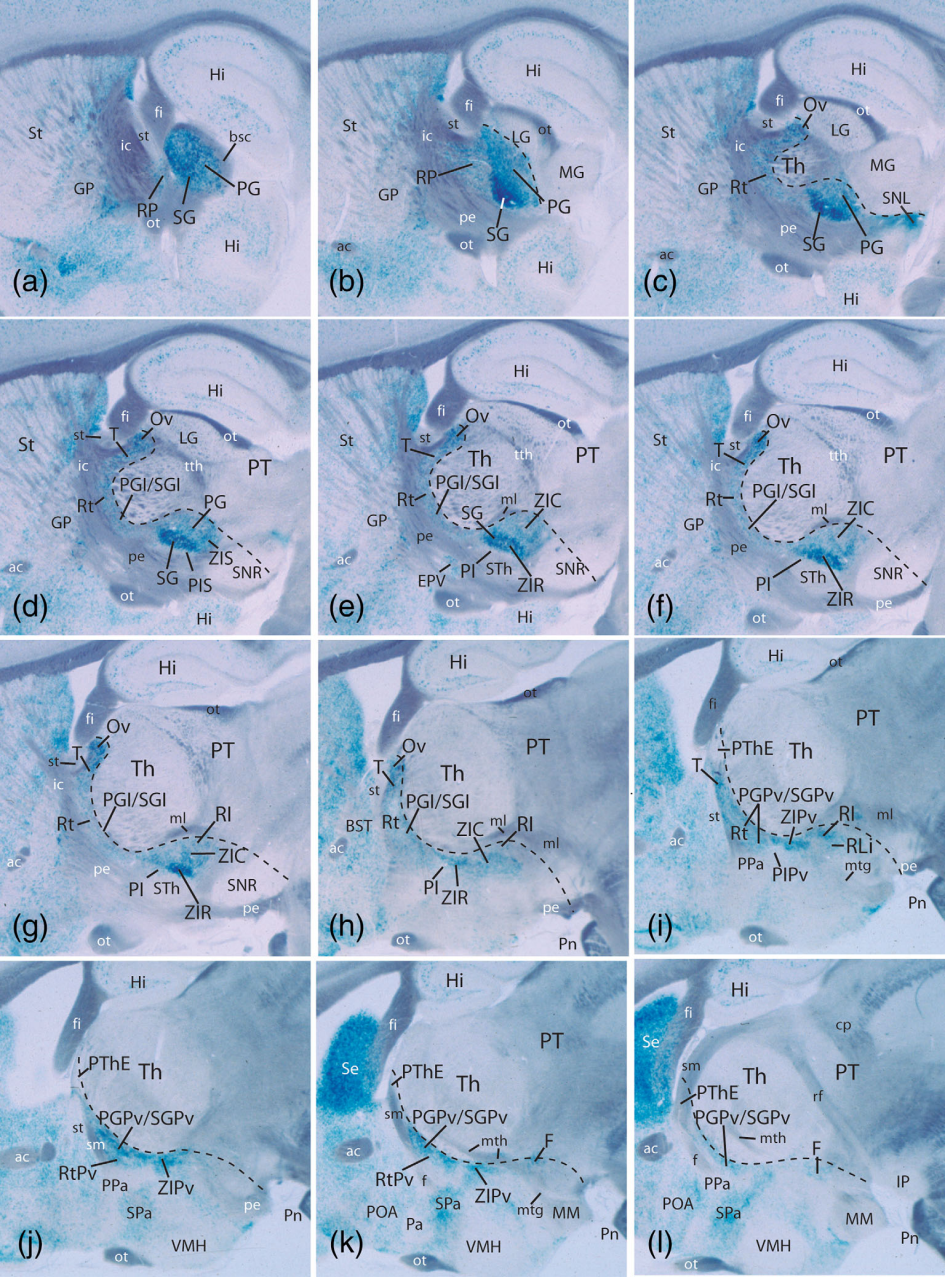
(a‐l) Lateromedial series of sagittal sections through the prethalamus of a P140 mouse brain showing *Dlx5*/*6*‐*LacZ* labelling in blue and myelinated tracts in dark gray. The transversal interthalamic boundary is marked by a dash line. The lateralmost sections are tangent to the PG‐SG‐RP complex, which partly caps laterally the underlying unlabelled Th, due to overall morphogenetic deformation of the diencephalon (a‐c). From there the series expands both into the dorsal, compressed and deformed upper part of the PThC region (Rt, T, Ov, PGI/SGI; (d‐h)), and the thick transition between PG/SG and the incertal complex (PI, ZIR, ZIC; (e‐h)). Note the outer mantle zone of the *Dlx5*/*6*‐*LacZ*‐positive prethalamus is compressed between the thalamus (Th) and the peduncular hypothalamus (PPa), the latter traversed dorsoventrally by the cerebral peduncle, before bending caudalwards past the hypothalamic subthalamic nucleus along the diencephalic substantia nigra (pe, STh, SNR; (b‐g)). The incertal intermediate stratum of the subcentral prethalamus is distinctly wider than the correlative deeper periventricular prethalamic stratum (PIPv, ZiPv, RtPv, PGPv/SGPv; (i‐l)); the latter bounds rostrally with the peduncular paraventricular hypothalamus (PPa; (i‐l)) [Color figure can be viewed at wileyonlinelibrary.com]

Our description below is restricted to PThC and PThSC (plus ZLR/LRi), given that no *Dlx5*/*6‐LacZ* signal appears at the PThE. It is based on *Dlx*‐*LacZ*‐expression observed in several P0 and P4 specimens sectioned in horizontal and sagittal sections (P0, Figures 3 and 4; P4 was not illustrated in extenso, due to its great similarity with P0. Subsequent sections of Results examine *Dlx*‐*LacZ* signal in combination with DLX‐immunoreaction at P0 (Section 3.4 and Figures 5 and 6), *Nkx2.2* labeling (Section 3.5; Figure 7), complementary molecular markers, such as *Calb1*, *Calb2*, *Enc1*, *Isl1*, *Pax6*, *Six3*, and *Sst*, detected by ISH, and CB, CR, NPY and PV, stained by IHC (Section 3.6; Figures 8, 9, 10, 11, 12, 13 and 15), and adult material at P40 (Section 3.7; Figures 16 and 17) and P140 (Section 3.7; Figures 18 and 19). These diverse data are summarized schematically in Figures 20 and 22. Apart of the *Nkx2.2* mappings, several of these materials were downloaded from the Allen Developing Mouse Brain Atlas. We also checked many other data available there (other stages, or other section planes, and additional markers not shown). Finally, another plate (Figure 14) contains E18.5 *Ecel1* expression images downloaded from the Allen Developing Mouse Brain Atlas; these are singularly corroborative of our anteroposterior tripartite PTh interpretation.

A general observation made in newborns and onwards was that *LacZ*‐positive periventricular forebrain elements, and particularly those of p3, gradually became displaced at some distance away from the ventricular lining; that is, a *Dlx*‐negative deep periventricular stratum became apparent (Figure 3). This peculiar feature possibly relates to neuropil maturation within the deep periventricular stratum.

#### 3.3.1 Central prethalamus region (PThC)

In the *Dlx*‐positive PThC region, we distinguish three independent subregions (i.e., molecularly differentially‐specified progenitor domains) in caudo‐rostral order; they are represented by the well‐known *pregeniculate*, *subgeniculate* and *reticular* nuclei (PG, SG, both superficial, and Rt, at intermediate stratum level), respectively, but contain as well additional complementary formations within the radial strata not occupied by these better known nuclei.

In horizontal and sagittal sections through the P0 prethalamus (Figures 3 and 4), the superficial *pregeniculate nucleus* (PG) can be recognized by its strong *Dlx‐LacZ* expression and its well‐known layered structure; its position is superficial (only covered by the optic tract), and it lies caudally within PThC, just rostral to the ZLR and the thalamus. The retinorecipient PG formation was classically named “ventral lateral geniculate nucleus” (Nissl, 1889), consistently with assumptions in the now obsolete columnar forebrain model (Herrick, 1910). This name has now decayed in use with the modern axial paradigm change proposed in the prosomeric model (Puelles & Rubenstein, 2003, 2015), which underpins the alternative *pregeniculate* term. The PG is layered, as is best seen in horizontal and coronal sections. We detect four layers parallel to the overlying optic tract. Superficially there is a weakly labeled *marginal PG layer* (or Layer 1, Figure 3e–k), which corresponds to a superficial cell‐poor retinorecipient neuropil. Underneath the marginal layer there appear two strongly labeled cell‐dense layers—our *external* and *internal PG layers*—(Layers 2 and 4, respectively), which are separated by a cell‐sparse, weakly *LacZ*‐positive gap (Layer 3) (PG; Figure 3c–k); retinal input apparently also occurs in Layer 3 (Major, Rodman, Libedinsky, & Karten, 2003). The two dense *LacZ*‐positive Layers 2 and 4 of the PG clearly correspond respectively to the classically described *magnocellular* and *parvocellular* strata of the adult PG nucleus. Both dense layers thicken caudalwards towards the ZL, where we find a separate, more strongly *Dlx*‐positive, but radially disposed cell aggregate or patch, which we ascribe to the ZLR, since it co‐expresses the *Nkx2.2* marker, among other selective zona limitans shell markers (ZLR; Figure 3j,k; see Martínez‐de‐la‐Torre, Garda, Puelles, & Puelles, 2002; review in Puelles & Martinez, 2013; see also Figure 2). In sagittal sections, the darkly *LacZ*‐labeled pregeniculate nucleus is comma‐shaped, with a rounded ventral end and a tapering dorsal end (e.g., PG; Figure 4d).

At the topologically dorsal end of the PG (which topographically appears somewhat displaced rostralwards at P0), the described layered configuration abruptly disappears and is replaced by a simpler ovoid neuronal aggregate, often misinterpreted in rodent brain atlases as a locus where the Rt nucleus reaches the brain surface (though this is inconsistent with the latter's clearcut intermediate locus). This superficial nonlayered ovoid formation lying just dorsal to the layered PG was identified here as the *oval nucleus*, following comparative evidence supportive of such a nucleus at this locus in nonmammalian tetrapods (Ov; Figures 3a,b and 4i–k; see Section 4). *Sst* is a specific marker of the oval nucleus (Figure 12c–d,i,j).

Rostrally, PG limits with the moderately *LacZ*‐positive, but similarly superficial and retinorecipient *subgeniculate nucleus* (SG). Some sources describing retinal projections do not mention this nucleus (Jones, 2007; pp. 1241–1314), but it does appear for instance in Morin and Studholme (2014) and in the Paxinos and Franklin (2013) mouse brain atlas. In contrast to PG, the SG population is unlayered, and appears as a homogeneous cell aggregate lying just deep to the optic tract at the middle anteroposterior subregion of the PThC. SG is separated from the cerebral peduncle by the equally superficial *Dlx*‐*LacZ*‐positive *retropeduncular nucleus* (RP), which we classify as the superficial stratum that covers the Rt nucleus within the rostral intermediate stratum (SG; RP; Figures 3c–k and 4a–d). The RP essentially lies immediately caudal to the cerebral peduncle (coursing dorsoventrally through the peduncular hypothalamus), but some of its *marginal cells* (RPm) protrude across the superficial diencephalo‐hypothalamic boundary, and extend partially into the hypothalamus, superficially to the peduncle, being covered by the optic tract (RP, RPm, pe, ot; Figures 3h–k and 4a–c).

The intermediate strata of both PG‐ and SG‐related subregions generally appear compacted at levels of maximal development of the Rt into a rather thin, moderately *LacZ*‐positive *intermediate PG*/*SG band*. In our material this band—labeled as PGI and SGI—is distinctly visible caudal to the weakly‐labeled Rt (PGI, SGI; Figures 3h–j and 4e); the PGI component contacts caudally the discontinuous intermediate patches of the ZLR (ZLRI), which typically display a strong *Dlx*‐*LacZ* signal (ZLRI; Figures 3j and 4g,h). We will show below with other markers that the PGI and SGI populations can be labeled differentially from the Rt, contrary to the frequent usage in the literature to lump them all together under the “reticular nucleus” concept. There is various evidence that such an expanded “reticular nucleus” is molecularly and hodologically heterogeneous (see Section 4).

In our opinion, only the rostral subregion of the PThC contains in its intermediate stratum the *reticular nucleus* proper (Rt), which is covered superficially by the already mentioned RP nucleus. As regards its *Dlx*‐*LacZ* profile, the Rt shows much diminished *LacZ* signal at P0 and P4 compared with other prethalamic populations, as a result of early embryonic local downregulation of its primarily present *Dlx*5/6 signal (Rt; Figures 3d–j and 4d–m; we will show below that it nevertheless continues expressing other *Dlx* paralogs). The Rt is a sizeable population in terms of dorsoventral, anteroposterior and radial extent; it is crossed interstitially by the longitudinal components of the thalamic peduncle (thalamo‐cortical and cortico‐thalamic fibers, both reportedly giving collaterals to the Rt), and limits rostrally with the alar lateral hypothalamus, where its closest neighbor is the *LacZ*‐negative *perireticular nucleus*, therefore considered here as an intermediate stratum‐related peduncular hypothalamic entity (see Puelles, Martinez‐de‐la‐Torre, Bardet, et al., 2012). The partial disaggregation of the Rt by passing thalamic and cortical fibers affects particularly its ventral part, where most of these fibers course. There exists also within this rostral intermediate stratum a shell of *LacZ*‐positive cells which cap the perforated Rt rostrodorsally. This *LacZ*‐positive shell area over Rt is entirely comparable to the reptilian triangular nucleus (Díaz, Yanes, Trujillo, & Puelles, 1994; Huber & Crosby, 1926; Medina, Trujillo, Diaz, Martin, & Puelles, 1990). We accordingly propose to apply likewise the name *triangular nucleus* in the mouse and mammals in general (T; Figures 3a–d and 4c–m). *LacZ* signal combined with DLX immunostaining (material described in detail in the next Results section) show differential labeling of the Rt proper versus T, as well as versus the corresponding superficial stratum, the RP nucleus, both of which mostly contain *Dlx5*/*6*‐labeled cells (RP; T; Rt; Figure 5a–g).

The periventricular stratum of the PG and SG subregions largely displays shared labeling properties. It appears in our material as a rather thick and strongly *LacZ*‐positive deep stratum found at some distance from the ventricular lining (PGPv, SGPv; Figures 3c–j and 4m–p). In contrast, the rostral periventricular stratum lying deep to Rt proper shows no *Dlx5*/*6*‐*LacZ* signal, but still distinct pan‐DLX immunoreaction, implying selective postnatal activity of *Dlx1* RNA (RtPv; Figures 3h–j, 4p and 5h; checked at the Allen mouse brain atlas; not shown). Use of additional markers (see below) nevertheless allows differential definition of the periventricular units corresponding to the three PThC subregions. Note some sources misidentify the whole periventricular stratum of the PThC region as either “zona incerta,” or “Forel's field” (Jones, 2007). Actually, all zona incerta parts belong only to the underlying PThSC region, and Forel's field was originally defined as a component of the subthalamic/thalamic tegmentum, that is, the prethalamic/thalamic basal plate (Forel, 1877). The primitive tegmental concept of “subthalamus” was later arbitrarily exported into neighboring alar subregions, including the zona incerta, and sometimes to the whole prethalamus (see historic account and criticism in Puelles, Martinez‐de‐la‐Torre, Bardet, et al., 2012).

#### 3.3.2 Subcentral prethalamus region (PThSC)

The subcentral prethalamic region is likewise subdivided into rostral, middle and caudal subregions, which lie respectively ventral to the corresponding PThC subregions described above, but are distinct from them (Figures 3 and 4). The PThC/PTHSC boundary was not clearly detected in the section planes used in our *Dlx5*/*6*‐*LacZ* material, but a cell‐poor dividing gap is visible in true transversal sections through p3 (e.g., Figure 7k), and can be visualized as well with some alternative gene markers (see below). The histological image of the PThSC is dominated by the conventional *zona incerta* (ZI), a structural complex whose location is restricted to intermediate radial levels of this domain. This region is usually divided into “dorsal” and “ventral” parts, due to use of the arbitrary columnar axis. Prosomeric terminology translates this into *caudal* and *rostral zona incerta* parts; moreover, we add to the incertal complex a newly identified and rostralmost *preincertal nucleus*, which lies directly under the Rt (ZIC; ZIR; PI; Figures 3l–o, 4e–n and 5c–g; compare ZI parts in Puelles, 2013; Puelles, Martinez‐de‐la‐Torre, Bardet, et al., 2012; Puelles & Rubenstein, 1993, 2003, 2015). Caudally, the PThSC contacts via ZIC a particularly thick ventral portion of the strongly *LacZ*‐positive ZLR formation, identified by us as a *retroincertal nucleus* (RI; Figures 3m–p and 4c–k; note this aggregate lies rostral to the thick basis of the ZLCo; see Figure 20b). The ZLR then bends rostralwards into the *rostral liminar* nucleus (RLi), which lies *under* the whole incertal formation (RI, RLi; Figures 1a,d,e, 3p and 4i–n; Puelles & Martinez, 2013).

The *caudal zona incerta* in general expresses strongly *LacZ* (ZIC; Figures 3k–o and 4e–l). Nevertheless, the ZICPv seems to have a stronger signal than the ZIC proper, possibly due to higher cell compaction in absence of passing thalamofugal or thalamopetal fibers (ZIC; ZICPv; Figure 3k–n); the ZIC is wedge‐shaped and diminishes in anteroposterior length from dorsal to ventral sections (ZIC; Figure 3k–o). The ZICPv surrounds the mamillothalamic tract as the latter approaches its thalamic course (ZICPv; mth; Figure 3k–n; compare Figure 14b,c). In contrast, the *rostral zona incerta* intermediate stratum (ZIR) shows a moderate, patchy *LacZ* signal (weaker than that of ZIC), whereas the ZIRPv also displays a strong *LacZ* signal (ZIR; ZIRPv; Figures 3k,l and 4m,n). The ZICS and ZIRS components are not distinguishable in the *Dlx5*/*6‐LacZ* material, but were visualized with DLX‐immunoreaction (see below).

The rostral PI subdomain of PTHSC encompasses at intermediate stratum level the distinct preincertal nucleus (PI), as well as corresponding superficial and periventricular formations (PIS; PIPv). In sagittal sections the PI is an oval shaped field found just rostrally to ZIR, which displays weak *Dlx*‐*LacZ* signal (PI; Figure 4g–l). It is however distinctly pan‐DLX immunoreactive, indicating again persistent postnatal expression of *Dlx1* RNA (Figure 5d–f). The PI is well delimited from surrounding ZIR and RP/SG formations, which display stronger *LacZ* reaction. The corresponding superficial stratum forms rostrally to ZIRS the small *superficial preincertal nucleus* (PIS), which lies directly ventral to the RP nucleus, from which it needs to be distinguished, due to their similar *Dlx* staining characteristics (PIS, RP; Figures 3h–o and 4a–f). Developmental observations indicate that marginal prethalamic subpopulations partly migrate tangentially into the hypothalamus, passing superficially to the peduncle (but underneath the optic tract). We identified them in some images as *marginal* RP (RPm) elements. This superficial partial invasion of hypothalamus appears at horizontal section levels roughly coincident with the locus of the ventral entopeduncular nucleus (RPm, EPV, ot; Figures 3j,k, 4b,c and 5a,b). Periventricular cells lying deep to the intermediate PI nucleus also show a distinct genoarchitectural pattern, though the PIPv is unremarkable in *Dlx*‐*LacZ* material (PIPv; Figures 3l–o and 4m–o; see other data below).

Finally, some groups of *LacZ*‐positive cells appear dispersed in the basal plate of p3 and p2, mostly lateral to the course of the mamillotegmental tract, possibly within the tegmental area originally known as “Forel's field” (p3Tg, p2Tg, F; Figures 3q and 4l–p). We interpret these cells as migrated from the p3 alar plate into p3 tegmentum, with partial subsequent dispersion into the p2 tegmentum.

### 3.4 Comparison of *Dlx5*/*6‐LacZ* and immunocytochemical pan‐DLX labeling patterns

We had at our disposal a series of sagittal sections through the P0 mouse prethalamus labeled with the *Dlx5*/*6*‐*LacZ* reaction which was counterstained by means of brown immunoreaction with a polyclonal antibody that recognizes all forms of the distalless protein (see Materials & Methods). Curiously, this material shows three sorts of results: (a) selective *LacZ*‐labeling (only blue labeling), (b) selective DLX‐immunostained cells (only brown labeling), and (c) double‐stained cells (blue + brown labeling). This material is shown in Figure 5. Our rationale for interpretation of these patterns was explained in Section 2. We summarized in Figure 7 the mixed brown/blue data corresponding to periventricular, intermediate and superficial prethalamic strata across the three AP subregions of the two *Dlx*‐positive DV regions (PThC, PThSC). In general, the anteroposterior grouping of differential *Dlx5*/*6*‐*LacZ* and IHC reactions seems to extend relatively unchanged ventralwards from the PThC into the PThSC, as we examine below. These patterns support the conclusion that there exists at least at perinatal stages a marked *anteroposterior heterogeneity* in the regulated co‐expression of distinct *Dlx* paralog genes in the prethalamus (the same material provides comparable conclusions for the hypothalamus; see Puelles, Martinez‐de‐la‐Torre, Bardet, et al., 2012; fig. 8.15).

**PThC brown labeling**: Within the PThC region, the reticular nucleus is the major element that shows a pure brown labeling, indicating loss of *Dlx5*/*6* signal with selectively persistent *Dlx1*/*2* activity (Rt; Figures 2b–e, 5b–g and 6b; Allen Developing Mouse Brain Atlas); this sort of reaction also characterizes the periventricular stratum of the reticular plus triangular nucleus complex (RtPv; Figures 5h and 6c). In addition, the hypothalamic *perireticular nucleus* also shows the same brown labeling type and *Dlx1*/*2* ISH (PRt; Figures 2a,b,d, 5a–c and 6b).

**PThSC brown labeling**: Within the incertal region, a strong brown labeling (presumed selective DLX1/2 immunoreaction) characterizes the previously unnoticed superficial stratum that covers the main zona incerta nuclei. We used for these structural units the same names applied to the intermediate ZI portions, adding a “superficial” descriptor sign (ZICS, ZIRS, Figures 2a, 5b–d and 6a; note significant lack of *LacZ* reaction at these superficial sites in Figure 3l–p). Rostrally there appears also in strong brown labeling the superficial preincertal nucleus, ventrally to the RP nucleus (PIS; Figures 5b,c and 6a). In addition, the oval‐shaped preincertal nucleus proper (PI), which lies in front of ZIR and deep to PIS, also shows a rather strong brown labeling and *Dlx1*/*2* signal (PI; Figures 2c,e, 5d–f and 6b), shared by its correlative periventricular stratum (PIPv; Figures 5g and 6c).

**PThC blue labeling**: Pure blue sites (indicating low or only prenatal *Dlx5*/*6* signal and undetectable DLX proteins) include the rostrally located retropeduncular (RP, RPm; Figures 5a and 6a) and triangular (T; Figures 5a–g and 6b) nuclei. The middle or subgeniculate PThC subregion also uniformly shows only blue labeling at the superficial subgeniculate nucleus and the underlying thin intermediate lamina of retro‐reticular SGI cells (SG, SGI, Figures 5a–c and 6a,b); there is also deeply to SGI a distinct oval‐shaped *subgeniculate periventricular nucleus*, which also reacts in pure blue (SGPv; Figures 5e–h and 6c). In contrast, selective blue labeling within the caudal or pregeniculate PThC subregion is restricted to the pregeniculate periventricular stratum (PGPv; Figures 5g and 6c).

**PThSC blue labeling**: Pure blue labeling (weak or only early *Dlx5*/*6* and no detectable DLX immunoreaction) appears in the ZIR, found under the similarly labeled SG nucleus (ZIR; Figures 5c–e and 6b).

**PThC double‐labeling**: The double‐labeled PThC elements (showing essentially *Dlx6* ISH plus *Dlx5*/*6*‐*LacZ* reaction; Figure 2f–h; Allen Developing Mouse Brain Atlas) mainly occupy the caudal subdomain, encompassing superficially the pregeniculate nucleus (PG; Figures 2f, 5a,b and 6a) and the small oval nucleus found dorsally to PG (Ov; Figures 5d,e and 6b); note both of them are held to be retinorecipient formations. We observed that the double labeling at the PG is particularly evident at its internal, or parvocellular Layer 4 (PG; Figure 5b), whereas more superficial sections through its external or magnocellular layer (Layer 2) tend to a pure blue labeling (PG; Figure 5a). However, sagittal sections are not optimal to resolve this point.

**PThSC double‐labeling**: Double labeling (only or mainly *Dlx6*; Allen Developing Mouse Brain Atlas) characterizes the ZIC lying under the similarly labeled PG nucleus (ZIC; Figures 2g,h, 5c–f and 6b).

**ZLR/RI/RLi formation**: Caudal to the PThC, the ZLR can be divided likewise into periventricular, intermediate and superficial strata. To avoid confusion with the ZL shell concept and corresponding established tags, we identified the superficial part of this formation, which shows double labeling (*Dlx6*), as the *cap portion* of the ZLR (ZLRC; Figures 5a–c and 6a). In contrast, the intermediate and periventricular parts of the ZLR are only blue (ZLRI; Figures 5c–f and 6b; ZLRPv; Figures 5g and 6c). The ZLRC is represented ventrally and periventricularly, caudal to PThSC, by the RI nucleus, which is continuous rostralward with the analogous subincertal RLi component. Both RI and RLi display a pure blue labeling type (RI; RLi; Figures 5b–g and 6b,c). In the next section of Results (and in Figure 7) we illustrate the ZLR/RI/RLi derivatives at P4, as identified with the selective gene marker *Nkx2.2*.

### 3.5 Analysis of the ZLR/RI/RLi complex at P0/P4 using *Nkx2.2* ISH

The prethalamic alar ZLR/RI/RLi complex appears in the immediate neighborhood of sources of “ventralizing” SHH effects, where *Nkx2.2* is induced (i.e., where SHH concentration is highest; Figure 7a–c). These sources include both the prethalamic longitudinal basal/floor SHH source (relative to which RI/RLi emerge) and the transversal SHH‐releasing core of the alar zona limitans (in front of which ZLR emerges; ZL = the mid‐thalamic secondary organizer, Andreu‐Cervera et al., 2019; Puelles & Martinez, 2013). As a result, this *Dlx*‐positive ZLR/RI/RLi complex displays a peculiar morphologic position occupying both the caudal and ventral limits of the alar prethalamus; a similar effect occurs at the thalamus, leading to a specular ZLC/CLi complex. Both ZLR/RLi and ZLC/CLi can be easily visualized at early embryonic stages as a distinct *Nkx2.2*‐expressing band bordering *Shh*‐expressing tissue (Figure 7a–c; images modified from fig. 8.26B,E,F in Puelles, Martinez‐de‐la‐Torre, Bardet, et al. (2012); see other images and discussion in this source; several other markers as, e.g., *Nkx2.9*; *Ptc*, and *Mash1* reproduce this characteristic expression pattern; others, such as *Ngn1*, give a negative image of the same pattern [Andreu‐Cervera et al., 2019]). Other markers are selective only of the ZLC (e.g., *Six3*) or of the ZLR (*Dbx1* at E11.5). The early *Nkx2.2* expression pattern implies both ventricular and mantle zone labeled cell populations. The parabasal RLi element extends rostrally into the hypothalamus, though there is a sharp pattern change beyond the hypothalamo‐prethalamic border, due to differential hypothalamic tangential cell migrations out of this band (dashed line in Figure 7a–c; see Puelles, Martinez‐de‐la‐Torre, Bardet, et al., 2012).

To the best of our knowledge, there is no published account of the expression pattern of *Nkx2.2*—or any other selective marker of this borderline complex—in the postnatal mouse forebrain. This may have led to the false notion that there is no adult counterpart of the embryonic *Nkx2.2* ISH pattern, or of the embryonic ZLR/RLi and ZLC/CLi correlates. We studied this aspect, focusing on the PTh, in several P4 specimens (*n* = 10) cut in different section planes and counterstained in alternate sections with either Calbindin (CB) or Tyrosine hydroxylase immunoreaction (TH) (Figure 7d–o). It should be noted that *Nkx2.2* apparently labels oligodendrocytes, thus highlighting a uniformly dispersed population of small cells throughout white matter tracts in the brain (e.g., unlabeled in Figure 7; not mentioned further below).

Lateral sagittal sections tangential to the PThC at the level of PG and the thalamic lateral geniculate nucleus (LG) show massive *Nkx2.2* labeling of the intergeniculate leaflet (IGL; Figure 7d,e; compare IGL, LG, PG in horizontal and transversal sections; Figure 7h,k,l,m). We tentatively interpret the IGL band as a fusion of the dorsal parts of the ZLR and ZLC bands; a nearly fused configuration is already observed at dorsal ZL levels at embryonic stages (Figure 7a–c). A thin negative gap separates the dorsal fused IGL from a more rostral and dispersed population of labeled neurons lying within the pregeniculate nucleus (PG; IGL; Figure 7d,e). Horizontal and transversal sections indicate that these probably tangentially migrated PG cells occupy mainly the marginal and external dense cell layers of this nucleus (PGe; IGL; LG; Figure 7h,k–m). The deep PG layers and neighboring SG and Rt areas remain unlabeled. There is also a calbindin‐positive PG subpopulation with a similar distribution (Figures 7h,k,l and 15g,g′,h,h′). We think that the *Nkx2.2*‐ and CB‐positive superficial PG cells probably are IGL derivatives secondarily migrated tangentially (rostrally) into PG; such cells are absent at stages E12.5‐E14.5 (Figure 7a–c). There are literature data suggesting that some of these migrated cells originate at the ZLR (Delaunay et al., 2009), while others come from the ZLC (Delogu et al., 2012).

In contrast, more ventrally, at levels through the medial geniculate body (MG) and the prethalamic ZI region, the IGL bifurcates in an acute angle into a denser rostral component (presumably the now distinct ventral part of ZLR) and a more diffuse caudal labeled population—interpreted here as the conventional *peripeduncular nucleus* (PP) of rodent atlases—which lies just in front of the MG (PP; Figure 7e,f,i,j,n,o). The latter would seem to be the ventral derivative of the thalamic ZLC band (compare PP; Figure 1e).

Deep to the described superficial components there are large stretches of the prethalamo‐thalamic boundary which do not show significant rests of either ZLC or ZLR at P4 (Figure 7e,f,g,l–o). This possibly coincides with passage of tracts afferent to the thalamus or thalamo‐telencephalic fibers (e.g., medial lemniscus; cerebello‐thalamic tract). However, ventrally and medially, a distinct positive band representing the ventralmost transverse ZLR is found, particularly at levels close to the alar‐basal boundary (ZLR; Figure 7e,f,l–o). This medioventral part of ZLR is partly continuous with a particularly dense positive cell aggregate, otherwise closely related to the mamillothalamic tract as it enters the thalamus, which we distinguished as the retroincertal nucleus (RI; Figure 7l,m). Both ventral ZLR and RI are continuous rostralwards with the longitudinally disposed, flat rostral liminar nucleus, which limits PThSC from the underlying tegmental (basal) domain (RLi; bas; Figure 7f,g,j,l,m). An analogous thalamic CLi formation extends caudalwards under the thalamus and pretectum starting behind the PP nucleus (CLi; Figure 7f,g,i–k,n,o; see also Figure 1e).

### 3.6 Complementary molecular characterization of prethalamic populations

In this section, we will describe the perinatal prethalamic distribution of other informative markers in material counterstained or not with *Dlx5*/*6*‐*LacZ* reaction. These data corroborate the prethalamic subdivisions reported above, but also partly highlight some local differences not noticed in our *Dlx* analysis, or even identify singularly some cell populations not recognized previously. This material is illustrated in Figures 8, 9, 10, 11, 12, 13, 14.

#### 3.6.1 Parvalbumin IR

At P0, parvalbumin (PV) immunoreaction is strongly present both at the Rt/T and SG, and extends also into SGI and SGPv (Rt, SG, SGI, SGPv; Figure 8a–d,f,g,i), but is wholly absent at the PG and oval nuclei, including the underlying intermediate and periventricular strata (PG, PGI; PGPv, Ov; Figure 8a–c,f–i). The retropeduncular nucleus (superficial to Rt) also expresses PV strongly, but the marginal cells on top of the peduncle held to have possibly migrated into the hypothalamus are surprisingly PV‐negative (RPm; RP; Figure 8d,d,f,g; see Section 4). In contrast, the perireticular nucleus, which occupies an interstitial position within the neighboring alar hypothalamic course of the peduncle (and reacts to double labeling in pure brown, like Rt; see above), also expresses PV, like Rt (PRt; Figure 8a,b,h). The ZLR formation is also wholly PV‐negative (ZLRC; ZLRI; ZLRPv; RI; RLi; Figure 8). Interestingly, while *Dlx5*/*6* expression patterns were practically continuous in the dorsoventral transition from the PThC into the PThSC, the PV expression pattern is strictly restricted to the mentioned parts of the PThC, leaving the incertal complex largely unlabeled, that is, PV negative (ZIR, ZIC; PI; Figure 8d,e,g–i). PV is therefore a differential marker between PThC and PThSC.

#### 3.6.2 Islet1 ISH

At P1, *Isl1* mRNA expression has a similar pattern as PV as regards the positive Rt/T and RP, as well as SG, SGI and SGPv (all positive; Figure 9a–d,f–h). In contrast to PV, *Isl1* appears expressed at the RPm (Figure 9c–f), and at the periventricular Rt and PG strata, which also was PV‐negative (compare PGPv in Figures 8b and 9b). The ZI complex is largely *Isl1*‐negative (Figure 9d,e,f–h). Finally, the ZLR complex also express distinctly *Isl1* (Figure 9). Accordingly, *Isl1* is also a differential marker for PThC and PThSC, though it shows some differences with the PV marker.

#### 3.6.3 Pax6 ISH and IHC

**FIGURE 21:**
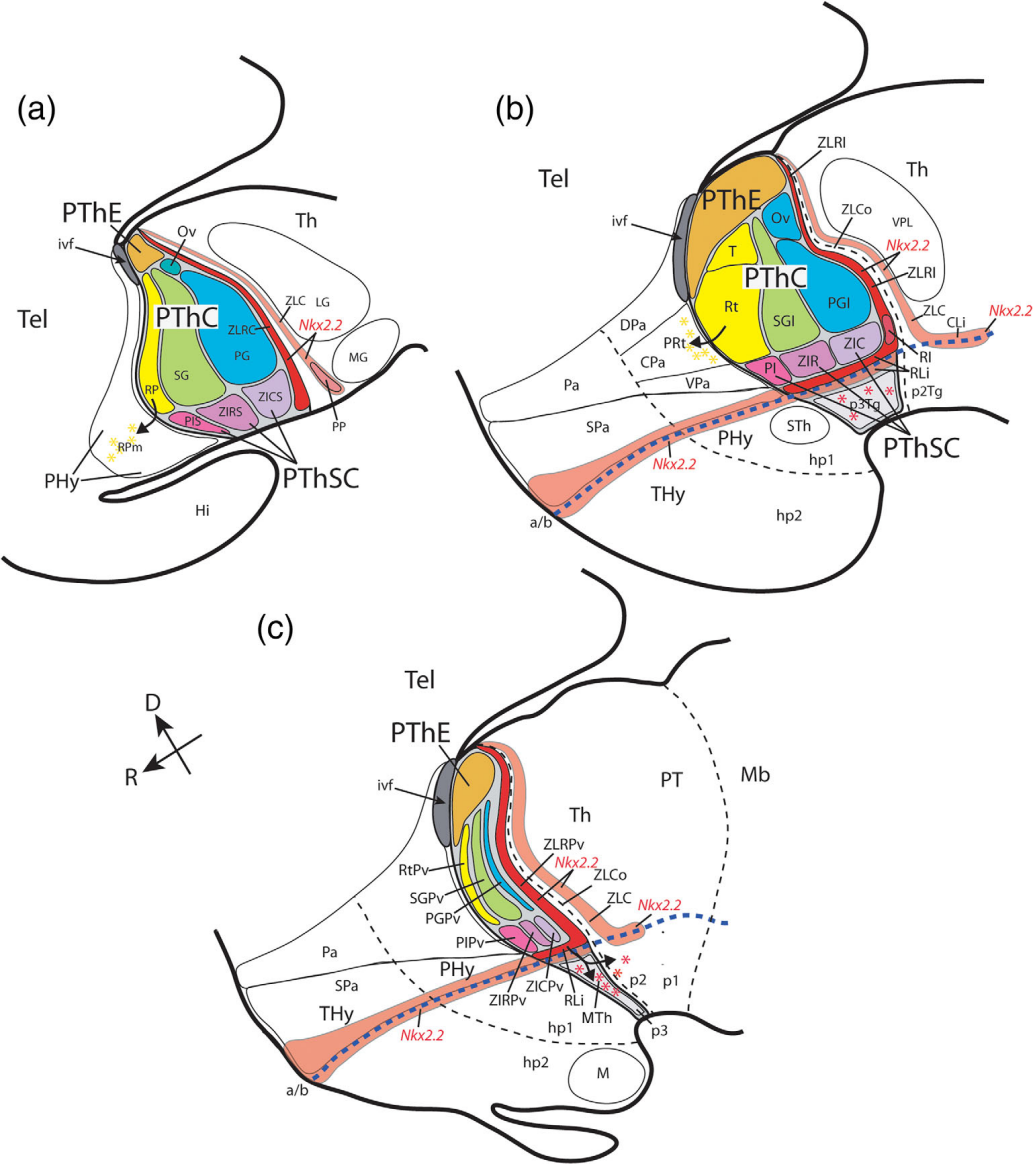
Color‐coded schemata summarizing dorsoventral, rostrocaudal y radial subdivisions of the mouse prethalamus in the context of its nearest hypothalamic and thalamic neighbours. The superficial (a), intermediate (b) and periventricular (c) strata are illustrated separately to show all prethalamic subdivisions and nuclei. The rostral (R) and dorsal (D) spatial directions are indicated in c. Interprosomeric limits are marked as thin black dash‐lines. The longitudinal alar/basal boundary (a/b) is indicated as a thick blue dash line; note its relationship with the pink band where the *Nkx2.2* gene is expressed throughout the forebrain, as well as with the associated zona limitans organizer (ZL core and ZLR/ZLC shell portions). The *Dlx5*/*6*‐*LacZ*‐negative prethalamic eminence (PThE) forms the dorsalmost prethalamic subdivision. The underlying *Dlx5*/*6*‐*LacZ*‐positive prethalamus is subdivided dorsoventrally into central and subcentral subregions (PThC, PThSC), and further includes an alar component of the rostral liminar band (RLi), which co‐expresses *Dlx5*/*6*‐*LacZ* and *Nkx2.2* (in red). Leaving aside the ZLR (in red, continuous ventrally with the RLi), the PThC is rostrocaudally subdivided in rostral, middle and caudal progenitor areas, all of them with radially stratified derivatives; the main rostral derivative is the intermediate reticular nucleus (Rt), whereas the superficial subgeniculate and pregeniculate nuclei characterize particularly the middle and caudal PThC areas (SG, PG). PThSC shows also a tripartite division in preincertal, and rostral/caudal zona incerta subregions (PI, ZIR, ZIC). The most caudal prethalamic subregion corresponds to the rostral *Nkx2.2*‐expressing shell of the zona limitans (ZLRC, ZLRI, ZLRPv; in red). Black arrows and color‐coded asterisks in a‐c indicate apparent tangential migrations of Dlx‐positive cells into either the alar peduncular hypothalamus or the p2 and p3 tegmentum (basal plate). [Color figure can be viewed at wileyonlinelibrary.com]

*Pax6*/PAX6 expression is initially ventricular in the whole p3 alar plate (PThE+PThC+PThSC) up to E14.5‐E15.5 (Allen Developmental Mouse Brain Atlas). Secondarily, *Pax6* signal becomes restricted to middle/rostral parts of the central and subcentral prethalamic mantle layer, while keeping the original ventricular zone expression only at the PThE, where the mantle always is PAX6‐negative (PThE; E13.5; Figure 1a,b; E16.5; Figure 21). Informative *Pax6* ISH mantle data downloaded from the Allen Developing Mouse Brain Atlas are shown for sagittal E18.5 sections (Figure 10a–c), in addition to horizontal P4 sections and graphic schemata (Figure 10d–o). It is readily apparent that the *Pax6*‐positive PThE ventricular zone results progressively reduced in relative size as development advances, though remnants of the PThE ventricular zone are still found in the adult (see below). In contrast, mantle *Pax6*/PAX6 signal is observed strongly and compactly along the whole periventricular stratum of the PThC region from E16.5 (Allen Developing Mouse Brain Atlas; not shown) to perinatal stages (CPv; Figure 10c–h,o). PAX6 immunoreaction is visualized optimally at the RtPv (Figure 21c,d). There is also uniform *Pax6*/PAX6 signal, though slightly less strong, at the SG radial subregion, particularly at the latter's superficial stratum (SG; Figures 1a, 10a,i,m and 21a) and intermediate stratum (SGI; Figure 10a,b,h,n). Due to prenatal dorsoventral morphogenetic deformation of the central complex, its periventricular stratum identified by *Pax6* is found topographically more dorsal than the corresponding intermediate and superficial strata, the latter being distinctly deflected ventralwards (e.g., check the deep CPv, vs. the more superficial SGI, SG areas in Figure 10g,h,i).

The subcentral prethalamus complex shows a *Pax6* expression pattern that reproduces in essence the central one. The main difference is that the *Pax6*‐labeled *subcentral periventricular stratum* is more sparsely populated than the central counterpart, and its cells have lost contact with the ventricular lining (SCPv; Figures 1b and 10c,i–k,o; compare CPv in Figure 10d–h,o). The subcentral periventricular elements that lie topographically ventralmost are disarranged into separate islets by the passage of the mamillothalamic tract (mth; Figure 10c,k–l). Near these ventralmost SCPv *Pax6*‐positive cells other similar elements actually seem to lie within the neighboring hp1, p3 and p2 basal plate areas, where *Dlx5*/*6*‐*LacZ* cells are also observed in sagittal sections; part of these dispersed *Pax6* cells thus seem located in the tegmental field of Forel (F; Figure 10c,n,o). A tegmental *Pax6*‐positive cell group was described before as a constant feature associated topographically to the origin of the mamillothalamic tract from the mamillotegmental *princeps* tract (Skidmore, Waite, Alvarez‐Bolado, Puelles, & Martin, 2012; Szabó et al., 2011; Valverde, García, López‐Mascaraque, & De Carlos, 2000). On the basis of its relationships studied in horizontal sections, we tentatively interpret this cell group as a basally displaced derivative of the alar prethalamic *incertal* periventricular stratum (thus *Pax6*‐positive), which may be conveniently named “*nucleus of the mamillothalamic tract*” (MTh; Figure 10l,o). *Pax6* signal also appears distinctly at the subcentral intermediate stratum, particularly at the rostral zona incerta subdivision (ZIR); this expression site seems continuous with the ventral end of the SG (ZIRS, ZIR; SG; Figures 1a, 10a,b,j,k,m,n and 21b).

The ZLR is also *Pax6*‐positive, though at places the labeling is discontinuous due to passing thalamic fiber packets. Sagittal sections readily show a dorsally tapering transversal band of *Pax6* expressing cells placed caudal to the *Pax6*‐negative PG nucleus and rostral to the negative thalamus; the majority of these cells correspond to our ZLR cap, or superficial ZLR stratum, but others are found at intermediate or periventricular levels (ZLRC: Figures 10a–c,e–h,m and 21a; ZLRI: Figures 10b,i–k,n and 21b; ZLRPv: Figures 10c,e–g,o and 21c). The ZLRI/ZLRC becomes thicker and more populated as it reaches its ventral end, where we identify the retroincertal nucleus (RI; Figure 10k,n), from where the *Pax6* reaction expands rostralwards into the associated longitudinal rostral liminar ZLR continuation (RLi; Figure 10k,l,o).

#### 3.6.4 *Six3* ISH

At early embryonic stages *Six3* is expressed at the *caudal shell* of the zona limitans (ZLC; part of thalamus in p2) and its signal appears also along its longitudinal caudal liminar continuation into pretectum and midbrain (CLi); there is also separate *Six3* expression in an ample area of the rostral prethalamus (Allen Developing Mouse Brain Atlas; LP/MMT, unpublished earlier observations). At postnatal stages, *Six3* expression characterizes in this rostral PTh area predominantly the reticular and triangular nuclei within PThC and the underlying preincertal nucleus within PThSC (Rt; T; PI; Figure 11). These are precisely the main prethalamic formations that are DLX immunoreactive (selective brown labeling; corresponding with *Dlx1*/*2* expression, Figure 2a–e; Allen Developing Mouse Brain Atlas) but *Dlx5*/*6*‐*LacZ*‐negative (no blue signal; see Figures 5 and 6), with the exception of T which contain *Dlx5*/*6*‐*LacZ* blue labeling. Some neighboring central prethalamic elements which only show *Dlx5*/*6*‐*LacZ* blue labeling are instead *Six3*‐negative (e.g., the subgeniculate nucleus; SG; Figure 11a,b,d–i), whereas the retropeduncular nucleus (RP, superficial to Rt) and the related RPm cells are exceptional in showing both *Dlx5*/*6*‐*LacZ* and *Six3* reactions (RP; RPm; Figure 11e–h). The RtPv stratum, jointly with the SGPv, express *Six3* forming a positive rostral part on the central periventricular stratum (CPv; Figure 11f,g,j). Positive *Six3* cells are also found at the perireticular nucleus, though this population lies in the alar lateral hypothalamus (PRt; Figure 11a,b,i). We found distinct *Six3* signal at a thin (previously undetected) caudal cell lamina of the SGI, which thickens ventralwards, and separates the SGI from PGI. This thin lamina limits caudally the entire dorsoventral extent of the subgeniculate domain; we named this new PThC structural element as the “subgeniculate lamina” (SGL; Figure 11a,b,e–i). The *Six3*‐positive SGL is visible only transiently with this marker (between E15.5 and P2) according to data available at the Allen Developing Mouse Brain Atlas.

#### 3.6.5 Somatostatin ISH

At P4, *Sst* signal is found mainly at the rostral PThC subregion which contains the Rt, T, and RP elements, also extending ventrally into the PI formation of rostral PThSC. Strongest reaction occurs at the triangular nucleus and the retropeduncular nucleus (T; RP; Figure S6A–F,I,J); the latter shows a distinct rostral stream of apparently migrated marginal suprapeduncular neurons (RPm) which lie within the neighboring peduncular hypothalamus (RPm; Figure 12a,i; compare Figure 5a). The *Sst* population at the T encapsulates rostrodorsally the upper aspect of the less strongly marked Rt (T; Rt; Figure 12b–f,j) and then converges dorsally into a strongly marked superficial area, which corresponds to the small *oval nucleus* we tentatively identified above (Ov; Figure 12c–e,j; see Section 4). Similar staining was observed at the magnocellular Layer 2 of the PG complex (PGmc; Figure 12a). Other superficial PTh parts such as SG, and the deeper PG layers are *Sst*‐negative. However, *Sst*‐positive cells distinctly populate the caudally limiting *SGI lamina* (SGL; Figure 12b,i,j compare Figure 11a,b). The SGI and PGI subregions of the intermediate PThC stratum separated by the SGI lamina, as well as the T, are conventionally lumped within the adult Rt in the literature. It is interesting that the separate SGL expression of *Sst* jointly with the Rt/T one produces a characteristic streaked *Sst* expression pattern in the lumped adult Rt (LP/MMT; unpublished observations). The preincertal nucleus of the PThSC is also *Sst*‐positive, as indicated above (PIS, PI; Figure 12c–f,i,j). It clearly is discontinuous from the ventral end of the Rt by its exclusive location within the PThSC complex. In contrast, the ZIR and ZIC are only sparsely labeled (ZIRS, ZICS; Figure 12a,b,i). Separately, the ZLR, RI and RLi complex also shows distinct *Sst* labeling (ZLRC; ZLRI, RI; RLi; Figure 12b–f,i–k).

#### 3.6.6 Calbindin IR

It is interesting to examine sagittal sections through the prethalamus in P0, P1, P3 and P8 mice, which carry the *Dlx5*/*6‐LacZ* construct and are also immunoreacted against calbindin (CB) (Figure 13). CB immunoreaction labels landmark tracts such as the mamillothalamic tract, the ansa lenticularis and the stria terminalis in the prethalamic neighborhood (but not the stria medullaris, which is calretinin‐positive) (mth, al, st, sm; Figure 13b–f). A thin SGPv selectively shows CB signal, which extends to the subjacent ZIRPv (SGPv; ZIPv; Figure 13a,l).There is also distinct CB immunoreaction at the Rt intermediate stratum, which contrasts with the negative SGI/PGI stratum (Rt; Figure 13d,e,h,k; SGI/PGI; Figure 13d,g,k). CB labeling of the Rt faithfully reproduces the area with local lack of *LacZ* reaction, whereas no CB was observed at the *LacZ*‐positive T and RP nuclei (Figure 13b–d,f,j,k). A peculiar dispersed CB‐positive subpopulation was observed within the PG, next to labeled CB cells in the thalamic intergeniculate leaflet, a ZLC derivative (PG; IGL; Figure 13g,g′,h,h′,j); we interpret CB‐positive PG cells as possibly tangentially migrated thalamic cells. *LacZ* material immunoreacted for NPY showed an IGL/PG pattern that was very similar to that of CB (Figure 13i,j).

The CB‐positive mamillothalamic tract contains collaterals from the longitudinal basal mamillotegmental tract, which target the anterior thalamic complex (Skidmore et al., 2012; Szabó et al., 2011; Valverde et al., 2000; mth, mtg, A; Figure 13b,c). This tract apparently crosses the base of the zona limitans in its approximation to the thalamic alar field. The CB‐positive fibers of the mth can be seen passing through the particularly voluminous ventral portion of the *LacZ*‐positive RI nucleus (a ZLR derivative) as well as the adjacent ZIC, surrounded by the sparse *Dlx5*/*6‐LacZ* positive cells observed within the neighboring p3 and p2 basal plate (mth; RI; Figure 13b–e). The mth tract finally penetrates the thalamus caudally to the periventricular portion of the retroincertal nucleus (RI; Figure 13b).

#### 3.6.7 *Enc1* ISH

The expression of *Enc1* shows selectivity for both the SG and PG central subregions, as well as the corresponding incertal or subcentral formations, jointly with the ZLR cap zone (ZLRC) (Figure 12g–j). In superficial sagittal sections at E18.5, we observed strong *Enc1* signal at the characteristic middle location and shape of the SG nucleus (SG; Figure 12g,h,i). At this section level there is practically no reaction of the superficial Layer 1 of the PG nucleus, but we see distinct labeling of the ZLR cap domain (ZLRC; Figure 12g). Deeper sagittal sections illustrates the *Enc1*‐positive *internal or parvocellular stratum* of the PG (PGi), which is best visualized in horizontal sections, jointly with the SG and the deeper intermediate and periventricular strata of the PG (PGi; SG; PGI; PGPv; Figure 12h–k). This material clearly reveals a wholly unmarked Rt complex, and, interestingly, also a differentially labeled *perireticular cell population* within the neighboring peduncular hypothalamus (Rt, PRt; Figure 12g,h,j; this is the only marker studied by us that does *not* show parallelism between Rt and PRt).

Labeling at the SG and the deep stratum of the PG extends ventralwards into the subcentral complex, where all radial parts of both rostral and caudal parts of the zona incerta (ZIR; ZIC) show *Enc1*‐positive cell populations. In contrast, the RI and the rostral liminar continuation of the ZLR, the RLi, are negative for *Enc1*, similarly as the PI radial complex (not shown).

#### 3.6.8 Ecel1 ISH

While most markers described above label preferentially rostral or caudal AP structural components of the PThC and PThSC, tending in either case to overlap at the corresponding middle AP subregions), we recently discovered a different *Ecel1* PTh expression pattern which also corroborates independently our subdivision model. This marker labels preferentially both the rostral and caudal subregions of PThC and PThSC at all radial levels, leaving the middle rostrocaudal subregion jointly with the PThE entirely unlabeled. We discovered the *Ecel1* gene serendipitously at the Allen Developing Mouse Brain Atlas; Figure 14 shows this remarkable pattern on E18.5 sagittal images from the indicated source. A rostral field of *Ecel1*‐positive structures includes the RP, PIS, T, Rt, PI, as well as the PRt cells in the alar lateral hypothalamus (Figure 14a–o). Next comes an *Ecel1*‐negative field comprising SG, ZIRS, SGI/SGL, ZIR, SGPv, ZIRPv plus the PThE (same figures), and then a caudal *Ecel1*‐positive field showing signal at the PG, ZICS, PGI, ZIC, PGPv, ZICPv (same figures). Surprisingly, the Ov nucleus, which we expected to appear positive dorsally to PG was *Ecel1*‐negative, perhaps implying it should be ascribed instead to the middle PThC sector (e.g., Ov in Figure 14c,d). It was unclear in the examined material whether the ZLR/RLi complex shows any *Ecel1* labeling, a pattern we have assumed means there probably is no such labeling.

### 3.7 Adult prethalamic cell populations at P40 and P140

In order to provide a full treatment of all prethalamic regions at these postnatal stages we will successively describe first the mature PThE on the basis of *Calb2* (calretinin) ISH at P40 (this being a selective PThE mantle marker) (Figure 16), and next the mature *Dlx*‐expressing PThC and PThSC subdivisions and related tracts of the prethalamus in juvenile (P40) and fully adult mice (P140) (Figures 17, 18, 19 and 20).

At P15 and P40, that is, before the mating age starts (Dutta & Sengupta, 2016), the expression of *Dlx5*/*6*‐*LacZ* in the prethalamic diencephalon (PTh) is still largely comparable to the postnatal pattern, though some changes prelude the adult appearance studied here at P140. For simplicity, only P40 and P140 data are shown (compare Figures 17 and 18 with Figures 19 and 20). The *LacZ* signal is still well visible at these stages, though it is somewhat paler than earlier. The standard derivatives of the original *LacZ*‐positive prethalamic domain are still clearly identifiable, though they are increasingly stretched and bent dorsoventrally and flattened anteroposteriorly, no doubt due to forces created mainly by the growing thalamus. Accordingly, some prethalamic boundaries, particularly those of the AP subregions, become less distinct. The distance between the *Dlx*‐positive “periventricular” PTh formations and the unlabeled diencephalic ependyma increases slightly during this period, so that most blue cells are found external to (i.e., more superficial than) the periventricular limiting plane defined by transverse landmark fiber tracts, such as the retroflex, mamillothalamic and fornix tracts (rf, mth, f; Figures 17 and 19).

The relative dimensions of the diencephalic alar domains evolve differentially. The periventricular stratum of alar p3 suffers progressive compression between the spherically expansive p2 alar mantle (thalamus) and the alar peduncular hypothalamus, which contains the paraventricular nucleus and the lateral hypothalamus, plus the massive cerebral peduncle (i.e., the medial and lateral forebrain bundles; Puelles, Martinez‐de‐la‐Torre, Bardet, et al., 2012). The original *Dlx*‐positive periventricular and intermediate elements of alar p3 thus result compressed anteroposteriorly into a concave capsule surrounding rostrally and laterally the thalamic mass (Puelles, Martinez‐de‐la‐Torre, Ferran, et al., 2012). The medial (deep) and lateral (superficial) parts of the deformed PTh are thereby pushed out into relatively more lateral and caudal topographic positions, apparently leaving next to the ventricle only a cell poor *Dlx*‐negative periventricular stratum (possibly downregulated *Dlx* signal?). Sagittal sections also witness a progressive sigmoid incurvation of the zona limitans boundary, duplicated at the large Rt population, and the PTh as a whole (Figures 17 and 19). This seems a result of the disproportionate growth and varying rostral protrusion of diverse nuclear parts of the thalamus (Puelles, Martinez‐de‐la‐Torre, Ferran, et al., 2012). Moreover, at these stages, unstained myelinated landmark tracts become increasingly visible as dark gray masses in our material, due to light diffraction.

#### 3.7.1 Prethalamic eminence (PThE)

We had not given previously any details about the PThE because of the absolute lack of *Dlx* expression, *LacZ* reaction and pan‐distalless immunoreaction at this locus. This hyperdorsal regional constituent of the prethalamus remains negative for *Dlx5*/6‐*LacZ* at P40 and P140, as it was before (PThE; Figures 16a, 17j–l, 19a and 20i–l). Recognition of this apparently progressively reduced domain in the adult rodent brain is classically difficult, leading often to its misidentification as either a part of the “bed nucleus striae terminalis complex,” or, more often, as “reticular nucleus.” For instance, the PThE is not identified as either “thalamic eminence” (the misleading classic name) or the newly orthodox “prethalamic eminence” in the mouse brain atlases of Hof, Young, Bloom, Belichenko, and Celio (2000), Watson and Paxinos (2010), or Paxinos and Franklin (2013). These authors nevertheless do recognize in this area the stria medullaris tract (sm), known to course longitudinally through the PThE area before it enters the habenula (Puelles, Martinez‐de‐la‐Torre, Bardet, et al., 2012; fig. 8.34B). Next to the sm tract a “bed nucleus of the stria medullaris” (BSM) is sometimes identified in rodent brain atlases. This nucleus may well be the chief homotopic derivative of the embryonic PThE. However, there is evidence that some other PThE derivatives result ectopically placed, due to their embryonic tangential migration out of the PThE primordium in rostralward direction, that is, into telencephalic and hypothalamic loci (Alonso, Trujillo, & Puelles, 2020; Watanabe, Irie, Hanashima, Takebayashi, & Sato, 2018).

At embryonic stages, the marker calretinin appears to characterize rather selectively the PThE mantle (Abbott & Jacobowitz, 1999), in contrast with the neighboring intratelencephalic BST complex, whose cells instead express strongly calbindin (Jacobowitz & Abbott, 1997; Medina & Abellán, 2012; fig. 7.10G). The underlying prethalamic Rt shows no calretinin and little calbindin staining (Jacobowitz & Abbott, 1997; Puelles, Sánchez, Spreafico, & Fairén, 1992). In order to delineate potentially dispersed PThE remnants, we examined a P40 mouse brain which was ISH reacted for calretinin (*Calb2*) in floating horizontal sections (cut parallel to the optic tract), of which alternate sections were counterstained by immunoreaction for CB, NOS or TH. This material is illustrated in Figure 16.

Eminential calretinin ISH signal was first observed at a dorsal horizontal level passing just underneath the hippocampal fimbria, where this tract arches medialwards into the hippocampal commissure (fi, PThE; Figure 15a–c). The PThE is represented by one or two elongated patches of strong calretinin signal found within supracapsular (=above the internal capsule) prethalamic territory, and found rostrolateral to the clearly delimited habenula and thalamus regions; the latter includes the anterior periventricular, parataenial, anterior and laterodorsal thalamic nuclei (PThE, Hb, PVA, PT, AD, AV, LD; Figure 15a–e). As the horizontal sections penetrate more deeply into the prethalamus, the dense eminential calretinin ISH signal becomes restricted to the apparent frontal and caudal ends of the supracapsular prethalamic territory (in our interpretation, these positive ends represent morphogenetically deformed medial/deep versus lateral/superficial eminential parts). The separation of extreme deep and superficial PThE parts leaves an intermediate space occupied by the underlying prethalamic triangular nucleus, which caps the reticular nucleus; the newly recognized oval nucleus soon appears next to the superficial (laterocaudal) tip of the PThE patch (PThE, T, Ov; Figure 15a–e). The PThE and neighboring dorsal PThC parts of prethalamus (mainly T) jointly form at these levels an oblique band intercalated between the interthalamic boundary (ZL) and the neighboring stria terminalis tract (st) plus associated subpallial nuclei (compare Paxinos & Watson, 2014 rat brain atlas figs. 200–201, where such a dorsal PThE+T band—according to us—is identified as “reticular nucleus”). In contrast, the telencephalic BST elements, which are calbindin‐immunoreactive and contain sparse calretinin‐positive cells (BSTM, BSTL; Figure 15e–k), represent periventricular elements of the medial ganglionic eminence and therefore lie topologically *under* the interventricular foramen (Puelles, Medina, et al., 2016; Puelles, Morales‐Delgado, et al., 2016; Puelles & Martinez, 2013). They are found always immediately rostral to the homotopic PThE remnant (or BSM) and its associated stria medullaris tract; the latter remains visible down to section levels through the anterior commissure, where it lies lateral to the fornix tract (PThE, BSTM, sm, f, ac; Figure 15e–k). At even more ventral section levels, the *paraxiphoid nucleus* (PaXi), which we tentatively ascribe to periventricular prethalamus, also expresses densely calretinin, consistently with an eminential origin. It constitutes the topologically deformed (compressed) ventralmost part of the eminential periventricular stratum, which lies strictly dorsal to the deep PThC stratum. In horizontal sections the PaXi appears intercalated between the thalamic reuniens and xyphoid nuclei (caudally) and the hypothalamic paraventricular nuclear complex (rostrally) (PaXi, Re, Xi, Pa; Figure 15k–m). Paxinos and Watson (2014) correctly classified the PaXi nucleus within the prethalamus, but apparently did not explicitly ascribe it to PThE (their atlas figs. 162 and 163).

Migrated ectopic eminential derivatives are thought to include at least the sizeable *septal triangular nucleus* (TS; Figure 15a–e) and the *nucleus of the anterior commissure* (BAC; Figure 16h–j; see Watanabe et al., 2018), both of which are distinctly calretinin‐positive in our preparations; the BAC appears connected directly with the PThE by a calretinin‐expressing cell bridge which passes between the fornix tract and the BSTM nucleus (BAC, f, BSTM; Figure 15h–j). Other nearby paraseptal cells such as the “STMA” (medial anterior stria terminalis nucleus) identified by Paxinos and Watson (2014) in the rat also show a PThE‐like calretinin‐positive profile, and stand out as a distinct mass partly embedded within the BST complex. This mass accompanies for a while the rostrally diverging rostral branch of the anterior commissure, and apparently connects with the BAC just behind the anterior commissure (STMA; Figure 15f–k). A more complete listing of apparently migrated derivatives of the avian PThE homolog (probably also present in the mouse) was recently reported by Alonso et al. (2020).

#### 3.7.2 Central prethalamic region (PThC)

**FIGURE 22:**
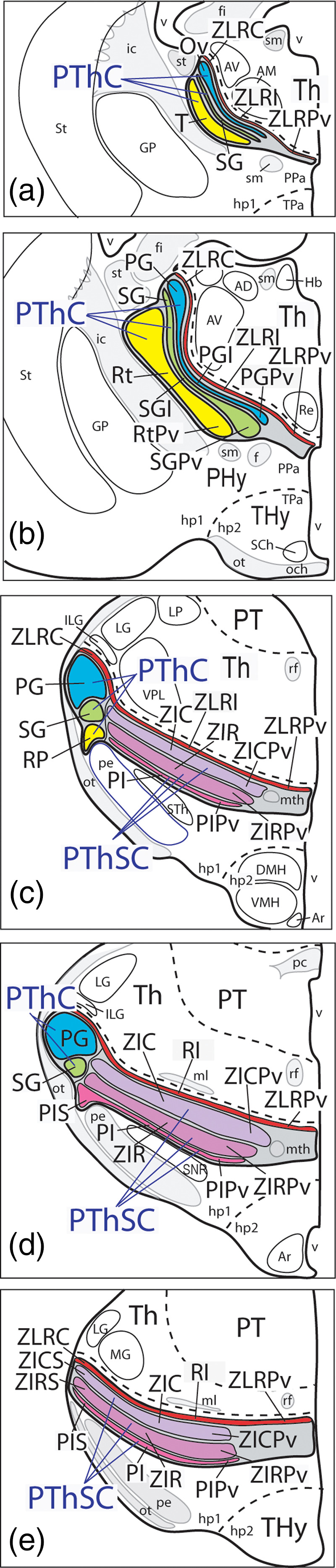
Color‐coded schemata of a dorsoventral series of horizontal sections through the prethalamus in the adult mouse. The schemata are based on a Gad67 ISH brain series, whose labelling is mainly restricted to the prethalamus in the area of interest. Dorsoventral, rostrocaudal y radial subdivisions of the central prethalamus (PThC) and subcentral prethalamus (PThSC) are highlighted in the context of the hypothalamic and thalamic neighbours. The color code is the same as in Figure 21. The midline lies to the right; caudal is oriented to the top. Interprosomeric limits are marked as thin black dash‐lines [Color figure can be viewed at wileyonlinelibrary.com]

In dorsal horizontal sections passing just under the fimbria and the PThE proper, the *oval nucleus* and the *triangular nucleus* appear lateral to the stria medullaris at the point where this tract passes from the PThE into the habenula. The Ov and T cover here the dorsal ends of the Rt, SG and PG nuclei (fi, PThE, Ov, T, sm, Hb; Figures 16a–f, 18a–g and 22a). The position of this dorsal central prethalamic complex found under the fimbria can be verified in sagittal sections (fi, Ov, T, Rt, SG, PG; Figures 17a–g, 19a–g and 20b). The superficial and retinorecipient *pregeniculate* and *subgeniculate* nuclei expand laterocaudally underneath these dorsal levels, covered superficially by the optic tract (PG, SG; ot; Figures 16b–k, 17a–d, 18b–k, 19a–e, 20a and 22b–d). Ov is distinguished from the PG by the higher compaction of its neurons (resulting in stronger *LacZ* signal), its typical position dorsal to the optic tract, and, crucially, *lack of the layered structure characteristic of the PG*, as seen in horizontal sections (compare Figures 16f,h and 18g,i). Indeed, the *pregeniculate nucleus*, found more ventrally, rostral to the thalamic geniculate nucleus and the intergeniculate leaflet, clearly shows its definitive superficial four‐layered structure, with cells predominating in the denser magnocellular (external o Layer 2) and parvocellular (internal or Layer 4) layers, which are separated by the cell‐poor Layer 3 (PG2‐4, LG, Figures 16g–k and 18i–k). The PG Layer 1, or marginal layer, represents the retinorecipient neuropile, which only has scattered blue cells; authors often include this layer in the magnocellular outer Layer 2 (PG1; Figures 16g–k and 18j,k). The PG as a whole is moderately labeled at P40, contrasting with denser *LacZ* signal at the unlayered SG (SG, PG; Figures 16i–k, 17a–d, 18h–k and 19a–e). This pattern is just the opposite of what was observed at earlier stages examined.

The intermediate stratum of the PThC region is considerably compressed between the thalamus and the hypothalamus. This stratum is mainly represented by the Rt, which forms a thick convex cap around the rostrolateral part of the thalamus, while SGI and PGI are apparently reduced to intercalated thin—perhaps discontinuous—rows of cells difficult to distinguish from Rt in this stretched material (Rt, SGI, PGI; Figures 16c–g, 17e–g, 18b–e, 19d–i, 20b and 22b). In the periventricular PThC, *LacZ* signal is stronger in the ovoid SGPv stratum than in the RtPV and PGPv, this being best observed in horizontal sections (RtPv, SGPv, PGPv; Figures 16b–f, 17b–d and 22b). This distinct labeling reminds of previous observations in *Dlx*‐*LacZ* sections counterstained with PV and *Isl1* labeling at earlier postnatal stages (PV, Figure 8; *Isl1*, Figure 9).

#### 3.7.3 Subcentral prethalamic region (PThSC)

The *preincertal subcentral formation* (PIPv; PI; PIS), jointly with the *rostral and caudal parts of the zona incerta* (ZIRPv; ZIR; ZIRS and ZICPv; ZIC; ZICS), represent additional radial prethalamic complexes which form a separate rostrocaudal series at the *ventral* end of the prethalamic alar domain, under the central prethalamus (Figures 16h–l, 17b–j, 18f–l, 19d–k, 20 and 22c–e). In sagittal sections, the whole incertal region seems to lie topographically caudal to the PThC, and ventral to the thalamic mass. As a whole, these subcentral formations appear limited rostrally and ventrally by the peduncle, before the latter turns from its *dorsoventral* peduncular hypothalamic course (rostral to PThE, PThC and PThSC) into its *longitudinal* course within the basal diencephalic tegmentum (Figure 1d,e); the basal hypothalamic subthalamic nucleus (STh) appears deep to the peduncle precisely at its topologic knee, a point which tends to be disregarded in the literature (Puelles, Martinez‐de‐la‐Torre, Bardet, et al., 2012; Puelles & Rubenstein, 2003, 2015). The STh accordingly is also a rostral neighbor of the incertal complex (STh; Figures 16h–k, 17d–g, 18i–l, 19e–g, 20b and 22c). Caudally the subcentral prethalamic region contacts the ZLR‐derived, *LacZ*‐ and *Nkx2.2*‐positive *retroincertal nucleus* (RI; Figures 16j,k, 17c–g, 18l,m, 19g–i, 20b and 22d,e). The latter lies close to the inflection of the medial lemniscus, where it departs from its prior longitudinal tegmental course to enter ventrodorsally across the CLi domain the ventrobasal thalamic mass, just caudally to the zona limitans (ZIR, ZIC, RI, ml, ZL,; Figures 16h–l, 17b–j, 18i–l, 19d–l, 20b and 22d,e).

Unlike at earlier postnatal stages, the *caudal zona incerta* (ZIC; seen under the PG nucleus) now shows a less marked *LacZ* expression than the *rostral zona incerta* (ZIR; seen under the SG nucleus), though the ventromedially placed *periventricular part of the caudal zona incerta*, as well as the neighboring *nucleus of the mamillothalamic tract*, display a relatively stronger signal (ZIC, ZICPv, MTh; mth; Figures 16g–l, 17d–j, 18f–l,19e–k, 20b,c and 22c,d). The weakly *LacZ*‐positive *preincertal nucleus* (PI) lies in front of the ZIR and ventral to the reticular complex (ZIR; PI; Rt; Figures 16j–l, 17e–g, 18j–l, 19e–h, 20b and 22c–e). The subcentral *superficial PI nucleus* (PIS), located at the caudal edge of the peduncle (under the central *retropeduncular nucleus*, RP) has a particularly weak *LacZ* signal (PIS, Figures 16j–l, 17b,c, 18j,k, 19d, 20a and 22d,e). The ZIRS, similarly as the ZIR, is compact and rather strongly labeled (ZIRS; Figures 16l, 17b,c, 18l and 19d), whereas ZICS, like the ZIC, is relatively less compact and shows a moderate *LacZ* signal (ZICS: Figure 18l,m). The respective periventricular subcentral strata show in general moderate LacZ signal, possibly somewhat stronger at the ZICPv, which appears fragmented by the passage of the mamillothalamic tract (PIPv; ZIRPv; ZICPv; mth; MTh; Figures 16g–k, 17h–j, 18f–l, 19i–k, 20c and 22c,d).

In addition, scattered *LacZ*‐positive neurons are still observed within the p3 tegmentum, dorsally to the substantia nigra and the ventral tegmental area. This topography corresponds to the classic *tegmental fields H1 and H2 of Forel* (“H” stands for “Haube,” “tegmentum” in German) (F; Figures 16l, 17k and 18k,l; Forel, 1877). Some dispersed *LacZ* cells are also found in the p2 tegmental region (Figure 17h–l).

#### 3.7.4 Zona limitans, rostral shell of zona limitans (ZLR) and RLi

The superficial small patch of intensely *LacZ*‐positive neurons ascribed to the zona limitans rostral shell (ZLR) which was previously constantly identified as ZLRC in the neighborhood of the pregeniculate nucleus (PG) becomes less distinct at late postnatal stages. Some of its cells apparently persist within the adult intergeniculate leaflet (IGL), which we accordingly believe contains fused ZLR (prethalamic) and ZLC (thalamic) derivatives, consistently with our *Nkx2.2* mappings described above (Figure 7). Part of these cells seem to incorporate after a short range tangential migration into the PG nucleus, possibly representing as well the local dispersed calbindin‐positive cell population (Figure 15f,i). CB‐positive cells were found at earlier stages within the IGL and PG (IGL, PG; Figure 13g,g′,h,h′). Sections passing through the subcentral zona incerta show a larger and deeper ventromedial derivative of the ZLR, the *retroincertal nucleus* (RI), which expands in front of the thalamic entry locus of the medial lemniscus, separated from the ZIC by a cell‐poor gap (RI, ZIC; ml; Figures 16j,k, 17c–g, 18l,m, 19g–i and 22d,e). The molecularly and histologically distinct retroincertal nucleus appears to have been lumped classically with the caudal zona incerta (the conventional “dorsal zona incerta”). It apparently also has been often misinterpreted as either the “peripeduncular nucleus” (a derivative of ZLC, as shown above with *Nkx2.2* ISH), or the “lateral terminal nucleus” of the basal optic pathway, both identified in rodent atlases just rostroventrally to the medial geniculate body (see Section 4). A packet of longitudinally descending *Dlx*‐*LacZ*‐positive fibers, which apparently originates from the RI and courses caudalwards close to the alar‐basal boundary, was visible already at early embryonic stages in sagittal sections. These fibers seem to terminate in a weakly *LacZ*‐positive neuropile within the so‐called “lateral part” of the substantia nigra compacta (Figures 16m–q and 18n–q).

## 4 DISCUSSION

By definition in the prosomeric model, the prethalamus (the ventral thalamus of Herrick's columnar model) is the alar territory of the diencephalic prosomere 3, found immediately rostral to the zona limitans (the mid‐diencephalic organizer) and caudal to peduncular alar hypothalamic domains (Puelles, 2018, 2019; Puelles, Martinez‐de‐la‐Torre, Bardet, et al., 2012; Puelles, Martinez‐de‐la‐Torre, Ferran, et al., 2012; Puelles & Rubenstein, 2003, 2015). Most prethalamic progenitor domains excepting the PThE produce neurons expressing *Gad65*/*67* and GABA transporters, that is, generate GABAergic projection and local circuit neurons. The PTh projections target various alar regions of the thalamus, pretectum and midbrain, as well as correlative tegmental regions (Jones, 2007). All prethalamic GABAergic domains express *Dlx* genes (Puelles & Rubenstein, 2003; Stühmer, Puelles, et al., 2002). Differentially, the PThE, an hyperdorsal alar subregion found next to the chorioidal roofplate, expresses *Tbr1* (Bulfone et al., 1995) and other differential markers (*Lhx5*, *Lhx9*, *Gdf10*; Shimogori et al., 2010) related to glutamatergic neurons, which provide input to the habenular nuclei via the stria medullaris (Turner et al., 2016). This transmitter‐related bipartition of the prethalamus recalls the subpallio‐pallial partition of the neighboring telencephalon, though here no significant mixing of excitatory and inhibitory populations occurs, at least in rodents. The prethalamus also contrasts with its caudal neighbor the thalamus, where mainly glutamatergic cells are produced in rodents, with partial invasion of extrinsic inhibitory interneurons (Jager, Calpin, Durmishi, Shimogori, & Delogu, 2019; Jager et al., 2016; Jeong et al., 2011; S. Martinez & Alvarado‐Mallart, 1989).

The prethalamic diencephalic territory has traditionally attracted less scientific attention than its thalamic and hypothalamic neighbors, leading at best to fragmentary anatomical and functional knowledge (concentrated on the reticular nucleus, zona incerta and some superficial visual nuclei), and at worst to considerable confusion about prethalamic boundaries and inner partitions, particularly when it was confused with the supposedly tegmental “subthalamus” (Forel, 1877). This latter trend caused erroneous ascription of some strictly prethalamic parts, when not the whole territory, to either the hypothalamus or the thalamus (see comments about the obsolete and confusing “subthalamus” concept in Puelles, Martinez‐de‐la‐Torre, Bardet, et al., 2012).

We felt that a recapitulative study of prethalamic structure performed with genoarchitectural markers might establish on a stronger basis a precise neuromeric model for this forebrain territory, hopefully, illuminating some misconceptions arisen in the wake of the now obsolete columnar approach (Puelles, 2018; Puelles, 2019; Puelles & Rubenstein, 2015).

Our present genoarchitectural study examines in detail postnatal and adult structure of the mouse prethalamus, starting by an analysis of subtle differences in *Dlx5*/*6*‐*LacZ* expression, and the surprisingly very informative correlation of such data with *Dlx1*/*2*/*5*/*6* immunochemical and in situ signals. We compared next the *Dlx* pattern with a handful of other prethalamic gene markers chosen because of their differential expression pattern within the area of interest. Irrespective that this report focuses on postnatal structure for the sake of manageability and general usefulness, our interpretations have been constantly informed by correlative prenatal developmental data at our disposal (our own collection of preparates, as well as the Allen Developing Mouse Brain Atlas, which we frequently consulted). In this report we used developmental material only at a few selected points of interest, for clarifying purposes.

Our results basically corroborate our earlier neuromeric studies of this forebrain region (Bulfone et al., 1993; Puelles & Rubenstein, 1993; Rubenstein, Martinez, Shimamura, & Puelles, 1994), but now we suggest an expanded genoarchitectural parcellation of the mouse prethalamus, contemplated in a model with partial intersection of four rostrocaudal and four dorsoventral domains (Figures 1, 6, 20 and 22). Due to the fact that three primary histogenetic compartments, namely PThE, RLi and ZLR, seem homogeneous (undivided), we distinguish on the whole 9 distinct progenitor domains in the prethalamus (3 central PTh + 3 subcentral PTh domains + PThE + RLi + ZLR). We did not explore the underlying prethalamic tegmentum. The central PTh is the largest prethalamic region and contains the best known large prethalamic structures, such as the reticular nucleus and the visual pregeniculate and subgeniculate nuclei. The subcentral PTh largely corresponds to the classic zona incerta, to which we added a third rostral component. Our study also underlines a prethalamic *radial structure*, previously scarcely visualized, which allows distinguishing between periventricular, intermediate and superficial strata, particularly within PThC and PThSC.

The perhaps unexpected level of complexity of the prethalamus in terms of patterned molecular and cellular regionalization establishes a more detailed scenario for future causal analysis of prethalamic patterning in terms of anteroposterior (AP) and dorsoventral (DV) molecular signaling systems. Major attention had been given before to the notion that the prethalamus essentially obeys in its molecular specification anteroposterior signals coming from the zona limitans, that is, the mid‐diencephalic secondary organizer (e.g., the morphogens SHH, WNT8b, WNT3a, FGF8; Crespo‐Enriquez, Partanen, Martinez, & Echevarria, 2012; Martinez‐Ferre et al., 2016; Puelles & Martinez, 2013; Scholpp & Lumsden, 2010; Scholpp, Wolf, Brand, & Lumsden, 2006; Vieira, Garda, Shimamura, & Martinez, 2005; Vieira & Martinez, 2006). Recent mouse results of Andreu‐Cervera et al. (2019) clearly reveal distinguishable DV and AP patterning effects on this forebrain area. Our present results suggest that additional prethalamus patterning influences potentially may arise from the hypothalamus, as well as from the roof and basal/floor plates of prosomere 3. An expansion of the habitual patterning scenario may be needed in order to achieve a full causal explanation of all the structures we see in the adult prethalamus.

### 4.1 Boundaries and morphogenetic deformation of the mouse prethalamus

The position of the prethalamus within the prosomeric model encompasses the whole alar plate of prosomere 3 (Figure 1; Puelles, 2018; Puelles & Rubenstein, 1993, 2003, 2015). As occurs with any well‐delimited, tridimensionally developing neuroepithelial sector, the adult prethalamus (PTh) represents a deformed cuboidal portion of the rostrodorsolateral diencephalic brain wall. The PTh displays free ventricular and pial surfaces which are topologically parallel to each other, irrespective of their respective final positions, much deformed during morphogenesis at the interface between the telencephalon and the thalamus. Classical neuroanatomy tended to disregard these inner and outer PTh surfaces, wrongly ascribing them either to the telencephalon or to the thalamus (figs. 10 and 11 in Puelles, 2019; and fig. 9 in Puelles, Martínez‐Marin, et al., 2019, aim to explain the sizeable morphogenetic deformations that cause this fundamental error, particularly in the human brain; such deformations are less marked in the mouse). We will explain below a minimum of needed facts about these free surfaces. There are in addition four other surfaces of the same cuboid; these limit with neighboring neuroepithelial domains in the remaining four directions of topological space (dorsal, ventral, rostral, and caudal).

The ventricular and pial PTh surfaces are strictly parallel to each other only at early neural tube stages, characterized by a simple neuroepithelial structure of the PTh (alar p3). During subsequent morphogenesis, the *ventricular surface* is importantly compressed rostrocaudally between the bulging thalamic mass at the back, and the peduncular hypothalamus (containing the massive peduncle) plus the associated telencephalon at the front (Figure 1d). Progressive differential growth of the adjacent hemisphere (enlargement of basal ganglia, plus formation of occipital and temporal poles) and correlative expansion of the thalamus and thalamo‐telencephalic connections (the latter necessarily have to navigate through the interposed PTh into the hemispheric stalk) jointly cause the prethalamic *pial surface* to become diverted caudalwards together with the thalamic and pretectal pial surfaces. In the human brain all of these lateral diencephalic regions finish facing the rostral midbrain under the pulvinar (Hochstetter, 1919); however, in the mouse the thalamic “lateral” and “medial” geniculate bodies still bulge *laterally* as rostrodorsal and rostroventral thalamic elements, respectively, even if hidden by the covering hemisphere; the pial face of the PTh is to be found immediately rostral to these landmarks. Accompanying this partial deformation, the superficial prethalamic derivatives partly embrace the alar hypothalamic sector of the cerebral peduncle (see fig. 8.12 in Puelles, Martinez‐de‐la‐Torre, Bardet, et al., 2012) thus allowing space for the flattened optic tract, which always courses caudalwards along the marginal alar PTh in its approach to the thalamus and pretectum (Puelles, Martinez‐de‐la‐Torre, Bardet, et al., 2012; fig. 8.34B; Wagner, McCaffery, & Dräger, 2000; Yonehara et al., 2009).^1^

In all adult mammals, the much reduced ventricular surface of PTh still separates the thalamus from the hypothalamo‐telencephalic complex; the ventricular PTh remnant lies topographically at or next to the prominent caudal limit of the interventricular foramen, where both PThE and PThC participate. Actually, depending on the species, a varying rostral part of PThE evaginates during development into the caudomedial wall of the hemisphere, where the true rostral boundary of PThE opposes intraventricularly the subpallial stria terminalis complex across the sulcus terminalis (Alonso et al., 2020; Puelles, 2019). This complicating aspect is not represented in Figure 1d schema. The PTh site at and across the interventricular foramen lies rostral (or far rostral in the human case) from the adult free pial surface of PTh (Figures 16e,f and 19g; Hochstetter, 1895; Schwalbe, 1880; see also fig. 11 in Puelles, 2019, and fig. 9 in Puelles, Martínez‐Marin, et al., 2019).

The *dorsal* PTh limiting surface is thin, and taenial in nature, since it contacts the prethalamic *roof plate*, represented by the p3 portion of the third ventricle chorioidal roof. The latter forms a cryptic (usually unrecognized) intermediate portion of the forebrain chorioidal roof which separates the analogous thalamic and telencephalic portions (Figure 1d; see also fig. 11c in Puelles, 2019). Our developmental observations indicate that the mouse prethalamic chorioidal roof plate lies just rostral to the velum transversum (we deduce this from the fact that the molecularly‐labeled interthalamic zona limitans ends dorsally at this landmark; see vt in Figure 1d). This finding implies that classic authors ascribed the cryptic prethalamic roof area to the telencephalon, since they generally believed the velum transversum to be a diencephalo‐telencephalic limiting landmark (Kuhlenbeck, 1973). The longitudinal boundary between the prethalamic roof and alar plates is visualized as a “taenia,” where the chorioidal tela attaches to the upper rim of the PThE. A longitudinal tract or a commissure may be associated to a taenia. In fact, the stria medullaris tract courses subpially through PThE next to the cited alar‐roof limit (see fig. 8.34B in Puelles, Martinez‐de‐la‐Torre, Bardet, et al., 2012); in some older works the stria medullaris itself was known as the “taenia tract” (Déjerine, 1895; Riley, 1960).

The opposed *ventral* limiting surface of the PTh cuboid represents in our model the longitudinal boundary of the p3 alar plate with the p3 basal plate (p3 tegmentum), that is, the *alar‐basal boundary* (Figure 1d). In the adult, this longitudinal border is found roughly in a coronal section plane, due to the axial bending occurred developmentally at the cephalic flexure; the diencephalic tegmentum characteristically contains many longitudinally coursing fiber packets (Weigert‐Pal‐stained fig. 86 in Ramón y Cajal, 1966). The p3 tegmentum lies topologically caudal to the hypothalamic retromamillary area (RM; Figure 1); it contains among other elements the rostralmost portion of the mesodiencephalic substantia nigra and ventral tegmental area complex underneath the prethalamic zona incerta subregion (Figures 4, 17 and 19; see fig. 10 in Puelles, 2019). The diencephalic alar‐basal limiting plane roughly covers the *longitudinal basal* course of the cerebral peduncle, which starts at p3 level just behind the basal hypothalamic subthalamic nucleus; (see, Ramón y Cajal, 1966; fig. 86), and separates it from the *longitudinal alar* diencephalic course of the optic tract (also at p3 level; see fig. 8.34 in Puelles, Martinez‐de‐la‐Torre, Bardet, et al., 2012). It is very uncommon in the literature to see represented pure transversal sections of p3 that illustrate the local alar‐basal relationships. Such sections, when theoretically conceived—for example, see fig. 10.4e in Puelles et al. (2013), or casually obtained (Figure 7k) do not coincide either with standard coronal or horizontal sections in brain atlases, which normally show the PTh sectioned obliquely.

The topologically *rostral* limiting surface of the PTh cuboid (which results deformed into a nearly lateral position, as mentioned above) bounds with the caudal telencephalon at levels through the interventricular foramen, as well as with the underlying alar peduncular hypothalamus at levels through the paraventricular area (PHy; Pa; Figure 1d). This is the surface which thalamo‐telencephalic fibers need to cross in order to connect with the internal capsule, forming the upper (alar) root of the cerebral peduncle (Puelles & Rubenstein, 2003, 2015). The peduncular hypothalamus contains in its intermediate and superficial strata the topologically dorsoventral hypothalamic course of the cerebral peduncle, that is, the medial and lateral forebrain bundles, respectively (Puelles, Martinez‐de‐la‐Torre, Bardet, et al., 2012; figs. 8.12 and 8.34). The medial forebrain bundle courses through the lateral hypothalamus (an intermediate hypothalamic stratum across both alar and basal plates of PHy), and this stratum is covered superficially by the lateral forebrain bundle (Puelles & Rubenstein, 2015; fig. 12). Obviously, the alar PTh relates only to the alar components of this hypothalamic territory (*loc.cit*.; and PTh; PHy; Figure 1d). The main periventricular rostral neighbor of PTh is the hypothalamic paraventricular nucleus, which is contacted by the PThE, the PThC and the PThSC (Puelles, Martinez‐de‐la‐Torre, Bardet, et al., 2012; figs. 8.09, 8.12, 8.13, 8.15, 8.17, 8.18, 8.20, 8.21, 8.24, 8.27A, 8.30, 8.31). The underlying subparaventricular area is caudally contiguous with the likewise *Dlx*‐positive RLi and ZLR complex (Figure 1d,e; we had to modify this notion, since in earlier accounts we wrongly interpreted that the incertal prethalamus was continuous with the subparaventricular hypothalamus). Dorsally to the paraventricular nucleus, the PThE limits rostrally with amygdalar and hippocampal telencephalic domains (Alonso et al., 2020; Puelles, Martinez‐de‐la‐Torre, Martinez, Watson, & Paxinos, 2019; figs. 4, 11 and 12).

Finally, the *caudal* limiting surface of the PTh cuboid bounds with the adult thalamus across the transverse glial palisade that develops at the site of the embryonic zona limitans (the latter roughly coincides with the classic “external medullary lamina”; e.g., Keyser, 1972). In embryos, the central core domain of the zona limitans, where *Shh* is strongly expressed in continuity with the underlying basal plate expression (Puelles, 2017, 2019; Puelles et al., 2004; Puelles & Martinez, 2013), is contoured rostrally (within PTh) and caudally (within thalamus) by thin bands of *Shh*‐negative neuroepithelium where some gene markers are selectively expressed in a shared pattern (e.g., *Nkx2.9*, *Nkx2.2* and *Ptc*; Echevarría, Vieira, & Martínez, 2001; Gimeno, Hashemi, Brûlet, & Martínez, 2002; Kitamura et al., 1997; Price et al., 1992; Puelles et al., 2004; Shimamura, Hartigan, Martinez, Puelles, & Rubenstein, 1995). We have previously referred to these bands as “rostral and caudal shell domains” of the zona limitans (ZL; ZLR; ZLC; see, e.g., Puelles, 2013; Puelles & Martinez, 2013). There are some gene markers which label selectively either the rostral or the caudal ZL shell domains, indicating the existence of partial molecular differences in their respective molecular profiles (e.g., Dbx1 only in the ZLR, and Six3 only in the ZLC; Jeong et al., 2011; S Martinez et al., 2012). The respective neuronal derivatives of ZL, ZLR, and ZLC (which have a tendency to migrate radially and tangentially) thus have differential properties. After radial migration into the corresponding mantle stratum, the mouse ZLR and ZLC derivatives usually perform short divergent tangential migrations to incorporate into neighboring prethalamic (e.g., PG) or thalamic (e.g., IGL) grisea (Delaunay et al., 2009; Delogu et al., 2012; Jeong et al., 2011; Vue et al., 2007; Vue et al., 2009). The primary ZLR and ZLC mantle formations accordingly are largely transient and possibly partially disappear as such at late embryonic and postnatal stages, excepting the so‐called “intergeniculate leaflet,” a persistent subpial retinorecipient formation held to be heterogeneous in its cell population. Some superficial cells derived from the dorsal ZLR or ZLC seem to migrate tangentially into the PG nucleus (see Figure 7, and Jeong et al., 2011; fig. 7A–C, where only‐red (*Nkx2*.2) and red‐green (*Nkx2.2*/*Tal1*) parts of IGL appear. Separate *Tal1*‐derived IGL and PG fluorescent cell patches are distinguished, while no prethalamic progenitors express *Tal1*). The ZL core domain forms itself the transverse palisade of radial glia cells mentioned above, which is crossed orthogonally by all thalamo‐telencephalic fibers (and viceversa), and may produce as well some mantle derivatives, mainly periventricular or intermediate (Kitamura et al., 1997). It may be accordingly understood that, in embryos, the caudal contact of PTh with the thalamus occurs across the glial ZL border and implies actually two transient parallel borders: the PThC‐PThSC versus ZLR limit, and the limit between the ZLR and the ZL core (something similar happens for the thalamus caudally to the ZL). This structural state should be kept in mind for patterning studies, but possibly is not as relevant for adult PTh structural analysis, other than as an explanation of the existence of some peculiar migrated cell types within the neighboring PTh or thalamus (thalamic GABAergic interneurons may originate from the *Ptc*‐ and *Nkx2.2*‐positive ZLC and CLi domains, unless they come really from the analogous, but *Dlx*‐positive ZLR; this point is not yet clear). It has been so far a matter of convention whether the ZL, ZLR and ZLC formations are ascribed or not to the PTh and Th; we propose ascribing ZLR to PTh, since this whole field is *Dlx5*/*6*‐positive and reacts to ZLCo‐derived rostrally directed gradiental SHH signals.

**FIGURE 23:**
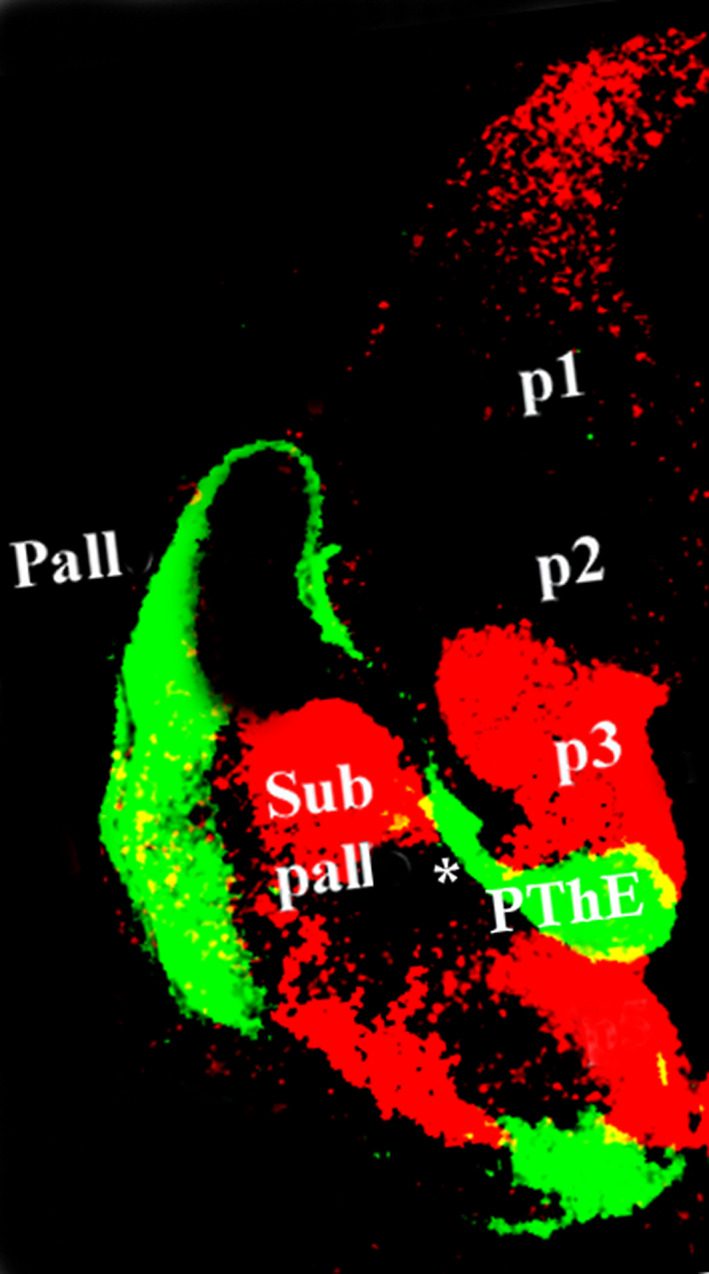
Graphically superposed adjacent horizontal sections through the diencephalon of an E13.5 mouse embryo. This pseudocolor darkfield image shows the relative expression patterns of two genes, *Dlx2* (in red; subpallium and noneminential prethalamus, p3) and *Tbr1* (in green; pallium and eminential prethalamus, PThE), obtained by separate in situ hybridization with mRNA probes. Note the *Tbr1*‐positiv*e* evaginated part of the PThE (asterisk) extends beyond the interventricular foramen as a flap added to the caudomedial wall of the evaginated telencephalic hemisphere (it covers the pial surface of p3). The flap ends at the unlabeled thin chorioidal fissure of the lateral ventricle opposite the fimbrial end of the hippocampal primordium. The evaginated PThE flap is separated from the *Dlx*‐positiv*e* subpallium by the compacted sulcus terminalis (also marked by the asterisk). Modified from Puelles (2001a) [Color figure can be viewed at wileyonlinelibrary.com]

As mentioned, the real shape of the deformed adult mammalian PTh is further complicated by the fact that a sizeable rostral part of the mammalian PThE results incorporated to the evaginating hemisphere across the interventricular foramen, bending around the local pial hemispheric sulcus as a fulcrum. This evaginated PThE portion ends in the form of a tapering flap that participates in the medial wall of the hemisphere, next to the sulcus terminalis (asterisk in Figure 23, modified from Puelles, 2001a; note the PThE flap has a Tbr1‐positive mantle, which is also characteristically calretinin‐positive, both of them being PThE markers) (see also Abbott & Jacobowitz, 1999). The evaginated part of PThE is usually misidentified in human textbook neuroanatomy as the *lamina affixa*, falsely believed to be *adhered* to the thalamus pial surface (see Puelles, 2019). Its ventricular surface within the lateral ventricle contacts the medial ganglionic eminence and eventually the amygdala and hippocampus along the sulcus terminalis, as well as the roof plate, which it must reach due to the hyperdorsal nature of the PThE, represented by the chorioidal fissure of the lateral ventricle (Puelles, 2019; Puelles, Martínez‐Marin, et al., 2019). The unevaginated part of the PThE becomes visible at the caudal contour of the interventricular foramen (that is where the “eminentia” makes its bulge) and behind it (Figures 1a,b,d,e,f and 23).

### 4.2 General genoarchitectural of the prethalamus

We turn now to overall molecular markers found at the PTh. The whole alar prethalamus, including the PThE, shares initially the expression of genes such as *Zic1* and *Zic5*, two zinc‐finger genes (Ferran, Puelles, & Rubenstein, 2015), or *Arx* (the PThE is *Arx*‐positive only at early stages, between E10.5 and E13.5, and restricted to its ventricular zone) (Allen Developing Mouse Brain Atlas; Cobos et al., 2005; Miura, Yanazawa, Kato, & Kitamura, 1997). In contrast, the basal prethalamus expresses, for instance, *Tcf4*, *Foxa1*, *Tle4*, *Lmx1b*, and *Ptx2* (Martinez‐Ferre & Martinez, 2012).

Various gene markers delineate the rostral and caudal boundaries of the prethalamus. We corroborated that the expression of *Dlx* genes (also *Arx*) ends caudally at the interthalamic ZL boundary, specifically at the limit between the *Dlx*‐positive ZLR and the *Shh*‐positive ZL core domain (Cobos et al., 2005; Jones & Rubenstein, 2004; Puelles & Rubenstein, 1993, 2003); *Arx* is distinctly expressed also in the ZLR (Puelles, Amat, & Martinez‐de‐la‐Torre, 1987). This caudal PTh limit is also clearly recognized by gene markers which are not expressed in the prethalamus, but in the adjacent thalamus, such as *Tcf7l2* and *Lef1*. These two genes are jointly expressed through alar midbrain, pretectum and thalamus, up to the said prethalamus/thalamus boundary (Ferran, Puelles, et al., 2015; Jones & Rubenstein, 2004; Nagalski et al., 2013; Nagalski et al., 2016; Allen Developing Mouse Brain Atlas). The caudal PTh boundary is also delimited by selective expression of *Semaphorin 5a* (*Sema5a*) and *Gbx2* in the alar thalamus (Jones & Rubenstein, 2004; Nakagawa & O'Leary, 2001; Puelles & Martinez, 2013). Other genes have an alar hypothalamic expression pattern that stops caudally at the hypothalamo‐prethalamic boundary; for example, *Cv2*, *Otp*, *Meis2*, *Rgs4*, *Sim1* and *Vax1* (Coffinier, Ketpura, Tran, Geissert, & De Robertis, 2002; Fan et al., 1996; Ferran, Puelles, et al., 2015; Hallonet et al., 1998; Morales‐Delgado et al., 2014; Morales‐Delgado et al., 2011; Puelles, Martinez‐de‐la‐Torre, Bardet, et al., 2012; Puelles & Rubenstein, 2003; Shimogori et al., 2010). The basal hypothalamic expression of *Otp*, *Sim1* and *Plagl1* also ends caudally at the transverse border with basal p3 (Ferran, Puelles, et al., 2015; Morales‐Delgado et al., 2014; Morales‐Delgado et al., 2011; Puelles et al., 2004; Puelles, Martinez‐de‐la‐Torre, Bardet, et al., 2012; Puelles & Rubenstein, 2003).

The PThE expresses differentially *Tbr1* and *Calb2* (calretinin) in its population of glutamatergic neurons (present results; Abbott & Jacobowitz, 1999; Bulfone et al., 1995; Puelles et al., 2000). A number of other developmental genes are also expressed selectively within the PThE: *Pax6* appears selectively at the eminential ventricular zone (as in the pallium) as well as *Gdf10*, while *Lhx1*, *Lhx5*, *Lhx9*, *Emx2* signals appear in the eminential mantle (present results; Abellán et al., 2010; Puelles et al., 2000; Shimogori et al., 2010).

Specific genes of the *Dlx* family are expressed selectively within PThC and/or PThSC domains and subdomains (see Figure 6), where they probably are related to the differentiation of specific subsets of GABAergic neurons. Shimogori et al. (2010; fig. 3) illustrated *Olig2* signal apparently restricted to PThSC, but correlative *Olig2* material found at the Allen Developing Mouse Brain Atlas suggests this expression extends also into PThC. As development advances, *Arx* expression tends to become restricted to the ZLR and PThSC subregions, in a pattern similar to that of *Pax6*.

*G*ain‐ or loss‐of‐function experiments have shown that the neurally expressed *Dlx* genes (paralogues 1,2,5,6) are required for the differentiation of GABAergic interneurons in the mouse telencephalon (Anderson, Eisenstat, Shi, & Rubenstein, 1997; Anderson, Qiu, et al., 1997; Long, Cobos, Potter, & Rubenstein, 2009; Long et al., 2007; Stühmer, Anderson, et al., 2002; Stühmer, Puelles, et al., 2002; Wang et al., 2011). As would be expected according to the observed *Dlx* patterns, in rodents the adult PThC and PThSC regions express both *Gad2* (*Gad65*) and *Gad1* (*Gad67*), which encode two isoforms of glutamic acid decarboxylase, the enzyme responsible for the production of γ‐amino butyric acid (GABA); they also express vesicular GABA transporters *vGat* and *Slc32a1* (Nagalski et al., 2016; Yuge et al., 2011). The thalamus instead contains relatively few GABAergic cells, except some produced at the midbrain (Jager et al., 2016), and others generated either at the ZLC, the *Dlx*/*Pv*‐positive Rt primordium, or another more distant *Dlx5*/*Pv*‐expressing progenitor domain, from where they migrate tangentially into the Th (Jager et al., 2016; this report prefers for reasons unclear to us a subpallial origin of the thalamis Dlx/Pv interneuronal lineage). Of course, the thalamus also expresses massively *vGlut2*, which encodes an isoform of the vesicular glutamate transporter (Puelles, Martinez‐de‐la‐Torre, Ferran, et al., 2012; Yuge et al., 2011). DLX and GAD65 proteins have nearly overlapping patterns in the prethalamus at embryonic stages (see fig. 2 in Stühmer, Puelles, et al., 2002). *Dlx1*/*Dlx2* double knockout mice have reduced prethalamic signal of *Gad65* and *Gad67* mRNA isoforms (figs. 6i C,F and 6iii c,d,g in Le et al., 2017). Moreover, the molecular association of *Dlx* genes with prethalamus is evolutionarily ancient. An homotopic lamprey prethalamus homolog could be first mapped precisely thanks to DLX immunoreaction and relevant prosomeric assumptions (Martínez‐de‐la‐Torre, Pombal, & Puelles, 2011). A significant prethalamic mantle layer containing GABAergic young neurons, jointly with an unlabeled partially evaginated PThE area, were illustrated in a stage 26 shark larva by Carrera, Ferreiro‐Galve, Sueiro, Anadón, and Rodríguez‐Moldes (2008; fig. 1B). Moreover, specification of GABAergic cells depending on DLX transcription factors was demonstrated in zebrafish (MacDonald et al., 2013).

### 4.3 Inner subdivisions of the prethalamus

#### 4.3.1 Dorsoventral subzones: PThE, PThC, PThSC

We propose a dorsoventral subdivision of the prethalamus in four longitudinal components: (a) *prethalamic eminence*—PThE—as a dorsal region; (b) a subjacent *central prethalamic zone*—our PThC—which includes the reticular, subgeniculate and pregeniculate nuclei; (c) a ventral *subcentral prethalamic region*—our PThSC, which contains the zona incerta and preincertal formations, and (d) limiting with the basal plate region, a thin *rostral alar liminar region* (RLi) expressing the gene *Nkx2.2* (among others); this region is a longitudinal extension of the interthalamic ZLR band, as a phenomenon typically induced by very high levels of SHH morphogen, which occurs either at the ZLR, in front of the ZL core, or at the RLi, along the alar‐basal boundary (Puelles, 2013; Puelles, Martinez‐de‐la‐Torre, Bardet, et al., 2012; Puelles, Martinez‐de‐la‐Torre, et al., 2019; Puelles & Rubenstein, 2015; Shimogori et al., 2010; see Figures 2 and 10). This tetrapartite dorsoventral PTh subdivision does not precisely match the alar organization of the thalamus, as contemplated within our prosomeric approach; the latter consists of five dorsoventral pronuclear units (epithalamus, dorsal, intermediate and ventral thalamic tiers, plus the possibly subparafascicular thalamic CLi domain; (Dávila, Guirado, & Puelles, 2000; Díaz et al., 1994; González, Puelles, & Medina, 2002; Martínez‐de‐la‐Torre et al., 2002; Puelles, 2001a, 2013; Puelles, Martinez‐de‐la‐Torre, et al., 2019; Redies et al., 2000). Moreover, the alar hypothalamus displays a dorsoventral alar organization into only two contrasting longitudinal domains, the paraventricular and subparaventricular areas (Morales‐Delgado et al., 2011; Puelles, Martinez‐de‐la‐Torre, Bardet, et al., 2012; Puelles, Martinez‐de‐la‐Torre, et al., 2019). As indicated above, the PThE mantle massively expresses TBR1, which also appears expressed less densely, though slightly more ventrally, in the central and ventral parts of the alar hypothalamic paraventricular area (Figure 1c). However, the chicken data of Alonso et al. (2020; also unpublished experimental fate‐mapping data) suggest that the paraventricular *Tbr1*‐positive cells are tangentially migrated eminential ones.

In contrast, *Dlx* family genes are strongly expressed in the alar hypothalamus only at the subparaventricular area, which occupies the ventral part of the alar hypothalamus (Figure 1b). This domain is relatively thick dorsoventrally in the terminal hypothalamus (site of the anterior hypothalamic and suprachiasmatic nuclei) and rather thin across the peduncular hypothalamus (THy; PHy; Figure 1b). We have concluded that the latter intensely *Dlx*‐positive mantle population is caudally continuous with the intensely *Dlx5*/*6‐LacZ*‐positive RLi domain of the prethalamus (not with the zona incerta, as reported previously in several publications). Accordingly, the PThSC region limits rostrally with the ventral part of the hypothalamic paraventricular nucleus complex in our updated model (VPa; Figure 1d,e; Puelles, Martinez‐de‐la‐Torre, Bardet, et al., 2012). In its turn, the PThC limits rostrally with the central part of the paraventricular nucleus (CPa). These two borders of PTh with VPa and CPa are selectively traversed longitudinally at lateral hypothalamus level by the thalamocortical fibers entering the internal capsule after they have crossed the reticular nucleus (and by other fibers in contrary direction). Note the PThE above this locus only *seems* to relate rostrally with the DPa, or with the nearest subpallium, since a virtual extension of the interventricular foramen leads around the protruding PThE into the terminal sulcus; early embryos clearly show the foraminal relationship, which becomes difficult to see in postnatal brains (compare Figure 1a,b,d–f, as well as Figure 22). Massive development of the subpallial medial ganglionic eminence leads to its occlusion of the interventricular foramen (Figure 1c), though the PThE still extends into its evaginated flap incorporated into part of the caudomedial hemispheric wall. We believe that the true rostral border of the deformed PThE probably relates to the amygdala and ventrocaudal hippocampus, as can be observed at early developmental stages (Alonso et al., 2020; fig. 24). The *Dlx*‐expressing PThC and PThSC prethalamic regions which appear intercalated between PThE and RLi accordingly represent extra alar domains without counterpart in the alar hypothalamus.

Experimental studies have shown that diencephalic dorsoventral regionalization is generated by antagonistic effects of dorsalizing and ventralizing inductive signals diffusing simultaneously from the roof plate (mainly TGF‐β‐related morphogens such as BMP4 and BMP7, and WNT family members) and the floor plate (SHH), respectively (Basler, Edlund, Jessell, & Yamada, 1993; Dickinson, Selleck, McMahon, & Bronner‐Fraser, 1995; Echelard et al., 1993; K. J. Lee & Jessell, 1999; Liem, Tremml, Roelink, & Jessell, 1995; Shimamura et al., 1995; Shimamura, Martinez, Puelles, & Rubenstein, 1997). Some peculiarities in the direct or indirect effects mediated by these or ancillary molecules which are causally related to segmental (AP) differential molecular identity may cause the singular emergence and increased relative growth of the PThC and PThSC histogenetic fields, and, ulteriorly, further expansion of its mature nuclear derivatives. This hypothesis needs to be explored experimentally. The roof plate atop the PThE was recently proposed as a forebrain signaling center based on the expression of BMPs, WNTs, and FGFs morphogens. “PThE” transplanted heterotopically next to ventral telencephalic cells led to an ectopic expression of *Lef1*, a target gene of the WNT/β‐catenin pathway (Adutwum‐Ofosu, Magnani, Theil, Price, & Fotaki, 2016). Curiously, we have not observed systematic differences in the molecular profile of the PTHC and PThSC regions; on the contrary, molecular AP divisions observed at PThC tended to continue ventrally into corresponding AP divisions of PThSC (see Table 4). Nevertheless, the nuclear derivatives of these fields are structurally characteristic in many ways, as evidenced by classical anatomic schemata, all of which differentiate the zona incerta from the reticular and pregeniculate nuclei (the intercalated subgeniculate central domain is less well known). This suggests that we may not yet have identified enough of the genes that code for dorsoventral prethalamic architectonic differences within the *Dlx*‐positive territory.

**TABLE 4:** Molecular profile of the dorsoventral, rostrocaudal, and radial prethalamic subdivisions

	Rostral				Middle			Caudal			
	Superf	Interm	Periv		Superf	Interm	Periv	Superf	Interm		Periv
	RP	T	Rt	RtPv	SG	SGI	SGPv	PG	Ov	PGI	PGPv
PThC	Early *Dlx5*/*6*	Early *Dlx5*/*6*	***Dlx1*/*2***	***Dlx1*/*2***	Early *Dlx5*/*6*	**Early *Dlx5*/*6***	**Early *Dlx5*/*6***	*Dlx6*	***Dlx6***		Early *Dlx5*/*6*
	***Six3***	***Six3***	***Six3***	***Six3***			*Six3*				
	PV	PV	PV		PV	PV	PV				
	*Isl1*	*Isl1*	*Isl1*	*Isl1*	*Isl1*	*Isl1*	*Isl1*				*Isl1*
				*Pax6*	***Pax6***	***Pax6***	***Pax6***				*Pax6*
					*Enc1*			*Enc1* (internal layer)		*Enc1*	*Enc1*
			CB				CB				
	***Sst***	***Sst***	***Sst***					***Sst* (PGmc)**	***Sst***		
	***Ecel1***	***Ecel1***	***Ecel1***	***Ecel1***				***Ecel1***		***Ecel1***	***Ecel1***

	PIS	PI	PIPv	ZIRS	ZIR	ZIRPv	ZICS	ZIC	ZICPv
PThSC	***Dlx1*/*2***	***Dlx1*/*2***	***Dlx1*/*2***	*Dlx1*/*2*	**Early *Dlx5*/*6***	**Early *Dlx5*/*6***	*Dlx1*/*2*	***Dlx6***	***Dlx6***
		***Six3***							
						CB			
				***Pax6***	***Pax6***	***Pax6***			
				*Enc1*	*Enc1*	*Enc1*	*Enc1*	*Enc1*	*Enc1*
	***Sst***	***Sst***		*Sst*			***Sst***		
	***Ecel1***	***Ecel1***	***Ecel1***				***Ecel1***	***Ecel1***	***Ecel1***

*Note*: Markers that are expressed in both central and subcentral prethalamic subdivisions (PThC + PThSC) are highlighted in bold. PThC and PThSC are dorsoventral prethalamic subdivisions. Interm, intermediate; Periv, periventricular; Superf, superficial strata.

#### 4.3.2 Rostrocaudal radial domains: Rt, SG, PG

No one has proposed previously an anteroposterior subdivision of the prethalamus in three subregions (actually four, if the *Nkx2.2*‐expressing ZLR is included; Figures 20 and 22). Our results so far suggest this partition is restricted to the PThC and PThSC, since the PThE always appeared unitary, and the same applies to the RLi.

The Rt, SG and PG domains of PThC are tri‐stratified radial histogenetic complexes with periventricular, intermediate and superficial strata. They appear clearly ordered from rostral, next to hypothalamus, to caudal, next to the thalamus (Figures 20 and 22); remarkably, the characteristic *rostral* Rt element of PThC was arbitrarily characterized developmentally as a caudal entity in the rat by Altman and Bayer (1979a, 1979b, 1979c, 1988a, 1988b, 1995), who interpreted Rt as a direct derivative of the zona limitans interthalamic border; indeed, they repeatedly identified our ZL as “reticular eminence,” irrespective that the ZL has no direct developmental relationship whatsoever with the Rt primordium (other than as a source of morphogens). Comparative data on Rt shown by Díaz et al. (1994) in a lizard and Puelles, Martinez‐de‐la‐Torre, et al. (2019) in the chick corroborate a rostral separate position of Rt relative to the ZL, as observed here in the mouse; this error of Altman and Bayer probably causes persistent confusion in students of the interthalamic region.

The subjacent PThSC region is likewise subdivided in rostral, middle and caudal subregions, which represent tri‐stratified preincertal (PI), ZIR, and ZIC radial complexes, which are completed caudally by the corresponding nonfused ventral part of the ZLR, identified by us as retroincertal nucleus (RI) (Figures 20 and 22). As mentioned, these incertal subregions tend to share molecular properties with the respective overlying PThC units, including, for example, the *Dlx* forms expressed postnatally (see Figure 6 and Table 4). We nevertheless deduce from their known anatomical and hodological differences that PThC and PTHSC must be differentiated by as yet undiscovered genetic determinants.

Earlier studies, including our own ones, had distinguished only rostral and caudal prethalamic AP subdivisions, based on the preferent rostral expression of *Six3*, *Pax6* and calbindin (*Calb1*) in the rostral part of the prethalamus, and of *Dlx* genes caudally, at embryonic stages of the mouse and chick (Martinez‐Ferre & Martinez, 2012; Puelles et al., 2000; Puelles & Martinez, 2013; Puelles, Martinez‐de‐la‐Torre, et al., 2019; Puelles & Rubenstein, 2003). A similar bipartite PTh pattern was deduced from immunostaining studies in amphibians (Gonzalez, Lopez, Morona, & Moreno, 2017; Morona & González, 2008).

According to present results, in situ expression of *Six3* appears restricted largely to the *rostral* central and subcentral prethalamus (i.e., mainly at intermediate radial levels, at the reticular and preincertal nuclei; Figure 11; Table 4). The *rostral* and *caudal* central and subcentral domains are selectively distinguished from the corresponding *middle* domains by *Ecel1* signal (Figure 14; Table 4). This contrasts with *Pax6*, which is expressed superficially mainly at the subgeniculate nucleus and the underlying ZIR, which belong to the *middle* PThC domain, as observed in material from the Allen Developing Mouse Brain Atlas from embryonic stage E13.5 onwards (Figures 10 and 21; Table 4). *Pax6* expression typically also persists as a thin transverse band in the perinatal and adult periventricular stratum of PThC and PThSC. On the other hand, the *caudal* PThC/PThSC subregion can be distinguished by its relatively much stronger *Dlx6* expression within the PG and ZIC formations, consistently with the stronger *Dlx*‐*LacZ* signal in our material, a demarcation reinforced also by locally restricted *Ecel1* expression (Figures 2, 5, 6 and 14; Table 4).

Experimental studies have shown that some molecular anteroposterior regionalization of the prethalamus depends on morphogens secreted by the zona limitans, the mid‐diencephalic secondary organizer (Echevarría, Vieira, Gimeno, & Martínez, 2003; Kiecker & Lumsden, 2004; Scholpp et al., 2006; Szabo et al., 2009; Vieira et al., 2005; Vieira & Martinez, 2006; Vue et al., 2009; Zeltser, 2005). SHH and WNT morphogens (WNT8b, WNT3a) are released by the ZL core, and these reportedly diffuse rostrally and caudally in the neighboring alar diencephalon to establish via a position‐dependent reaction pattern the differential AP molecular profiles of the prethalamus and thalamus. The concentration gradients of these morphogens (jointly with intrinsic sensitivity properties of the genome) presumedly enable local tissue to select differential AP fates (S Martinez et al., 2012; Parr, Shea, Vassileva, & McMahon, 1993; Puelles, 2017; Puelles & Martinez, 2013; Roelink & Nusse, 1991; Scholpp & Lumsden, 2010; Vieira et al., 2005). Our present data suggest that such secondary AP patterning actually occurs in 3–4 analogous steps across the PThC and PThSC parts of the prethalamic alar plate (4 steps counting the ZLR; the SGL would represent an added AP singularity). A possible modulation of this pattern by differential DV signals, including ventralizing ones from the basal/floor plates, or by other AP signals theoretically diffusing from the hypothalamus, has not been explored yet.

#### 4.3.3 Zona limitans shell: Rostral shell domain (ZLR) and rostral liminar domain (RLi)

Both the rostral and caudal shell domains of the zona limitans (ZLR, ZLC), which develop at high SHH signaling level next to the *Shh*‐ and *Otx2*‐positive zona limitans core (ZLCo), express *Nkx2.2*, *Nkx2.9*, and *Ptc*, among other markers, some of which are selective for ZLR or ZLC (Delogu et al., 2012; Echevarría et al., 2001; Gimeno et al., 2002; Golding et al., 2014; Kitamura et al., 1997; Martínez‐de‐la‐Torre et al., 2002; Price et al., 1992; Puelles et al., 2004; Shimamura et al., 1995; Virolainen, Achim, Peltopuro, Salminen, & Partanen, 2012). These thin histogenetic centers are associated to the development of various thalamic or prethalamic derivatives, including the so‐called “intergeniculate leaflet,” a retinorecipient entity, some of whose cells migrate tangentially either caudally into the thalamic *posterior limitans nucleus* or rostrally into the prethalamic PG nucleus; (Delogu et al., 2012; Jeong et al., 2011); other deeper ZLR/ZLC derivatives were tentatively identified here on the basis of selective *Nkx2.2* expression as the prethalamic RLi and RI, and the thalamic PP and CLi (Figure 7). The mature cell populations selectively derived from the prethalamic ZLR are still poorly known, in contrast to the better studied ZLC derivatives (Delogu et al., 2012; Jeong et al., 2011). We have observed *Nkx2.2*‐positive and CB/*Calb1*‐positive elements within PG. These surely include those that migrate tangentially from the thalamic intergeniculate leaflet (ZLC), but may contain as well prethalamic derivatives of the similarly *Nkx2.2*‐positive ZLR (fig. 6 in Kitamura et al., 1997; present results; see our Figure 13g′,h). The ZLR expresses selectively *Dlx* family genes, as well as *Pax6*, *Arx*, *Lhx1* and *Dbx1*, whereas differential genes such as *Tal1*, *Sox14*, *Six3*, *Npy* and *Calb2* appear selectively at the ZLC (present results; Delogu et al., 2012; Jeong et al., 2011; S Martinez et al., 2012). We think that the ventralmost (and broadest) part of ZLR forms the retroincertal nucleus (RI), a deep aggregate lying next to the medial lemniscus tract as it penetrates the ventrobasal thalamus; the RI is a newly defined entity (RI in our Figures 3, 4, 5, 6, 7 and 16, 17, 18, 19).

A thin longitudinal band expressing *Dlx5*/*6*‐*LacZ* extends beyond the ZLR proper under the PThSC, overlapping the upper edge of the prethalamic and hypothalamic basal plate expression domain of *Shh*. This band is identified here as the ventralmost alar prethalamus as the *rostral liminar band* (RLi), and it shows like the ZLR/ZLC complex a *Nkx2.2*/*Ptc*‐positive molecular profile (Puelles, Martinez‐de‐la‐Torre, Bardet, et al., 2012). RLi is held to be due to analogous patterning conditions as the ZL‐shell bands, namely the local existence of particularly high SHH levels (secreted by both basal and floor plates); these bands disappear entirely when notochordal ventralization of the neural tube is compromised (Andreu‐Cervera et al., 2019). Practically nothing is known about adult RLi derivatives, though we have found at least two candidates, which seem distinct from both the incertal/preincertal complex (PThSC) and the RI nucleus, and are restricted topographically to the ventralmost part of PTh. One of them is represented by a population of partly disaggregated *Dlx*‐ and *Pax6*‐positive cells, which surround the origin of the mamillothalamic tract out of the princeps mamillotegmental tract. This *Pax6*‐positive population was previously identified in the literature as the “*nucleus of the mamillothalamic tract*” (Skidmore et al., 2012; Szabó et al., 2011; Valverde et al., 2000).

Another neighboring aggregate of *Dlx*‐positive cells found near the prethalamic alar‐basal border, as well as near the hypothalamo‐prethalamic limit, lies practically in an entopeduncular position precisely where the cerebral peduncle bends from its dorsoventral hypothalamic course into its longitudinal tegmental diencephalic trajectory. This small cell group was identified by Ramón y Cajal (1903) as the “accessory subthalamic” nucleus. However, it does not share molecular markers with the subthalamic nucleus proper (which expresses, e.g., *Calb2* and glutamatergic markers), whereas the aggregate in question expresses strongly *Dlx*‐*LacZ*, *Sst*, *Ecel1*, and *Arx* (present results, Allen Developing Mouse Brain Atlas), and probably contains accordingly GABAergic neurons. Given its entopeduncular hypothalamic position and its gene markers, similar to those of the ventral entopeduncular nucleus, we have chosen to name it the “*accessory entopeduncular nucleus*.” We ignore, however, whether this mass is hodologically and functionally related to the main dorsal and ventral entopeduncular nuclei of the peduncular hypothalamus (Wallace et al., 2017).

### 4.4 Nuclear derivatives of the prethalamus in historic perspective

This is the first detailed and molecular description of the full regionalization of the prethalamus in rodents. Previous genoarchitectural studies in rodents followed older neuroanatomic literature in only identifying the largest prethalamic nuclei, notably the reticular and pregeniculate nuclei (the old “ventral lateral geniculate nucleus”), as well as the zona incerta (Bluske, Kawakami, Koyano‐Nakagawa, & Nakagawa, 2009; Jones & Rubenstein, 2004; Nagalski et al., 2016; Nakagawa & O'Leary, 2001; Vue et al., 2007; Yuge et al., 2011).

#### 4.4.1 Prethalamis eminence (PThE)

The PThE was classically identified as a “thalamic eminence” in the developing diencephalon of anamniotes and amniotes (Gilbert, 1935; Herrick, 1910, 1936; Keyser, 1972; Kuhlenbeck, 1927, 1954). This structure is now easily recognizable by the specific expression of calretinin (*Calb2*; Abbott & Jacobowitz, 1999), *Tbr1* (Bulfone et al., 1995), *Lhx1*, *Lhx5*, and *Lhx9* (in the mantle) (Abellán et al., 2010; Shimogori et al., 2010), and several other markers, notably *Pax6*, which is restricted to its ventricular stratum (review in Puelles & Martinez, 2013; Shimogori et al., 2010; present results). The PThE is presently conceived as a distinct hyperdorsal progenitor domain of the prethalamic (p3) alar plate, which is found next to the prethalamic chorioidal roof plate, and partly evaginates into the caudomedial wall of the hemisphere in mammals (Alonso et al., 2020; fig. 11; Puelles, 2018, 2019; Puelles & Rubenstein, 2003). As a result, part of its ventricular surface bulges at the back of the interventricular foramen (that is the historic reason for its “eminence”). Its evaginated continuation *apparently* limits with the medial ganglionic eminence (along the terminal sulcus), though its primordial neighbors are the pallial amygdala and the hippocampus, as well as the chorioidal fissure; the evaginated PThE has been speculatively misinterpreted in the human brain as the *lamina affixa* (see Puelles, 2019). The nonevaginated PThE mantle contains the longitudinal course of the stria medullaris (coming from the alar hypothalamus); this tract later penetrates the thalamic habenular territory, reaching the habenular commissure. Adult derivatives of the PThE are not described in detail in mammalian literature (with the possible exception of the bed nucleus of the stria medullaris), due to its notable mediolateral stretching and anteroposterior compression between local morphogenetic deformations generated by the thalamus and the telencephalo‐hypothalamic complex (see Puelles, 2019; figs. 10 and 11b,c; Puelles, Martínez‐Marin, et al., 2019; fig. 9). The apparent progressive “vanishing” of the PThE also relates to the existence of various tangential migrations of PThE cells into hypothalamic and telencephalic regions (Abellán et al., 2010; Alonso et al., 2020; Huilgol et al., 2013; Meyer, 2010; Meyer, Perez‐Garcia, Abraham, & Caput, 2002; Roy, Gonzalez‐Gomez, Pierani, Meyer, & Tole, 2014; Ruiz‐Reig et al., 2017; Takiguchi‐Hayashi et al., 2004; Tissir et al., 2009).

As a consequence of its partial evagination, the PThE pial surface is bisected longitudinally by the hemispheric sulcus, which therefore is not a proper limiting sulcus (as opposed to the interpretation of Kuhlenbeck, 1973, who regards it as the external tel‐diencephalic limit). Our present in situ observations of *Calb2* expression in the adult mouse revealed the persistence of some *Calb2*‐positive cell aggregates around the stria medullaris tract at the expected locus of the PThE (Figure 15). These aggregates collectively represent the stretched *bed nucleus of the stria medullaris* (BSM of the literature). Recent work in both mouse and chick suggests that the apparent developmental reduction of the PThE cell population is due to several massive migrations which transfer sizeable PThE cell populations rostralwards to a number of hypothalamic, subpallial, pallial and septocommissural forebrain sites (Alonso et al., 2020; Ruiz‐Reig et al., 2017; Watanabe et al., 2018). Interestingly, these variously migrated PThE populations all share with the BSM a glutamatergic phenotype, *Calb2* expression, and projections to the habenula.

#### 4.4.2 Central prethalamus (PThC)

We will comment on its superficial, intermediate and deep periventricular derivatives, each of which may be ascribed to either rostral, middle or caudal molecularly distinct compartments.

**Central superficial stratum (RP, SG, PG and Ov)**: The three major superficial elements of the PThC, that is, the retropeduncular (RP), subgeniculate (SG) and pregeniculate (PG) nuclei are disposed in a rostrocaudal row under the PThE, deep to the optic tract (Figures 20 and 22). The RP, SG and PG share retinal input, as was recently illustrated by anterograde labeling studies with cholera toxin B subunit in the mouse (Gaillard, Karten, & Sauvé, 2013; review in Monavarfeshani, Sabbagh, & Fox, 2017; Morin & Studholme, 2014, e.g., fig. 1K—note the RP was wrongly identified as “PP” or “peripeduncular nucleus” by these authors). Confusingly, these experimental data newly establishing RP and SG as prethalamic retinorecipient nuclei lying rostral to the PG were not acknowledged by either Sefton, Dreher, Harvey, and Martin (2015) or Sabbagh et al. (2018). Adding to the confusion, Monavarfeshani et al. (2017) depicted the PP proper as retinorecipient in fig. 1, but did not include the RP in the list of retinorecipient structures.

The thalamic PP, possibly first identified in front of the medial geniculate nucleus by Saper, Swanson, and Cowan (1976), and later shown at this site in rodent brain atlases by Hof et al. (2000), Watson and Paxinos (2010), and Paxinos and Franklin (2013), needs to be distinguished strictly from the prethalamic RP, a wholly different entity which lies just caudal to the peduncle at levels *above* the zona incerta complex. Faull and Mehler (1985) held that PP belongs to the PTh, without giving its exact derivation. Paxinos and Franklin (2013) do not identify the RP at this position, though it is visible as an AChE‐negative subpial gray formation lying just rostral to the AChE‐positive SG (figs. 50–34, 121, 146). The conventional PP is commonly mapped more ventrally and caudally than the RP, typically rostral to the medial geniculate body, and close to the tegmental substantia nigra. We conclude that the RP relates to the peduncle next to the alar hypothalamus whereas the PP, which we think derives from the thalamic ZLC, relates to the peduncle as it passes through the thalamic tegmentum (that is, PP is found after the peduncle turns in a right angle around the tegmental subthalamic nucleus; RP lies above this turning point; Figure 20; Puelles, Martinez‐de‐la‐Torre, Bardet, et al., 2012; Puelles & Rubenstein, 2015). We accordingly estimate that both Nakagawa and O'Leary (2001) and Nagalski et al. (2016) referred to the parabasal PP identified within prosomere 2 (thalamus) in their molecular mappings, rather than to the more dorsal alar prethalamic RP within rostral PThC.

It is unclear whether PP also receives retinal projections (Morin & Studholme, 2014). A different retinorecipient entity, the lateral terminal nucleus (“LT”) is sometimes mapped next to the PP (Morin & Studholme, 2014; Paxinos & Franklin, 2013), though we regard this topographic ascription as probably erroneous, since we conceive the true LT as a *pretectal* formation associated to the transverse peduncular tract. This name seems to have been carelessly applied to some other retinorecipient spot next to the zona limitans, without precise indication whether it belongs to PTh or Th.

Comparative considerations suggest that a small and normally cryptic *oval nucleus* (Ov) may be added to the list of superficial retinorecipient prethalamic grisea present in mammals (see Morin & Studholme, 2014; fig. 1G,H; labeling dorsal to the lateral geniculate and PG projections, or superficial to Rt). Ours results are the first genoarchitectural demonstration of Ov as a distinct *Sst*‐positive entity in the mouse. We propose to apply the descriptive reptilian name “oval” to mammals (as we did before for the avian positional homolog; Puelles, Martinez‐de‐la‐Torre, et al., 2019; Puelles, Martinez‐de‐la‐Torre, Paxinos, Watson, & Martinez, 2007). Topologic homology is complemented in this case by existence of a small separate retinorecipient *prethalamic* nonlayered formation (not projecting to telencephalon), which lies in all cases dorsal to the PG and the optic tract, as previously recognized in amphibians (the nucleus of Bellonci; see Puelles, Milán, & Martínez‐de‐la‐Torre, 1996), reptiles (original Ov concept; Huber & Crosby, 1926) and birds (classic “lateral anterior nucleus,” often wrongly ascribed to the thalamus; Ehrlich & Mark, 1984b; Huber & Crosby, 1929; Repérant, 1973; Webster, 1974). An old account postulating the possible existence of this entity in mammals was recorded by Addens (1938). The Ov nucleus is normally elusive in mammals because, excepting *Sst*, it does not stain differently than the PG or other prethalamic nuclei with most markers studied. It corresponds in such material to the small subpial stratum where the Rt nucleus seems to reach the brain surface, dorsally to the PG. Nissl stain locates there an aggregate of small neurons. Rt neurons are not likely to reach the pial surface, irrespective of appearances, because Rt is strictly an *intermediate stratum* component of PThC. In any case, the mouse Ov does appear differentially labeled by *Sst* compared to the mainly *Sst*‐negative PG (Figure 12b,c), and it lies as predicted in a retinorecipient dorsal extension of the PG territory (see retinal projections at this locus in Morin & Studholme, 2014, fig. 1G,H, though the locus was not identified by these authors; see also review and discussion of Ov concept in Puelles, Martinez‐de‐la‐Torre, et al., 2019; Puelles et al., 2007). The avian Ov also shows selective *Sst* expression (Ferran, Puelles; unpublished observations).

The comparative literature on the prethalamus in reptiles suggests an antecedent of our RP in the reptilian “suprapeduncular nucleus” described by Frederikse (1931) and Papez (Papez, 1935). Huber and Crosby (1926) distributed much the same territory among a pair of prethalamic nuclei, which they named “ventromedial” and “ventrolateral” nuclei, respectively. The “suprapeduncular” name refers, in *columnar* interpretation, to a position “dorsal” to the hypothalamic course of the peduncle, which was wrongly thought to be “ventral” in the old columnar model (the relationship is actually caudorostral); this nucleus would thus correspond to our “retropeduncular” element in prosomeric interpretation. The alternative names “ventromedial + ventrolateral nuclei” also refer to apparent *columnar* position in coronal sections, this time relative to the supposedly “dorsal” (actually “caudal”) thalamic mass (Butler & Northcutt, 1973; Cruce, 1974; Huber & Crosby, 1926; Senn, 1968; Shanklin, 1930). These authors did not identify specifically a Rt‐homolog population within the “ventral thalamic” (prethalamic) diencephalic area. This notion was first introduced by Pritz and Stritzel (1990). Diaz et al. (1994) later discussed and provided additional hodological data on the reptilian Rt homolog, which proved to be associated to a radially complete rostral prethalamic territory (like our rostral PThC). This complex was composed of a superficial *suprapeduncular nucleus* (our RP); this element was clearly distinct from the adjacent, but more caudal, superficial *ventrolateral nucleus* (corresponding to our present SG nucleus). Deep to the SP/RP there was the classic *ventromedial nucleus* (correlative to our Rt), which seemed to end in a periventricular formation, the *dorsal hypothalamic nucleus* (corresponding to our present RtPv). Diaz et al. (1994) defined systematically reptilian Rt‐like neurons by their characteristic bidirectional and topographically ordered Rt‐Th connectivity. The whole mediolateral Rt prethalamic complex (equivalent topologically to our present RtPV, Rt proper and RP in mouse) projected in a topographically ordered fashion upon the three dorsoventral tiers of thalamic structure (Díaz et al., 1994) (compare mappings of these tiers in Dávila et al., 2000; Martínez‐de‐la‐Torre et al., 2002; Puelles, 2001b; Redies et al., 2000). Dávila et al. (2000) subsequently also illustrated some lizard suprapeduncular neurons labeled retrogradely from the thalamus, which clearly mapped just caudal to the alar hypothalamic sector of the peduncle in a sagittal section (fig. 5E,F). Diaz et al. (1994) study thus in fact already demonstrated in a lizard a rostrocaudal sequence of prethalamic superficial centers (SP; VL; ventral geniculate nucleus, or PG; see fig. 1) which must be homologous with our present RP, SG and PG superficial units within mouse PThC. We substituted the RP (retropeduncular) term for the earlier SP name following our standing program to abandon obsolete columnar references whenever possible, substituting descriptive terms consistent with the prosomeric forebrain axis. The “retropeduncular” descriptor is more precise than SP, which might be confused with the parabasal “peripeduncular” (PP) term.

The RP within rostral PThC shows in our hands selective PV‐, *Isl1*‐, *Six3*‐, *Ecel1*‐, and *Sst*‐expression, a profile that is shared with the deeper Rt cell population. The RP cells differ from Rt cells by their higher expression of *Dlx5*/*6‐LacZ* and lack of calbindin (CB) (Table 4). In the cat, a RP nucleus located consistently with our definition was identified as a superficial extension of the *Wisteria floribunda* agglutinin (WAF)‐positive and SMI32‐positive Rt population (Baldauf, 2010; figs. 1B, D and 3A–C).

The superficial SG nucleus was apparently first recognized in the rat as a separate AChE‐positive entity by Paxinos and Watson (1986; these authors also coined this term). As mentioned above, the SG was identified as a retinorecipient nucleus by Morin and Studholme (2014), but not by Sefton et al. (2015). Selective AChE labeling distinguishes the SG not only from the PG, but also from our RP (see our discussion above), which is also AChE negative (Paxinos & Franklin, 2013). The SG also stands out among neighboring superficial prethalamic nuclei in the mouse by its relatively strong *Enc1* and *Pax6* staining, and it is selective lack of *Ecel1* expression (Allen Developing Mouse Brain Atlas; present results: Figures 10, 12 and 14; Table 4); we found that SG and the underlying ZIR tend to show similar molecular profiles. Other markers such as PV and *Isl1* also label the SG, but are shared with portions of the reticular/RP complex (present results: Figures 8 and 9; Table 4).

Based on comparable topology and connections, SG is the homolog of the classic sauropsidian and amphibian ventrolateral nucleus (e.g., reptiles, Bass & Northcutt, 1981; Derobert et al., 1999; Kenigfest et al., 1997; Medina & Smeets, 1992; Reiner, Zhang, & Eldred, 1996) (birds, Ehrlich & Mark, 1984a; Ehrlich & Mark, 1984b; Inzunza & Bravo, 1993; Marín et al., 2001; Norgren & Silver, 1989) (amphibians, Montgomery & Fite, 1989; Wye‐Dvorak, Straznicky, & Tóth, 1992). It accordingly was duly proposed to use this term also in birds (implicitly likewise in reptiles) to replace the previous “ventrolateral nucleus” name, which has clearcut wrong columnar connotations (Puelles, Martinez‐de‐la‐Torre, et al., 2019; Puelles et al., 2007); of course, “subgeniculate” is also columnar‐inspired—and strictly wrong, topologically, since it actually is a “pre‐pregeniculate” entity rather than “subgeniculate,” but this term is clumsy, and we thought it preferable to keep the known topographic SG name for the sake of clarity. Consistently with retinal input to the SG in rodents (Gaillard et al., 2013; Morin & Blanchard, 1999; Morin & Studholme, 2014) a prethalamic SG (ventrolateral nucleus) receives also retinal projections in birds (Ehrlich & Mark, 1984a, 1984b; Norgren & Silver, 1989) and reptiles (Bass & Northcutt, 1981; Butler & Northcutt, 1978).

In the hamster, SG receives also afferent inputs from the thalamic intergeniculate leaflet and projects ipsilaterally to its prethalamic PG neighbor (old ventral lateral geniculate nucleus) and bilaterally to the intergeniculate leaflet (Morin & Blanchard, 1999).

The PG, jointly with the correlative dorsal oval nucleus (Ov), forms the caudal major superficial component of the central prethalamic subregion. The prethalamic laminar PG was previously known as “ventral lateral geniculate nucleus” (Hines, 1929) based on its location with respect to the thalamic dorsal lateral geniculate nucleus, with the intercalated IGL formation, and its retinorecipient connectivity. The terms *ventral* and *dorsal* derived from the obsolete columnar model, and correspond to topological prosomeric rostral and caudal locations. Accordingly, the names pregeniculate (PG) and lateral geniculate (LG) nuclei seem appropriate for these two entities. Both nuclei have differential development, cytology, immunohistochemistry, and pattern of connections (review in Monavarfeshani et al., 2017). In addition to a different prosomeric origin (PG derives from p3 versus LG from p2), the PG has a *Dlx*‐positive profile reflecting its GABAergic character, whereas the LG contains mainly glutamatergic neurons and only few GABAergic cells (Gabbott & Bacon, 1994; Harrington, 1997; Inamura, Ono, Takebayashi, Zalc, & Ikenaka, 2011; Puelles, Martinez‐de‐la‐Torre, Ferran, et al., 2012; Sabbagh et al., 2018; Yuge et al., 2011). Moreover *Htr2c* (a serotonin receptor) and *Cdh6* are restricted to PG, whereas *Sert*, *Nr1d1*, *Gfrα1* and *p57kip2* markers label specifically LG (Yuge et al., 2011).

Respect to their connections, there are differences in the subtypes of retinal ganglionic cells that innervate PG and LG nuclei and in the properties of their retinal synapses (Monavarfeshani et al., 2017). Retinal inputs innervate massively the magnocellular external PG layer, which is *Enc1*‐ and *Htr2c*‐negative in contrast with the nonretinal inner layer, which is *Enc1*‐ and *Htr2c*‐positive (Yuge et al., 2011; present results; Table 4). Retinal and nonretinal layer cells of PG also express differentially Cat315‐ and WFA‐extracellular matrix proteins, respectively, in contrast with LG cells, which do not express either of these molecules (Sabbagh et al., 2018). Nonretinal inputs to the PG are more diverse and mainly different that those to LG, including sources such as the superior colliculus, visual cortex, and several pretectal and rhombencephalic nuclei (review in Monavarfeshani et al., 2017; see figs. 3 and 4). Visual cortical cells project to both PG and LG, but layer V cells innervate PG whereas layer VI cells project to LG in rodents (Bourassa & Deschênes, 1995; Cosenza & Moore, 1984; Hammer et al., 2014; Jacobs et al., 2007; Seabrook, El‐Danaf, Krahe, Fox, & Guido, 2013). PG efferents are also more diverse that those of LG, innervating regions related with visuomotor function, eye movement, vestibular function, and circadian function, including the superior colliculus and the hypothalamic suprachiasmatic nucleus (Matute & Streit, 1985; Moore, Weis, & Moga, 2000; Taylor, Jeffery, & Lieberman, 1986). In sharp contrast to the LG, the PG does not project to the visual cortex, or any other cortical region (Harrington, 1997).

**Central intermediate stratum (T, Rt, SGI, PGI[Ic])**: The reticular nucleus (Rt) is strictly the rostral, main component of the PThC intermediate stratum, though the literature often ascribes to the Rt other central intermediate populations belonging to the SGI and PGI strata. The Rt domain is labeled selectively by *Six3* at perinatal and postnatal stages. The middle and caudal subregions of the PThC, containing the SGI and PGI cell populations, separate the Rt from the thalamus. The homologous reticular nucleus population in lizards and crocodilians, which suffers less morphogenetic deformation than in mammals, has been clearly illustrated as a *rostral* PTh subregion (Díaz et al., 1994; Pritz & Stritzel, 1990), which consistently shows GAD and PV expression (Pritz, 2018).

The practically *Dlx5*/*6*‐*LacZ*‐negative Rt is covered dorsally by a *Dlx5*/*6*‐*LacZ*‐positive triangular population which can be also distinguished with respect to Rt by a strong *Sst*‐ and *Six3*‐expression and nonexpression of CB (present results; Table 4). The reptilian *triangular area* has a similar location, dorsal to the reticular nucleus, and caudal to the hypothalamic course of the forebrain peduncle (Ariens Kappers, Huber, & Crosby, 1936; Butler & Northcutt, 1973; Cruce, 1974; Huber & Crosby, 1926; Knapp & Kang, 1968a, 1968b; Medina et al., 1990; Papez, 1935; Repérant, 1973; Senn, 1968; Senn & Northcutt, 1973; Trujillo & Lopez, 1977). Accordingly, we propose to name this part of the reticular complex, the *triangular nucleus* or *area* (T). The T corresponds, at least partially, to the “rostral pole of the reticular nucleus” of columnar terminology (but is topologically dorsal). This Rt part is characterized by medium‐ to small‐size neurons with multipolar‐arranged dendrites, which contrast with the large fusiform cells and flat dendrite arborizations that characterize the Rt proper (cat, ferret, Clemence & Mitrofanis, 1992; cat, Scheibel & Scheibel, 1966; rat, Spreafico, Battaglia, & Frassoni, 1991). In the rat, the rostral pole of the reticular nucleus projects largely to the thalamic intralaminar (central‐lateral, paracentral, central‐medial, parafascicular) and midline (reuniens/rhomboid, parataenial) nuclei (Kolmac & Mitrofanis, 1997), whereas the efferent projections of the Rt proper target specific sensory dorsal thalamic nuclei (Pinault, Bourassa, & Deschênes, 1995a, 1995b). Thalamic efferent projections from the reptilian area triangularis are also found in the lizard *Varanus* (Hoogland, 1982). However, other connectivities studied in this area provide contradictory results. For instance, visual afferents to T are reportedly found in some turtles (Belekhova, 1979; Knapp & Kang, 1968a, 1968b) but not in most of studied reptilian groups (Bass & Northcutt, 1981; Butler & Northcutt, 1971, 1978; Künzle & Schnyder, 1983; Repérant, 1973). Trigeminal afferents are found in snakes (Molenaar & Fizaan‐Oostveen, 1980); however, nonbrainstem projections were detected in *Varanus* (Hoogland, 1982).

The term “nucleus reticularis,” previously named “Gitterschicht” (“grid layer”) by Nissl (1889), was introduced by Münzer and Wiener (1902) and corresponds to our Rt proper (possibly including as well our RP, T and RtPv), also known as the “main body of the reticular nucleus” (Clemence & Mitrofanis, 1992). These authors distinguished three cytoarchitectonic subregions in the reticular nucleus of cats and two in ferrets: a main body, an inner small‐celled part and a perireticular nucleus. The main body of their Rt corresponds largely to our Rt proper, whose cells are GABAergic and mostly immunoreactive to PV in all studied mammals (review in Jones, 2007; Mikula, Manger, & Jones, 2008). Moreover, the Rt contains somatostatin and calbindin‐positive cells (SST/Sst and CB/*Calb1*) (Clemence & Mitrofanis, 1992; Mitrofanis, 1992; present results; Table 4). The expression of genes such as *Arc*, *Kcnj4*, *Lancl3*, *Rnf144b*, *Tiam2*, *Trh*, *Fgd5* (Nagalski et al., 2016), *Meis2* and *Deinh2* (Zeisel et al., 2018) appears also restricted to the Rt.

The disperse cells of the perireticular nucleus are immersed in the hypothalamic course of the peduncle, that is, lie within the *Dlx*‐negative paraventricular peduncular hypothalamic subdomain, which lies just rostral to the *Dlx*‐positive prethalamic RP/Rt complex. The perireticular cells are also GABAergic (GABA‐ and DLX‐positive elements) and share with the prethalamic RP/Rt complex PV and SST (Clemence & Mitrofanis, 1992; present results), and other markers such as *Isl1*, *Six3* and *Ecel1* (present results). These data might support a prethalamic origin of these cells, assuming a subsequent migration to the paraventricular hypothalamic subdomain, as was previously suggested (Earle & Mitrofanis, 1996; Mitrofanis, 1994; Puelles, Martinez‐de‐la‐Torre, Bardet, et al., 2012).

The Rt has an important role as a modulator of the excitatory interactions between the cerebral cortex and the thalamus. Subregions of this GABAergic nucleus project, mainly ipsilaterally, to distinct thalamic nuclei. Moreover, the Rt receives topographically ordered innervation from collaterals of traversing glutamatergic thalamocortical and corticothalamic axons (Coleman & Mitrofanis, 1996; Crabtree, 1998; Guillery, Feig, & Lozsádi, 1998; Pinault et al., 1995a, 1995b; Pinault & Deschênes, 1998). Based on electrophysiological studies, the mammalian Rt may be subdivided in at least seven segregated sectors, five of them sensory, one motor and one limbic, with some overlap between these sectors (review in Pinault, 2004; Sokhadze, Campbell, & Guido, 2019). Electrophysiological properties of neurons obviously vary depending on their location in the Rt (S. H. Lee, Govindaiah, & Cox, 2007). Neuronal typological heterogeneity has been noted also in the Rt (e.g., a majority of PV‐expressing cells vs. minor subpopulations of CB‐ and STT‐expressing cells).

The neighboring narrow intermediate strata of the SG‐ and PG‐related radial complexes (SGI, PGI) are difficult to differentiate from the Rt in *Dlx5*/*6*‐*LacZ* material, but *Enc1*‐ and *Ecel1*‐expression distinguishes PGI from its *Isl1*‐ and PV‐positive SGI neighbor, and both *Six3* and *Sst* label transiently a thin caudal lamina of SGI (the SGL) abutting on the unlabeled PGI (present results; Table 4). These entities are not identified in mammalian neuroanatomical literature, where they usually are all ascribed to the Rt. In contrast, a sparsely populated PTh sector lying caudal to the Rt and rostral to the interthalamic limit, was clearly recognized in lizards as an intercalate area (Díaz et al., 1994); it may correspond to the sum of our SGI and PGI.

**Central periventricular stratum (RtPv, SGPv, PGPv)**: The *Pax6*‐expressing periventricular stratum of the PThC subdivides in three rostrocaudal parts based on distinct profiles, RtPv (*Ecel1*‐positive), SGPv (PV‐positive and *Ecel1*‐negative), and PGPv (*Enc1*‐ and *Ecel1*‐positive). Paxinos and Watson (2014) identified in a deep periventricular position their *paraxiphoid nucleus* (PaXi), which appears intercalated between the thalamus (reuniens and xyphoid nuclei) and the hypothalamic paraventricular nucleus. However, we believe that this nucleus is a derivative of the PThE, since it has a CR‐positive profile (present observations).

#### 4.4.3 Subcentral prethalamus (PThSC)

**Subcentral superficial stratum (PIS, ZIRS, ZICS)**: The superficial derivatives of PThSC had never been described before. We searched for them, since the known ZI portions (PI, ZIR, ZIC) all are components of an intermediate stratum, so that it could be predicted that some superficial cell populations might be associated to them. We succeeded best in visualizing PIS, ZIRS and ZICS in our doubly stained preparations with *Dlx5*/*6*‐*LacZ* reaction and pan‐DLX immunoreaction. They showed all three a pure brown labeling, which indicates restricted *Dlx1* expression, whereas deeper intermediate incertal elements showed various amounts of blue labeling.

**Subcentral intermediate stratum (PI, ZIR, ZIC)**: The preincertal nucleus (PI) is also described here for the first time. It corresponds to the rostral intermediate PThSC component, which lies strictly under the central Rt nucleus, with which it shares some markers (*Six3*, *Sst*, *Ecel1*; Table 4). In double *Dlx5*/*6*‐*LacZ* reaction and pan‐DLX immunoreaction, the PI likewise appears as a brown‐reactive mass, indicative of selective *Dlx1* expression. Due to its rostral position, the PI limits directly with the subparaventricular peduncular alar hypothalamus.

The zona incerta (ZI) was originally defined in myelin‐stained preparations as a scarcely stained area situated between large tegmental fiber tracts (Forel, 1877). Later several authors incorporated the ZI under the concept of “subthalamus,” jointly with other formations such as the subthalamic nucleus and the dorsal hypothalamic nucleus (review in Puelles, Martinez‐de‐la‐Torre, Bardet, et al., 2012; here reasons were considered to regard the confuse “subthalamus” concept as obsolete). Jones and Burton (1976) suggested that this neuronal population should be named “the nucleus of the zona incerta,” but this name has not been used. The whole domain is GABAergic and cyto‐ and chemo‐architectonically heterogeneous, with extensive afferent connections mainly from the thalamus, hypothalamus, brainstem, and spinal cord (review in Mitrofanis, 2005). The classic ZI is presently subdivided in three rostrocaudally ordered aggregates (PI/ZIR/ZIC). ZIR and ZIC were conventionally identified under columnar assumptions as “dorsal” and “ventral” parts of the zona incerta, although they are respectively rostral and caudal parts in a neuromeric topological conception (ZIC, ZIR). We added here the rostral preincertal nucleus (PI) as a further somewhat cryptic component of the PThSC (see above; Table 4). Kawana and Watanabe (1981) distinguished two other ZI sectors. One was held to lie rostrally (in fact, it was topologically dorsal, i.e., corresponded to the intermediate stratum of PThC—i.e., RtPV/SGPv/PGPv). The other was described as a caudal pole part; in our model it would seem to represent the area of ZIC closest to the ZLR and RLi, if not referring to the RI nucleus.

**Subcentral periventricular stratum (PIPv, ZIRPv, ZICPv)**: The changing molecular profiles of this rather small prethalamic region, not studied by most earlier authors, are presented in Table 4.

#### 4.4.4 Rostral shell of the zona limitans (ZLR) and RLi

We already commented above about the conceptual problems posed by this peculiar prethalamic primordium and its derivatives, in the context of relevant literature.

### 4.5 Conclusions

We think that our updated molecular and topologic conception of the prethalamus may be instructive for causal navigational explanations of the thalamo‐telencephalic connections, since the latter necessarily must be guided through the heterogeneous prethalamus (namely, its three AP parts, aside of its DV subdivisions). Prethalamic molecular heterogeneity suggests a more complex scenario for prethalamic patterning than has been contemplated so far. Our new model also provides new light upon the still insufficiently understood diencephalo‐telencephalic boundary (Alonso et al., 2020; Puelles, 2019). Another scenario for further prethalamic studies includes exploring the role of the diverse retinorecipient grisea distinguished in our prethalamic schema (visuomotor or other functions), in comparison, for instance, with those of pretectal and midbrain visual centers.

## Data Availability

The data that support the findings of this study are available from the corresponding author upon reasonable request.

## Endnote

1 Note the clearcut dorsoventral retinal projection pattern upon the diencephalon demonstrated by means of transport of a LacZ‐labeled retinoic‐acid reporter by Wagner et al. (2000); by means of transport of a LacZ‐labeled retinoic‐acid reporter shows throughout PTh, thalamus and pretectum an inverted DV topography which is only consistent with the prosomeric model (and our present prethalamus model), but cannot be explained within the antagonic diencephalic columnar model. One of the strongest points of the prosomeric model which is not fully realized in the field is that it makes sense of the spatial organization of retinotopic projections (see also Marín, Blanco, & Nieto, 2001). This implies that growing axons somehow detect and react to serial neuromeric fields and boundaries when they form synaptic connections.
